# Genome-wide expression profiling of aquaporin genes confer responses to abiotic and biotic stresses in *Brassica rapa*

**DOI:** 10.1186/s12870-017-0979-5

**Published:** 2017-01-25

**Authors:** Md. Abdul Kayum, Jong-In Park, Ujjal Kumar Nath, Manosh Kumar Biswas, Hoy-Taek Kim, Ill-Sup Nou

**Affiliations:** 1 Department of Horticulture, Sunchon National University, 255 Jungang-ro, Suncheon, Jeonnam 57922 South Korea; 2 University-Industry Cooperation Foundation, Sunchon National University, 255 Jungang-ro, Suncheon, Jeonnam 57922 South Korea

**Keywords:** Aquaporin, Abiotic stress, Biotic stress, Gene expression, *Brassica rapa*

## Abstract

**Background:**

Plants contain a range of aquaporin (AQP) proteins, which act as transporter of water and nutrient molecules through living membranes. AQPs also participate in water uptake through the roots and contribute to water homeostasis in leaves.

**Results:**

In this study, we identified 59 *AQP* genes in the *B. rapa* database and Br135K microarray dataset. Phylogenetic analysis revealed four distinct subfamilies of *AQP* genes: plasma membrane intrinsic proteins (PIPs), tonoplast intrinsic proteins (TIPs), NOD26-like intrinsic proteins (NIPs) and small basic intrinsic proteins (SIPs). Microarray analysis showed that the majority of PIP subfamily genes had differential transcript abundance between two *B. rapa* inbred lines Chiifu and Kenshin that differ in their susceptibility to cold. In addition, all *BrPIP* genes showed organ-specific expression. Out of 22 genes, 12, 7 and 17 were up-regulated in response to cold, drought and salt stresses, respectively. In addition, 18 *BrPIP* genes were up-regulated under ABA treatment and 4 *BrPIP* genes were up-regulated upon *F. oxysporum* f. sp. *conglutinans* infection. Moreover, all *BrPIP* genes showed down-regulation under waterlogging stress, reflecting likely the inactivation of AQPs controlling symplastic water movement.

**Conclusions:**

This study provides a comprehensive analysis of AQPs in *B. rapa* and details the expression of 22 members of the *BrPIP* subfamily. These results provide insight into stress-related biological functions of each *PIP* gene of the AQP family, which will promote *B. rapa* breeding programs.

**Electronic supplementary material:**

The online version of this article (doi:10.1186/s12870-017-0979-5) contains supplementary material, which is available to authorized users.

## Background

Plants depend on the absorption of water from soil and its subsequent transport to all other plant parts. Water moves inside the plant body through apoplastic, transcellular, and symplastic pathways. The symplastic pathway transports water across membranes [1] and is generally mediated by members of an ancient family of water channels called aquaporins (AQPs), which are part of the major intrinsic protein (MIP) superfamily [2]. Efficient cell-to-cell water movement through the plant is controlled by AQPs in different physiological contexts [3]. In addition to water uptake into roots, AQPs also function in water homeostasis in leaves [4, 5]. Moreover, AQPs are involved in controlling water movement for tissue expansion [6, 7] and have regulatory roles in processes such as fruit development [8] and cell enlargement in *Arabidopsis thaliana* roots, hypocotyls, leaves, and flower stems [6], and ripening of grape berries [9].

AQPs are predicted to consist of six membrane-spanning segments with two cytoplasmic termini. AQPs contain Asn-Pro-Ala (NPA) motifs located in two short, fold-back alpha helices following the second (loop B, LB) and fifth (loop E, LE) trans-membrane helices. Each AQP monomer contains two hemi-pores, which fold together to form the water channel. *Arabidopsis* encodes 35 different AQPs [10], whereas there are 66 AQPs in *Glycine max* [11], 31 in *Zea mays* [12], 33 in *Oryza sativa* [13], 54 in *Populus trichocarpa* [14] and 47 in *Solanum lycopersicum* [8]. Based on sequence similarity and subcellular localization, higher plant AQPs have been classified into five subfamilies, namely the plasma membrane intrinsic proteins (PIPs), the tonoplast intrinsic proteins (TIPs), the NOD26-like intrinsic proteins (NIPs), the small basic intrinsic proteins (SIPs), and the X (or unrecognized) intrinsic proteins (XIPs) [15]. The NIP subfamily is named for the founding member, soybean (*Glycine max*) nodulin-26 (*Gm*NOD26), which is an abundant AQP expressed in the peribacteroid membrane of N_2_-fixing symbiotic root nodules. It was initially thought that the NIP proteins were found only in the nodules of nitrogen-fixing legumes [16]. However, NIP proteins were later found in many non-leguminous plants including *Arabidopsis* [17], and rice [13]. The SIP subfamily is conserved in all plant species, but is not well characterized to date. The XIPs form a phylogenetically distinct subfamily and have been found in moss, fungi and dicot plants [15]. *Arabidopsis* encodes 35 different AQPs [10], 66 AQPs in *Glycine max* [11], 31 in *Zea mays* [12], 33 in *Oryza sativa* [13], 54 in *Populus trichocarpa* [14] and 47 in *Solanum lycopersicum* [8].

AQPs also appear to be involved in responses to abiotic stresses like drought, salt, and cold stresses in various plants. Seven members of the PIP1 subfamily of rice are responsive to cold stresses [18]. Moreover, *Tricticum aestivum* TIP2 regulates the responses of plants to abiotic stresses (salt and drought) via an ABA-independent pathway(s) [19]. In *Arabidopsis*, *PIP2;5* is up-regulated during cold exposure, and PIP subfamily genes are responsive to drought and salt stresses [20]. In addition, *NtAQP1* is involved in improving water use efficiency, hydraulic conductivity, and yield production under salt stress in tobacco [21]. By contrast, there is limited information whether AQPs function plant defenses against biotic stresses like attacks from fungal, bacterial and viral pathogens.

In this work, we carried out a genome-wide expression profiling of the *AQP* gene family in *Brassica rapa* to characterize which genes were responsive to biotic and abiotic stresses. *Brassica rapa* is a species of the genus *Brassica*, which is economically important worldwide. We performed comprehensive *in silico* analyses of gene classifications, chromosomal distribution, synonymous and non-synonymous substitution rates, syntenic relationships, evolutionary divergence, subcellular localization, gene duplication, phylogenetic analysis, exon–intron organization, conserved motifs, and predicted functions of AQPs in *B. rapa*. We further determined the gene expression pattern of PIP subfamily members in *B. rapa* plants in response to abiotic stresses (cold, drought, salinity, water logging) and ABA treatment. We also analyzed PIP subfamily expression under biotic stress (infection with *Fusarium oxysporum* f.sp. *conglutinans*), and assessed AQP protein similarity to stress response-related proteins from other plants.

## Results

### Identification and in silico functional analysis of *B. rapa* aquaporin genes

To identify all *AQP* genes in *B. rapa*, we searched SWISSPROT of the BRAD (http://brassicadb.org/brad/) [22] and annotations of microarray data for cold-treated *B. rapa* (Chiifu & Kenshin), removing any duplicates. A total of 61 gene sequences encoding putative members of the AQP family were identified in *B. rapa*. Domain searches using SMART confirmed that 59 of the putative *AQP* genes in *B. rapa* encoded predicted MIP and trans-membrane domains. In agreement with this result, protein sequence similarity analysis of all 61 sequences using blastp (protein-protein BLAST) showed that all but the two protein sequences lacking functional MIP and trans-membrane domains were most similar to proteins of AQPs. Based on these findings, we concluded that there are 59 functional *AQP* genes in *B. rapa*, which we named based on nomenclature used in other plants and guided by sequence similarity and phylogenetic analysis. Tao et al. [23] previously reported 53 *AQP* genes in *B. rapa*, and our analysis found these, along with six more *AQP* genes. Additional file 1: Table S1 lists the chromosomal position, ORF length and orthologous genes, as well as predicted protein length, iso-electric point and molecular weight for each of these 59 *B. rapa AQP* genes. These 59 AQP proteins of *B. rapa* showed a high level of sequence similarity to AQP proteins from different plant species. *In silico* functional analysis showed that the six newly identified *AQP* genes are likely involved in water transport in the plant body and leaves and in also root development (Additional file 2: Table S2). Most of the BrAQP proteins were highly similar to AQPs involved in water and solute transportation or fruit development in different plant species. Six, five and two of BrAQP proteins shared the highest degree of identity with proteins responsible for pod colour, tissue-specific expression and root development, respectively, in other plant species (Additional file 2: Table S2). Interestingly, the majority of BrPIP subfamily proteins showed high identity to abiotic stress-related AQP proteins from a wide range of plants (Additional file 2: Table S2). Therefore, we have selected BrPIP subfamily for details expression analysis. Out of 59 identified BrAQPs, 25 were most similar to abiotic stress (freezing, salt and drought)- and ABA-related AQP proteins in different plant species. Twenty out of those 25 belonged to the BrPIP subfamily are directly related to abiotic and ABA- stress responsive. Therefore, we concluded that PIP subfamily members among the BrAQP proteins are the most likely to be involved in water and solute transport in response to various abiotic stresses.

### Sequence analysis of *BrAQP* genes

Table 1 summarizes the aromatic/Arg (ar/R) selectivity filter (H2, H5, LE1 and LE2), Froger’s positions (P1 to P5), and the prediction of domains, subcellular localization, NPA motifs, and genome fractionation (subgenome) for the 59 AQP protein sequences. With the exception of BrPIP2;2b all of the predicted BrAQP proteins contained two conserved NPA motifs, in LB and LE. Each member of predicted BrSIP subgroup member contained unusual third amino acids in the motifs, with the alanine replaced by threonine, cysteine, leucine or valine. By contrast, *BrNIP1;2a, BrNIP1;2b, BrNIP6;1a* and *BrNIP6;1b* encoded motifs with a variable third residue in which alanine was replaced by glycine and valine. Meanwhile, *BrNIP5;1a* and *BrNIP5;1b* encoded dissimilar amino acids in both NPA motifs, where alanine was replaced with serine and valine, respectively. Based on our subcellular localization predictions, all members of the NIP, SIP and PIP subfamilies of *B. rapa* appear to be present in the cell membrane. However, members of TIP subfamily were predicted to be positioned on vacuoles, with BrTIP 5;1 located in both vacuole and cell membrane (Table 1).

**Table 1:** Subgenome position, conserved amino acid residues (NPA motif, Ar/R filter, Froger's position), the prediction of transmembrane and MIP domains and subcellular localization of *B. rapa* Aquaporins

Gene name	Sub genome	NPA motif		Ar/R selectivity filter				Froger’s Position (P1 - P5)					TMH + MIP	Subcellular localization
		LB	LE	H2	H5	LE1	LE2	P1	P2	P3	P4	P5		
*BrSIP1;1a*	LF	NPT	NPA	I	T	P	I	I	A	A	Y	W	6 + 1	CM
*BrSIP1;1b*	MF1	NPT	NPA	I	T	P	I	I	A	A	Y	W	6 + 1	CM
*BrSIP1;2*	LF	NPC	NPA	V	T	P	I	I	A	A	Y	W	6 + 1	CM
*BrSIP2;1a*	MF1	NPL	NPA	S	K	G	A	F	V	A	Y	W	6 + 1	CM
*BrSIP2;1b*	MF2	NPL	NPA	S	K	G	A	F	V	A	Y	W	6 + 1	CM
*BrSIP2;1c*	LF	NPV	NPA	S	K	G	A	F	V	A	Y	W	6 + 1	CM
*BrNIP1;2a*	LF	NPA	NPG	W	V	A	R	F	S	A	Y	I	6 + 1	CM
*BrNIP1;2b*	MF1	NPA	NPG	W	V	A	R	F	S	A	Y	I	6 + 1	CM
*BrNIP2;1a*	LF	NPA	NPA	W	V	A	R	F	S	A	Y	I	6 + 1	CM
*BrNIP2;1b*	LF	NPA	NPA	W	V	A	R	F	S	A	Y	I	6 + 1	CM
*BrNIP3;1a*	MF1	NPA	NPA	W	I	A	R	F	S	A	Y	I	6 + 1	CM
*BrNIP3;1b*	MF2	NPA	NPA	W	I	A	R	F	S	A	Y	I	6 + 1	CM
*BrNIP4;1*	MF2	NPA	NPA	W	V	A	R	F	S	A	Y	I	6 + 1	CM
*BrNIP4;2a*	LF	NPA	NPA	W	V	A	R	F	S	A	Y	I	6 + 1	CM
*BrNIP4;2b*	MF2	NPA	NPA	-	V	A	R	F	S	A	Y	I	4 + 1	CM
*BrNIP4;2c*	MF2	NPA	NPA	-	-	A	R	F	S	A	Y	I	3 + 1	CM
*BrNIP5;1a*	MF2	NPS	NPV	A	I	G	R	F	T	A	Y	L	6 + 1	CM
*BrNIP5;1b*	MF1	NPS	NPV	A	I	A	R	F	T	A	Y	L	6 + 1	CM
*BrNIP6;1a*	MF1	NPA	NPV	A	I	A	R	F	T	A	Y	L	6 + 1	CM
*BrNIP6;1b*	LF	NPA	NPV	A	I	A	R	F	T	A	Y	L	6 + 1	CM
*BrNIP7;1*	LF	NPS	NPA	A	V	G	R	Y	S	A	Y	M	6 + 1	CM
*BrTIP1;1*	MF1	NPA	NPA	H	I	A	V	T	A	A	Y	W	6 + 1	V
*BrTIP1;2a*	LF	NPA	NPA	H	I	A	V	T	A	A	Y	W	6 + 1	V
*BrTIP1;2b*	MF1	NPA	NPA	H	I	A	V	T	A	A	Y	W	6 + 1	V
*BrTIP1;3*	LF	NPA	NPA	H	I	A	V	T	S	A	Y	W	6 + 1	V
*BrTIP2;1a*	LF	NPA	NPA	H	I	G	R	T	S	A	Y	W	6 + 1	V
*BrTIP2;1b*	MF2	NPA	NPA	H	I	G	R	T	S	A	Y	W	6 + 1	V
*BrTIP2;1c*	MF1	NPA	NPA	H	I	G	R	T	S	A	Y	W	5 + 1	V
*BrTIP2;2*	LF	NPA	NPA	H	I	G	R	T	S	A	Y	W	6 + 1	V
*BrTIP2;3a*	LF	NPA	NPA	H	I	G	R	T	S	A	Y	W	6 + 1	V
*BrTIP2;3b*	MF1	NPA	NPA	H	I	G	R	T	S	A	Y	W	5 + 1	V
*BrTIP3;1a*	MF1	NPA	NPA	H	I	A	R	T	A	A	Y	W	6 + 1	V
*BrTIP3;1b*	LF	NPA	NPA	H	I	A	R	T	A	A	Y	W	6 + 1	V
*BrTIP3;2a*	LF	NPA	NPA	H	M	A	R	T	A	S	Y	W	6 + 1	V
*BrTIP3;2b*	MF1	NPA	NPA	H	M	A	R	T	A	S	Y	W	6 + 1	V
*BrTIP4;1*	MF1	NPA	NPA	H	I	A	R	T	S	A	Y	W	6 + 1	V
*BrTIP5;1*	LF	NPA	NPA	N	V	G	C	V	A	A	Y	W	6 + 1	V and CM
*BrPIP1;1a*	LF	NPA	NPA	F	H	T	R	Q	S	A	F	W	6 + 1	CM
*BrPIP1;1b*	MF1	NPA	NPA	F	H	T	R	Q	S	A	F	W	6 + 1	CM
*BrPIP1;2a*	MF1	NPA	NPA	F	H	T	R	Q	S	A	F	W	6 + 1	CM
*BrPIP1;2b*	LF	NPA	NPA	F	H	T	R	Q	S	A	F	W	6 + 1	CM
*BrPIP1;3a*	MF2	NPA	NPA	F	H	T	R	Q	S	A	F	W	6 + 1	CM
*BrPIP1;3b*	LF	NPA	NPA	F	H	T	R	Q	S	A	F	W	6 + 1	CM
*BrPIP1;4*	MF1	NPA	NPA	F	H	T	R	Q	S	A	F	W	6 + 1	CM
*BrPIP1;5*	MF1	NPA	NPA	F	H	T	R	Q	S	A	F	W	6 + 1	CM
*BrPIP2;1*	LF	NPA	NPA	F	H	T	R	Q	S	A	F	W	6 + 1	CM
*BrPIP2;2a*	MF2	NPA	NPA	F	H	T	R	Q	S	A	F	W	6 + 1	CM
*BrPIP2;2b*	LF	NPA	-	F	-	-	-	Q	-	-	F	W	5 + 1	CM
*BrPIP2;3a*	MF2	NPA	NPA	F	H	T	R	Q	S	A	F	W	6 + 1	CM
*BrPIP2;3b*	LF	NPA	NPA	F	H	T	R	Q	S	A	F	W	5 + 1	CM
*BrPIP2;4a*	MF2	NPA	NPA	F	H	T	R	Q	S	A	F	W	6 + 1	CM
*BrPIP2;4b*	MF1	NPA	NPA	F	H	T	R	Q	S	A	F	W	6 + 1	CM
*BrPIP2;4c*	LF	NPA	NPA	F	H	T	R	Q	S	A	F	W	6 + 1	CM
*BrPIP2;5a*	LF	NPA	NPA	F	H	T	R	Q	S	A	F	W	6 + 1	CM
*BrPIP2;5b*	MF2	NPA	NPA	F	H	T	R	Q	S	A	F	W	6 + 1	CM
*BrPIP2;6*	MF2	NPA	NPA	F	H	T	R	Q	S	A	F	W	6 + 1	CM
*BrPIP2;7a*	MF2	NPA	NPA	F	H	T	R	M	S	A	F	W	6 + 1	CM
*BrPIP2;7b*	LF	NPA	NPA	F	H	T	R	M	S	A	F	W	6 + 1	CM
*BrPIP2;7c*	MF1	NPA	NPA	F	H	T	R	M	S	A	F	W	6 + 1	CM

Blue colour letters denote unusual amino acids in NPA motifs. *CM* Cell membrane, *V*Vacuole

*LF* Less Fractioned subgenome, *MFs (MF1 and MF2)* More Fractioned subgenomes, *LB* Loop B, *LE* Loop E, {two half helices (LB and LE)}, *NPA* Asparagine, Proline, Alanine, *AQP* contain 6 TM helices (H1 to H6), *H2* Helice 2, *H5* Helice 5, *LE1*Loop E1, *LE2* Loop E2, *Ar/R* Aromatic/Arginine, *TMH* Transmembrane helice

The ar/R selectivity filter and five Froger’s positions of the BrNIP subfamily members were quite divergent compared to those of the other subfamilies (Table 1 and Additional file 3: Figure S1a ~ 1d). The predicted polypeptides of the SIP subfamily were divided into two groups (SIP1 and SIP2) and showed 22.6–91.1% identity within the subfamily, but 72.1–91.1% identity within the groups. The ar/R filter and five Froger’s positions P1 to P5 of the SIP subfamily were well conserved in all sites. The 16 putative TIP subfamily members were divided into 5 groups and showed 68.2–94.8% identity within groups (Additional file 4: Table S3).

### Phylogenetic analysis of BrAQP proteins

**Fig. 1:**
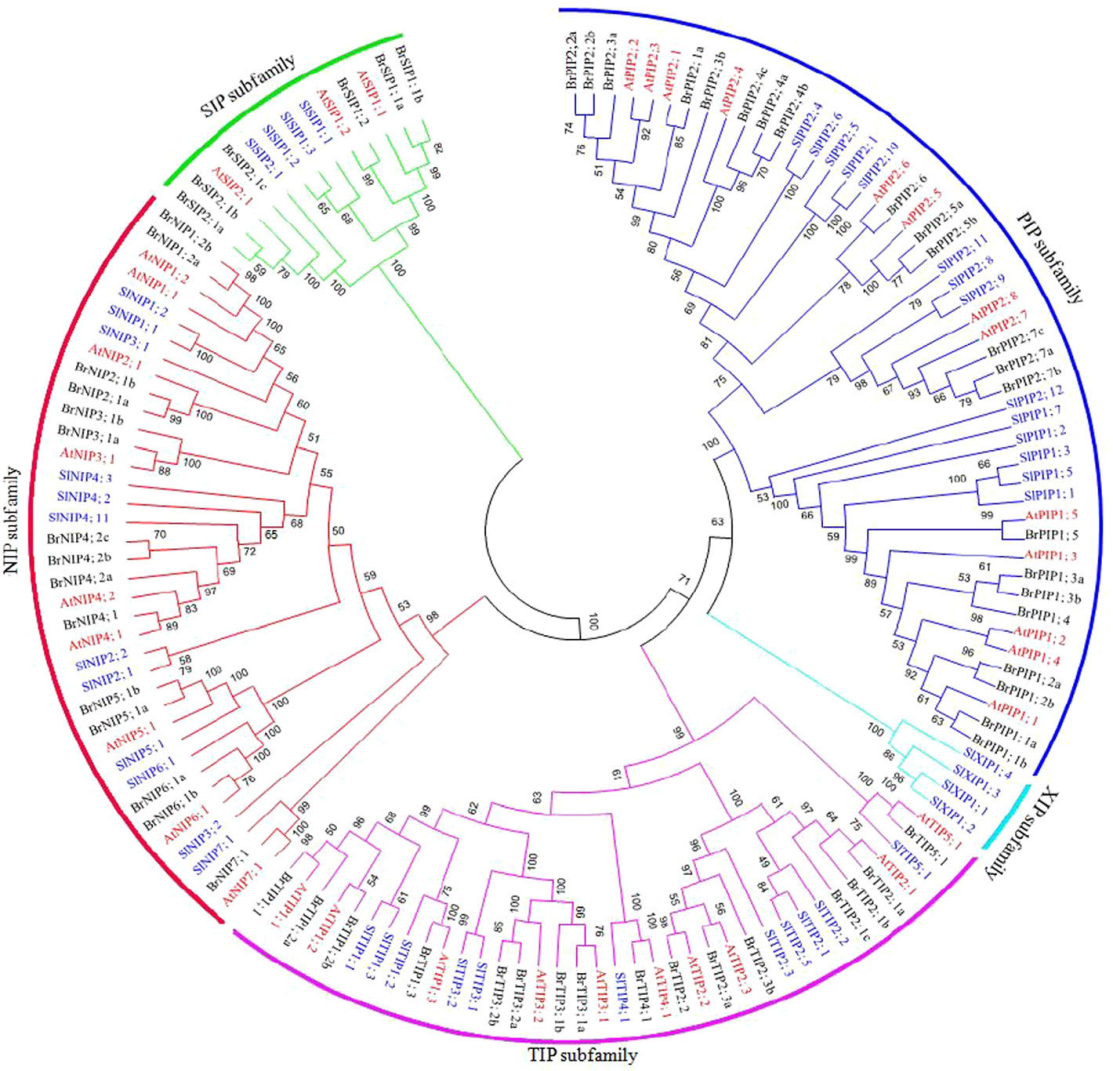
Phylogenetic analysis of aquaporin proteins identified in *B. rapa,* Arabidopsis and tomato. Based on relatedness to characterized proteins, aquaporins were classified as plasma membrane intrinsic proteins (PIPs) in the *blue tree*, tonoplast intrinsic proteins (TIPs) in the pink tree, nodulin 26-like intrinsic proteins (NIPs) in the red tree, small basic intrinsic proteins (SIPs) in the green tree and X intrinsic proteins (XIP) in the deep green tree*. At, Sl, Br* denote *Arabidopsis, tomato and B. rapa* and *red*, *blue* and *black* letters denote *Arabidopsis,* tomato *and B. rapa* aquaporin proteins, respectively

The phylogenic tree was constructed based on the multiple sequence alignment of 59, 45 and 35 putative full-length BrAQP, SiAQP and AtAQP proteins, respectively (Fig. 1). The BrAQPs were classified into four subfamilies (PIP, TIP, NIP and SIP) corresponding to the *Arabidopsis* grouping defined by Quigley et al. [10]. The six newly identified *B. rapa* genes were distributed in PIP, NIP and TIP subfamilies, with each subfamily containing 2 members. Accordingly, these new members are named as BrNIP4;2b, BrNIP4;2c, BrPIP2;2b, BrPIP2;3b, BrTIP2;1c and BrPIP2;3b. Among the subfamilies, PIP had the most BrAQPs and contained 22 members, relative to the 16, 15 and 6 members of the TIP, NIP and SIP subfamilies, respectively. Members of XIP subfamily were totally absent in *B. rapa* (Fig. 1).

### Chromosomal locations and gene duplications of *BrAQP* genes

**Fig. 2:**
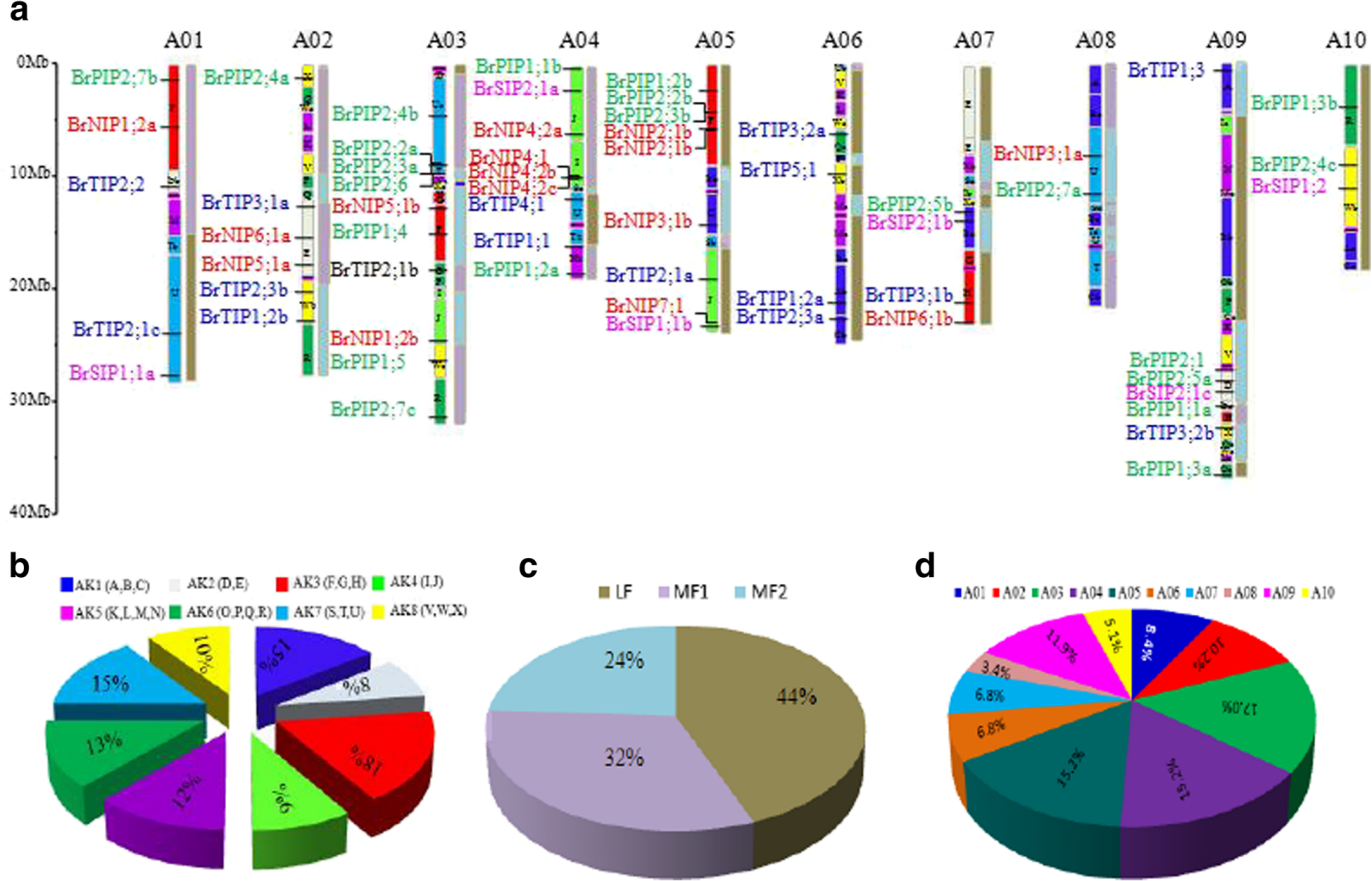
Distribution of *BrAQP* genes on 10 chromosomes. **a** The 24 (A to X) ancestral blocks and three sub-genomes were plotted, based on the report of Schranz et al. [25]. **b** The percentages of *BrAQP* genes on ancestral blocks. **c** The percentages of *BrAQP* genes on the least fractionated (LF), medium fractionated (MF1) and most fractionated (MF2) subgenomes. **d** The percentages of BrAQP genes on each chromosome

We conducted *in silico* analysis to determine the localization of *AQP* genes in 10 chromosomes of *B. rapa* using gene mapping software (Fig. 2a). The most *AQP* genes were found in chromosome 3 (17.0%) and the fewest were found in chromosome 8 (3.4%) (Fig. 2d). The physical locations of the *BrAQP* genes in the *B. rapa* genome reflected the diversity and complexity of this gene family. The PIP subfamily genes were distributed on all chromosomes except chromosome 6, and TIP subfamily genes were found in all chromosomes except chromosomes 8 and 10. Other than chromosomes 6, 9 and 10, there were NIP group genes in each chromosome. Genes in the SIP subfamily were present only on chromosomes 1, 4, 5, 7, 9 and 10 (Fig. 2a). Genome triplication has occurred since divergence of the *Brassica* genus from the ancestor of *A. thaliana* between five and nine million years ago (MYA) [24]. The *B. rapa* genome consists of three differentially fractionated sub-genomes, namely the least fractionated (LF), medium fractionated (MF1), and most fractionated (MF2). The 59 *BrAQPs* were fractionated into three subgenomes (i.e., LF, MF1, and MF2), including 26 (44%) in LF, 19 (32%) in MF1, and 14 (24%) in MF2 (Fig. 2c and Table 1). In addition, we reconstructed the *B. rapa* genome containing 24 conserved chromosomal blocks (labelled A–X) according to previous reports [25]. The colour coding of these blocks was based on their positions in a proposed ancestral karyotype (AK1-8) [25]. Most of the 59 *BrAQP* genes belonged to AK3 (18%), followed by AK1 and AK7 (15%), while only 8% of *BrAQP* genes were assigned to AK2 (Fig. 2b).

**Fig. 3:**
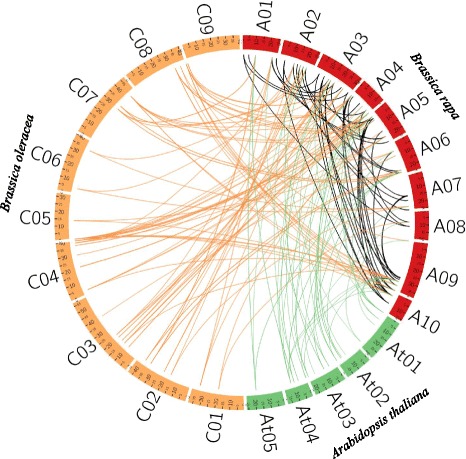
Microsynteny analysis of *AQP* genes among *B. rapa, B. oleracea* and *A. thaliana*. The chromosomes from the three species are indicated in different colors; red, green and yellow colors represent *B. rapa, A. thaliana* and *B. oleracea* chromosomes, respectively. Black lines denote duplicated *BrAQP* genes on 10 *B. rapa* chromosomes

The arrangement of *BrAQP* genes in the *B. rapa* genome implies that some genetic events have affected this gene family during evolution. The distribution of the *AQP* gene family has likely been influenced by processes such as segmental duplication, tandem duplication, and polyploidization [26, 27]. In addition, genome triplication events might have played a key role in the expansion of *AQP* gene family in *B. rapa.* We found evidence of at least two tandem duplication events (*BrNIP4;1* vs. *BrNIP4;2b*, *BrNIP4;2b* vs. *BrNIP4;2c*) with total of 43 segmental duplications in the *BrAQP* gene family (Table 2, Fig. 3). Estimation of the Ka/Ks ratios (synonymous and nonsynonymous substitutions per site) was done to assess the selection constraints among duplicated *BrAQP* gene pairs. In these analyses, Ka/Ks ratios <1, 1 and >1 indicate negative or purifying selection, neutral selection and positive selection, respectively [28]. All *BrAQP* duplicated gene pairs showed a Ka/Ks ratio of <1, suggesting that these genes evolved under strong negative or purifying selection pressure in *B. rapa*. These results suggest that purifying selection has played an important role in the functional divergence of *BrAQP* genes. We calculated the divergence time of *BrAQP* genes and found that these gene duplications began approximately 9.39 million year (mya) ago and ended at 0.38 mya ago (Table 2), which indicates that the divergence time of the *AQP* genes in *B. rapa* occurred after the triplication events (i.e., 5 ~ 9 MYA) [29].

**Table 2:** Estimated Ka/Ks ratios of the duplicated *BrAQP* genes with their divergence time in *B. rapa*

Duplicated gene pairs			Ks	Ka	Ka/Ks	Duplication type	Purify selection	Time (mya)
BrSIP1;1b (MF1)	vs.	BrSIP1;1a (LF)	0.191	0.034	0.18	Segmental	Yes	0.64
BrSIP2;1b (MF2)	vs.	BrSIP2;1a (MF1)	0.241	0.059	0.24	Segmental	Yes	0.80
BrSIP2;1b (MF2)	vs.	BrSIP2;1c (LF)	0.307	0.084	0.27	Segmental	Yes	1.02
BrNIP1;2b (MF1)	vs.	BrNIP1;2a (LF)	0.314	0.006	0.02	Segmental	Yes	1.05
BrNIP3;1a (MF1)	vs.	BrNIP3;1b (MF2)	0.421	0.051	0.12	Segmental	Yes	1.40
BrNIP4;1 (MF2)	vs.	BrNIP4;2b (MF2)	0.376	0.068	0.18	Tandem	Yes	1.25
BrNIP4;2b (MF2)	vs.	BrNIP4;2c (MF2)	0.338	0.077	0.23	Tandem	Yes	1.13
BrNIP5;1b (MF1)	vs.	BrNIP5;1a (MF2)	0.282	0.006	0.02	Segmental	Yes	0.94
BrNIP6;1b (LF)	vs.	BrNIP6;1a (MF1)	0.283	0.034	0.12	Segmental	Yes	0.94
BrPIP1;1a (LF)	vs.	BrPIP1;1b (MF1)	0.229	0.012	0.05	Segmental	Yes	0.76
BrPIP1;1a (LF)	vs.	BrPIP1;2b (LF)	0.727	0.018	0.02	Segmental	Yes	2.42
BrPIP1;1a (LF)	vs.	BrPIP1;3b (LF)	0.925	0.091	0.10	Segmental	Yes	3.08
BrPIP1;1b (MF1)	vs.	BrPIP1;3b (LF)	0.867	0.085	0.10	Segmental	Yes	2.89
BrPIP1;1b (MF1)	vs.	BrPIP1;2b (LF)	0.768	0.012	0.02	Segmental	Yes	2.56
BrPIP1;2b (LF)	vs.	BrPIP1;2a (MF1)	0.224	0.027	0.12	Segmental	Yes	0.75
BrPIP1;2b (LF)	vs.	BrPIP1;3a (MF2)	1.013	0.054	0.05	Segmental	Yes	3.38
BrPIP1;2b (LF)	vs.	BrPIP1;3b (LF)	0.948	0.066	0.07	Segmental	Yes	3.16
BrPIP1;3a (MF2)	vs.	BrPIP1;4 (MF1)	0.672	0.041	0.06	Segmental	Yes	2.24
BrPIP1;3b (LF)	vs.	BrPIP1;3a (MF2)	0.114	0.017	0.15	Segmental	Yes	0.38
BrPIP1;3b (LF)	vs.	BrPIP1;4 (MF1)	0.705	0.047	0.07	Segmental	Yes	2.35
BrPIP2;1 (LF)	vs.	BrPIP2;2b (LF)	0.693	0.152	0.22	Segmental	Yes	2.31
BrPIP2;1 (LF)	vs.	BrPIP2;2a (MF2)	0.786	0.160	0.20	Segmental	Yes	2.62
BrPIP2;2b (LF)	vs.	BrPIP2;2a (MF2)	0.377	0.026	0.07	Segmental	Yes	1.26
BrPIP2;3a (MF2)	vs.	BrPIP2;3b (LF)	0.410	0.023	0.06	Segmental	Yes	1.37
BrPIP2;3a (MF2)	vs.	BrPIP2;5a (LF)	1.160	0.195	0.17	Segmental	Yes	3.87
BrPIP2;3a (MF2)	vs.	BrPIP2;4c (LF)	2.817	0.117	0.04	Segmental	Yes	9.39
BrPIP2;3b (LF)	vs.	BrPIP2;2a (MF2)	0.351	0.006	0.02	Segmental	Yes	1.17
BrPIP2;3b (LF)	vs.	BrPIP2;1 (LF)	0.734	0.026	0.04	Segmental	Yes	2.45
BrPIP2;3b (LF)	vs.	BrPIP2;4c (LF)	1.325	0.091	0.07	Segmental	Yes	4.42
BrPIP2;4a (MF2)	vs.	BrPIP2;4b (MF1)	0.106	0.020	0.19	Segmental	Yes	0.35
BrPIP2;4a (MF2)	vs.	BrPIP2;4c (LF)	0.172	0.012	0.07	Segmental	Yes	0.57
BrPIP2;4b (MF1)	vs.	BrPIP2;4c (LF)	0.142	0.020	0.14	Segmental	Yes	0.47
BrPIP2;5a (LF)	vs.	BrPIP2;5b (MF2)	0.374	0.028	0.07	Segmental	Yes	1.25
BrPIP2;7a (MF2)	vs.	BrPIP2;7c (MF1)	0.176	0.035	0.20	Segmental	Yes	0.59
BrPIP2;7a (MF2)	vs.	BrPIP2;7b (LF)	0.415	0.032	0.08	Segmental	Yes	1.38
BrPIP2;7b (LF)	vs.	BrPIP2;7c (MF1)	0.304	0.011	0.04	Segmental	Yes	1.01
BrTIP1;1 (MF1)	vs.	BrTIP1;2b (MF1)	0.590	0.093	0.16	Segmental	Yes	1.97
BrTIP1;1 (MF1)	vs.	BrTIP1;2a (LF)	0.590	0.061	0.10	Segmental	Yes	1.97
BrTIP1;2a (LF)	vs.	BrTIP1;2b (MF1)	0.964	0.167	0.17	Segmental	Yes	3.21
BrTIP2;1a (LF)	vs.	BrTIP2;1b (MF2)	0.133	0.035	0.26	Segmental	Yes	0.44
BrTIP2;1a (LF)	vs.	BrTIP2;1c (MF1)	0.170	0.029	0.17	Segmental	Yes	0.57
BrTIP2;1b (MF2)	vs.	BrTIP2;1c (MF1)	0.134	0.017	0.13	Segmental	Yes	0.45
BrTIP2;3a (LF)	vs.	BrTIP2;3b (MF1)	0.514	0.041	0.08	Segmental	Yes	1.71
BrTIP3;1a (MF1)	vs.	BrTIP3;1b (LF)	0.337	0.051	0.15	Segmental	Yes	1.12
BrTIP3;2a (LF)	vs.	BrTIP3;2b (MF1)	0.354	0.017	0.05	Segmental	Yes	1.18

*LF* less fractioned subgenome, *MF* more fractioned subgenome (MF1 and MF2), *Ks* the number of synonymous substitutions per synonymous site, *Ka* the number of nonsynonymous substitutions per nonsynonymous site, *MYA* million years ago

### Microsynteny relationships

To investigate evolutionary history and relationships, a microsynteny map was constructed using orthologous gene pairs of the *AQP* genes among *B. rapa*, *B. oleracea* and *A. thaliana* (Fig. 3). Based on this analysis, 39 orthologous gene pairs between *B. rapa* and *A. thaliana* were identified, whereas 72 orthologous gene pairs were found between *B. rapa* and *B. oleracea* (Fig. 3). This result suggests that *BrAQP* genes are more closely related to those of *B. oleracea* and *A. thaliana.* We found 45 duplications of *BrAQP* genes. Out of 45 pairs, 43 were segmental and 2 pairs were identified as tandem duplications, which is denoted with a black line in Fig. 3. For clarity, we have also depicted only the *BrAQP* duplicated gene pairs in *B. rapa* chromosomes (Additional file 5: Figure S2).

### Motif and exon-intron distribution

Conserved motifs among each subfamily were identified using MEME software and compared for providing further support of the grouping of *BrAQPs*. Most BrAQP proteins of the same subfamily had similar motifs, with motifs 1 & 2 present in all subfamilies (Additional file 6: Figure S3). The protein sequences of all *BrAQPs* shared high similarity; thus, out of the 10 motifs, most (1, 2, 3, 4, 5, 6, 7 and 9) were found in all PIP subfamily members except *BrPIP2;3b* and *BrPIP2;4c*, which were lacked of motif 5, and *BrPIP1;2a,* which had no motif 4 (Additional file 6: Figure S3). Motifs 1, 2, 3, 6 and 10 were common to both TIP and NIP subfamily members, although *BrTIP2;1c, BrTIP2;3b, BrNIP4;2b,* and *BrNIP4;2c* did not contain motif 10. A unique motif (motif 8) was found in TIP group members, and motif 6 was found only in subfamily SIP1. The best possible match sequence for each motif is presented in Additional file 7: Table S4.

The intron–exon structures of the *B. rapa AQPs* were analyzed using the GSDS program. Most members of the PIP subfamily had three introns, while four members had two introns and two members had four introns. In the TIP subfamily, eight members had two introns and seven members had one intron, but only one gene had no intron. All BrNIP family members had 2 to 4 introns; 7 out of 15 members had 3 introns, another 7 members had 4 introns, and only 1 had 2 introns. BrSIPs formed a small subfamily of *BrAQP* in which all members had two introns (Additional file 8: Figure S4).

### Microarray expression analysis in response to cold and freezing stress

**Fig. 4:**
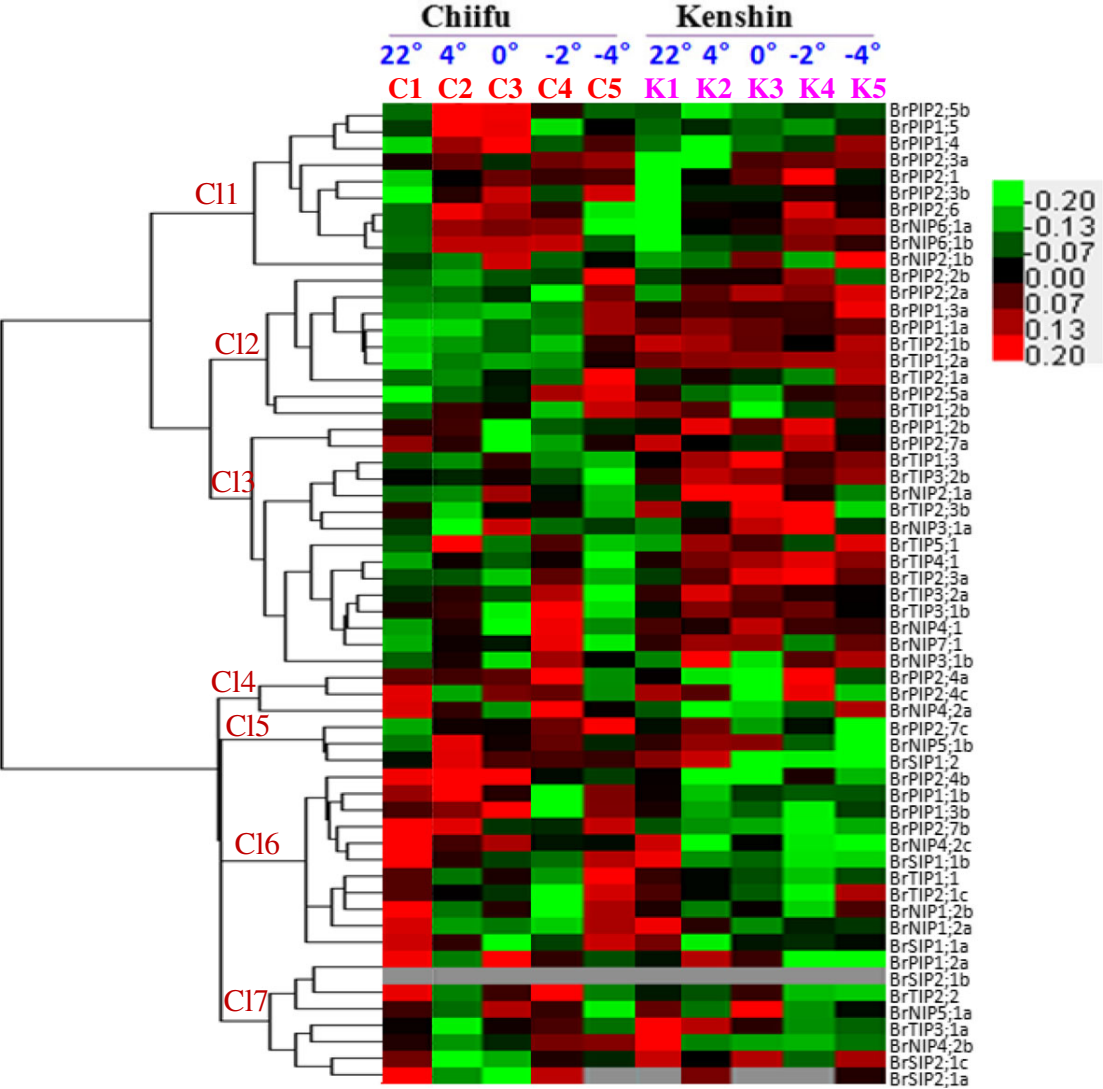
Differential expression profiles of *BrAQP* genes in different temperatures. C and K indicate Chiifu and Kenshin, respectively, which were treated under five temperatures: control (C1&K1), 4 °C (C2 & K2), 0 °C (C3 & K3), −2 °C (C4 & K4), and **-** 4 °C (C5 & K5). Expression clusters are shown in the left (Cl1–Cl7) and gene names are at the right. Color legend at right represents differential expression in microarray data

Expression patterns of the 59 *BrAQP* genes were determined using our previously published microarray data set, wherein two contrasting *B. rapa* inbred lines cold-tolerance Chiifu and cold susceptible Kenshin, were treated with different temperatures (22 °C, 4 °C, 0 °C, −2 °C and −4 °C) [30]. The two lines (Chiifu and Kenshin) responded differently in microarray expression. Chiifu originated in temperate regions, whereas Kenshin originated in tropical and subtropical regions. At low temperature, Kenshin shows severe injury while Chiifu does not [31]. Moreover, Kenshin has been used as a breeding stock to develop heat-tolerant plants [32]. We created a heat map based on differential microarray transcript values and to examine expression pattern of *BrAQP* genes in response to temperature treatments in two inbred lines (chiifu and kenshin) of *B. rapa* (Fig. 4). In the heat map, expression patterns of *BrAQP* genes were divided into seven clusters (Cl-1 to Cl-7). Most *BrPIP* genes were present in Cl-1, Cl-2, Cl-4 and Cl-6. The *BrPIP* genes in Cl-1, Cl-4 and Cl-6 showed higher expression in Chiifu than in Kenshin in response to both cold and freezing temperatures. Five *BrPIP* genes in Cl-2 showed higher expression in Kenshin than in Chiifu under normal conditions (22 °C). However, Cl-2 and Cl-3 *BrAQP* genes exhibited higher expression in Kenshin than in Chiifu in response to both cold and freezing temperatures. *BrSIP2;1b* did not show a significant response in any temperature treatment, whereas *BrSIP2;1a* did not respond to freezing temperatures. As a whole, we concluded that the majority of *BrPIP* subfamily genes were highly induced in Chiifu by cold and freezing treatment compared to in Kenshin. These results indicate that *BrPIP* subfamily genes might play an important role in the cold and freezing tolerance of Chiifu. On the contrary, a few *BrPIP* and those of other *BrAQP* subfamilies showed higher expression in Kenshin in response to cold and freezing temperature; those genes might be related to the cold and freezing susceptibility of Kenshin.

### Expression profiles of BrPIP genes in various organs

**Fig. 5:**
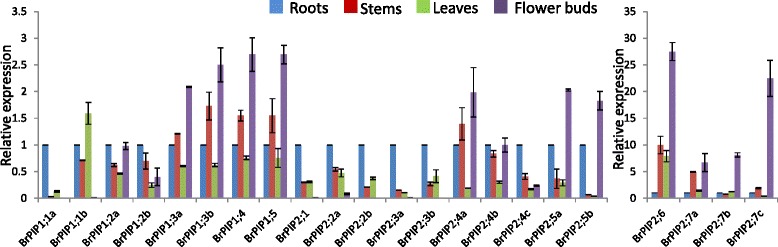
Expression profiles of *BrPIP* genes in various tissues as determined by qPCR analyses. Expression of the indicated genes was determined in roots, stems, leaves, and flower buds

The expression of 22 *PIP* genes in different organs of *B. rapa* plants (roots, stems, leaves, and flower bud) was analyzed by qPCR and semi-quantitative RT-PCR (Fig. 5, Additional file 9: Figure S5). Eighteen PIP genes (*BrPIP1;1b, 1;2a, 1;2b, 1;3a, 1;4, 1;5, 2;1, 2;2a, 2;3a, 2;4a; 2;4b; 2;4c, 2;5a; 2;5b, 2;6, 2;7a 2;7b,* and *2;7c*) were expressed in all tested organs but *BrPIP1;1b* and *2;3a* were only slightly expressed in flower buds. Two genes (*2;2b and 2;3b*) were abundant in all of the tested organs except flower bud. *BrPIP1;1a* was highly expressed in roots and leaves and slightly expressed in stem but absent in flower buds. By contrast, *BrPIP2;5a,* and *BrPIP2;5b* were highly expressed in roots and flower buds but slightly expressed in stems and leaves. *BrPIP1;3a, 1;3b, 1;4, 1;5, 2;4a, 2;5a, 2;5b, 2;6, 2;7a, 2;7b, and 2;7c* were highly expressed in flower buds compared to other organs. However, *BrPIP1;1a, 1;2b, 2;1, 2;2a, 2;2b, 2;3a, 2;3b* and *2;4c* were more abundantly expressed in roots compared to other tested parts (Fig. 5). In most of the cases, qPCR and RT-PCR results were consistent, although slightly different results were found for *BrPIP2;4a, 2;4b, 2;5a, 2;5b* and *2;6* (Fig. 5, Additional file 9: Figure S5).

### Stress-responsive expression analysis

**Fig. 6:**
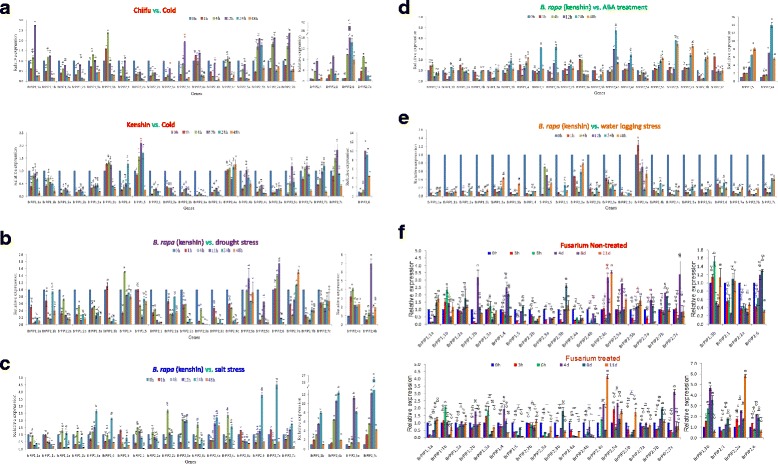
Expression analysis of *BrPIP* genes under abiotic stresses using real-time quantitative RT-PCR. The relative expression levels of *BrPIP* genes under treatment with (**a**) cold, (**b**) drought, (**c**) salinity (**d**) ABA (**e**) waterlogging or (**f**) *Fusarium oxysporum* f.sp. *conglutinans* infection. The error bars represent the standard error of the means of three independent replicates. Variance analysis and the Tukey tests were carried out to determine differences among effects on different time courses due to abiotic and biotic stresses for all genes, where different letters indicate the significant difference with *p* ≤ 0.05

Crop loss due to abiotic stresses decrease average yields of most important crops and threatens food security worldwide [33]. Therefore, identification of stress-responsive genes is an important basic step towards developing stress tolerant cultivars. Accordingly, we analyzed the expression of *BrPIP* subfamily genes for responsiveness to cold, drought, salt, water logging and ABA in *B. rapa* plants via qPCR using specific primers (Additional file 10: Table S5). As in the analysis of microarray data described above, two inbred lines of *B. rapa*, Chiifu and Kenshin, were used to detect the responses of *BrPIP* genes expression due to cold stress. All of the *BrPIP* genes showed higher expression in Chiifu compared to Kenshin except *BrPIP2;4b*, which did not show any higher expression change due to cold treatment either in Chiifu or in Kenshin compared to the control (Fig. 6a). Out of 22 *BrPIP* genes, 14 were differentially expressed in response to cold stress at different time points. The majorities of the genes were down-regulated at the beginning of the cold treatment, but began to be up-regulated after 4 h and continue to increase in expression up to 12 h of time course. Thereafter, the same genes were down-regulated until the end of the time courses (Fig. 6a). In Chiifu, *BrPIP1;1a, BrPIP1;4, BrPIP1;5* and *BrPIP2;6* genes showed about 3-, 8-, 10- and 41- fold higher expression at 12 h, respectively, and *BrPIP2;7c* showed about 10-fold higher expression at the 4 h time point compared to the 0 h time point. The fold changes of the expression of those genes were significantly (p ≤ 0.01) different from each other at the mentioned time points (Fig. 6a). By contrast, the majority of *PIP* genes showed down-regulation in Kenshin upon cold treatment. Only a few *PIP* genes such as *BrPIP1;3b, 1;5, 2;5b; 2;7a* and *2;7b* showed differential expression in response to cold stress in Kenshin, and their expression levels were very low. In Kenshin, *BrPIP2;6* and *BrPIP2;7c* exhibited about 10- and 2-fold higher expression at the 12 h time point compared to the control and their expression subsequently started to decreases however the expression differences between those two genes were statistically significant (p ≤ 0.01; Fig. 6a).

We next used Kenshin for qRT-PCR assays to elucidate the responses of *BrPIP* genes to drought stress. Differential expression of *BrPIP1;4, 2;4a, 2;4b, 2;5a, 2;6* and *2;7a* were observed during drought and the differences of the expression were significant (p ≤ 0.01) among the genes (Fig. 6b). *BrPIP2;4b, 2;5a* and *2;6* showed up-regulation up to 12 h, but *BrPIP1;4,* and *2;4a,* showed up-regulation up to 4 h and were subsequently down-regulated to the end of the time courses (Fig. 6b). Meanwhile, *BrPIP2;7a* showed down-regulation at the initial stage of stress and was gradually up-regulated thereafter, whereas *BrPIP1;3b* showed up-regulation at the beginning of drought (1 h) but was subsequently down-regulated. The rest of the *BrPIP* genes were down-regulated soon after drought stress and remained consistent throughout the stress period. These results are in agreement with those for plasma membrane *AQPs* in response to abiotic stresses in *Arabidopsis thaliana* [17].

The majority of the *BrPIP* genes were significantly up-regulated during salt-stress (p ≤ 0.01). *BrPIP1;3a, 1;3b, 2;4a, 2;4b, 2;7b* and *2;7c* were up-regulated and showed the highest expression at 24 h and then were down-regulated. *BrPIP1;2a, 1;2b, 1;4, 1;5*, *2;3b* and *2;4c* were alternately up- and down-regulated throughout the treatment time course (Fig. 6c). Under salt stress, *BrPIP2;1, 2;2a* and *2;2b* showed down-regulation at 1 h but exhibited higher expression at 4 h; thereafter they were gradually down-regulated up to the end of time courses. By contrast, *BrPIP2;3a* expression reached a peak at 4 h and remain unchanged up to 24 h, followed by a radical down-regulation at 48 h. *BrPIP2;5a* showed slight down-regulation at 1 h followed by up-regulation (up to 12 fold compared to the control) at 12 h, but again started down-regulation to the end of the time course (Fig. 6c). *BrPIP2;6* and *2;7a* were down-regulated at the beginning of salt stress and continues to 12 h; thereafter they suddenly exhibited higher expression at 24 h. During salt stress, *BrPIP1;3b, 2;4b, 2;6, 2;7a* and *2;7c* showed about 8-, 14-, 4-, 5- and 26- fold higher expression compared to the control at 24 h, respectively, while *2;5a* showed 12- fold higher expression at 12 h and those expression fold changes were statistically significant (p ≤ 0.01; Fig. 6c). The *BrPIP* gene expression under salt stress treatment was similar to that of plasma membrane *AQPs* in *A. thaliana* under abiotic stresses [17].

Abscisic acid (ABA) is an important phytohormone that plays a vital role in plant growth and development as well as in responses to a wide range of stresses. As shown in Fig. 6d, most of the *BrPIP* genes were up-regulated in response to ABA treatment and showed their highest expression at 24 h. A small number of *BrPIP* genes (*BrPIP1;1a,* and *BrPIP2;3a*) exhibited higher expression at 4 h, while *BrPIP1;2a* and *BrPIP2;7c* peaked at 1 h and decreased thereafter. *BrPIP2;1, BrPIP2;2a, BrPIP2;4b, BrPIP2;6,* and *BrPIP2;4a* genes showed the highest expression at the 24 h time point*.* By contrast, *BrPIP1;2b, BrPIP2;3b* and *BrPIP2;5a* were down-regulated throughout the ABA treatment. *BrPIP1;5* exhibited about 8- fold higher expression at 48 h and *BrPIP 2;4a* showed about 14- fold higher expression at 24 h; the expression change of those genes was statistically significant (p ≤ 0.01) compared to other genes in the same time courses (Fig. 6d).

In the case of water logging stress, all *BrPIP* genes except *BrPIP2;4a* exhibited down-regulation compared to control. Some *BrPIP* genes showed increasing expression from 12 h to the end of treatment, but their relative expression remained below that of the control (Fig. 6e).

### Expression of *BrPIP* genes under biotic stress

We also analyzed the responses of *BrPIP* genes to biotic stress treatment using *Fusarium oxysporum* f.sp*. conglutinans*, which specifically attacks *Brassica* species and causes wilt diseases. Upon artificial infection by this pathogen, 4 out of the 22 *BrPIP* genes showed significantly higher expression (p ≤ 0.01; Fig. 6f). *BrPIP1;3b, BrPIP2;6* and *BrPIP2;1* displayed about 4.5-, 2- and 1.5- fold higher expression at 4 dai (days after infection), respectively. *BrPIP2;2a* exhibited about 6- fold higher expression at 11 dai compared to mock-treated plants (Fig. 6f). These results suggest that *BrPIP1;3b, BrPIP2;6, BrPIP2;1* and *BrPIP2;2a* may be involved in responses to *F. oxysporum* f.sp*. conglutinans* infection.

## Discussion

*AQP* genes are ubiquitously important in higher plants because of their function as water and/or small neutral solute transporters in plant body. Precise gene annotation is an important starting point for future functional studies of this family. The *AQP* gene family has 35 members in *Arabidopsis* and 47 members in tomato [8]. Meanwhile, we have found 59 *AQPs* in *B. rapa* and carried out *in silico* functional analysis, which showed that most of the PIP subfamily proteins shared a high degree of identity with abiotic stress-related AQP proteins from other plant species. Proteins of another three subfamilies (SIP, NIP and TIP) exhibited similarity to *AQPs* in crop plants involved in water and solute transport in leaves and fruits during fruit development, pod development, root development, nutrient uptake and arsenic transportation. All of the members of PIP, NIP and SIP subfamily and most of the TIP subfamily members contained the same ar/R selectivity filter and Froger’s positions. In some cases, these were different in TIP subfamily which is consistent with previous research [34]. The ar/R selectivity filter and Froger’s positions in the BrTIP subfamily members were quite divergent compared to those of the other subfamilies, indicating that they have different solute permeability.

Nineteen members of the *BrPIP* subfamily showed high similarity to both water flow and abiotic stress-related *PIP* genes from other plant species, whereas three showed high similarity to proteins involved in water flow between the pollen and stigma papillae, and abiotic stress-related *PIP* genes from other plant species (Additional file 2: Table S2). We therefore concluded that AQPs of *B. rapa* are likely involved in water and solute transport and that *BrPIP* subfamily members might be involved in abiotic stress responses as well. We analyzed the relative expression patterns of 59 *BrAQP* genes using a whole-genome microarray dataset obtained upon treatment at various temperatures (22, 4, 0, −2, and −4 ° C) in two inbred lines of *B. rapa*; Chiifu and Kenshin [31]. Thereafter, *BrPIP* subfamily genes were selected based on their variation in transcript abundance compared to the control, and analyzed for responsiveness to temperature treatments in those two contrasting *B. rapa* inbred lines (Fig. 4). The results indicated that *BrPIP* genes might play a vital role in abiotic stress responses in *B. rapa.* On the other hand, the *BrPIP* subfamily members were highly conserved, indicating their probable involvement in similar biological functions.

From an evolutionary viewpoint, gene number increases can be due to gene duplication events, including tandem and segmental duplication [35]. Gene duplication may play the driving role in the evolution of gene families and genetic systems [36]. Here, we identified 43 segmental duplicated gene pairs and two pairs tandemly duplicated genes (Table 2), suggesting that segmental duplication was the main contributor to the expansion of this gene family. We analyzed the evolutionary history of this family and calculated the Ka, Ks and Ka/Ks ratios of duplicated gene pairs. Interestingly, all gene pairs had Ka/Ks ratios <1 (Table 2), indicating that the *BrAQP* gene family has undergone large-scale purifying selection. The evolutionary timescale of *B. rapa* was estimated based on the synonymous substitution rate [37], revealing that the divergence time of the duplicated *BrAQP* genes spanned 0.38 to 9.39 million years, which suggests that duplication-based divergence of the *BrAQP* family members in *B. rapa* occurred after the triplication events (i.e., 5 ~ 9 MYA) [27]. Our microsynteny analysis showed that there are 39 and 72 orthologous gene pairs between *B. rapa* / *A. thaliana* and *B. rapa* / *B. oleracea*, respectively (Fig. 2).

Based on our organ-specific expression analysis, all *BrPIP* genes are expressed at different levels in at least one of the tested organs of *B. rapa* plants. *BrPIP1;1a, 1;2a; 2;2a,* and *2;3a* were more abundantly expressed in roots compared to other tested organs; which is consistent with previous findings [4, 17, 20]. *BrPIP1;2b, 1;3a, 1;4, 2;2a,* and *2;3a* were abundantly expressed in stem while *BrPIP1;1a, 1;2b* and *2;2a* were highly expressed in leaves, like their *Arabidopsis* counterparts. Previous reports have been suggested that *AQP* genes are expressed in all plant tissues and are involved in growth and development and responses to environmental stress conditions [5]. This abundantly expressed *BrPIP* genes in roots, stem and leaves might be related to different cellular controls of water flow. However, *BrPIP1;2a, 1;2b, 1;3a, 1;4, 2;5b 2;6; 2;7a, 2;7b* and *2;7c* were typically more expressed in flower buds of *B. rapa* plants (Fig. 4). Pollen absorbs water from the stigma surface before it germinates [38]. According to Marin-Olivier et al. [39] water flows from stigma papillae to the pollen, and this may be dependent on *AQP* genes, although they are not directly related to pollen grain germination. Our results provide candidate abundantly expressed *BrPIP* genes in flower, which may play a role in the control of pollen rehydration, which is an essential step for the success of pollination.

Our expression analysis showed that *BrPIP* genes are expressed differently upon various abiotic stress treatments. In response to cold stress, all *BrPIP* genes showed down-regulation, except *BrPIP, 1;3b, 1;5, 2;4a, 2;6, 2;7a, 2;7b* and *2;7c* in Kenshin (Fig. 6a). Interestingly, *BrPIP2;6* showed 10-fold higher expression compared to the control at 12 h in Kenshin. By contrast, most of the *BrPIP* genes showed up regulation in Chiifu and exhibited higher expression at 12 h. All of the genes showed several-fold higher expression in Chiifu compared to Kenshin. In summary, the *BrPIP* genes were more highly induced than any other group of *BrAQP* genes in response to cold or freezing stress. These results are expected due to the origin of two lines, where Chiifu is cold tolerant and Kenshin is cold susceptible [40]. Plasma membrane *AQP* genes have been reported to play roles under both low and freezing temperatures in rice [18]. *AQP* genes also function to maintain homeostasis and water balance under stress conditions [41]. The expression of specific *AQPs* is high in guard cells [42, 43]; therefore, it seems that *AQPs* play a role in water movement in guard cells, and regulate stomatal movement. Under low temperature conditions, leaf stomata of cold-sensitive plants remain open but those of cold-tolerant plants close rapidly [44, 45] and maintain cell turgor pressure. All *BrPIP* genes showed higher expression in cold-tolerant Chiifu than in cold-susceptible Kenshin lines. Therefore, we speculate that *BrPIP* genes might be involved in maintenance of water balance in the cell and cell turgor pressure during cold stress.

We found that the majority of *BrPIP* genes were significantly down-regulated during drought stress treatment (Fig. 6b). Mittler et al. [46] reported that quick accumulation of reactive oxygen species (ROS) leads to damage of the cell membrane and oxidation of proteins, lipids, and DNA during drought stress. Down-regulation of *BrPIP* gene expression during drought stress may reduce membrane water permeability and cellular water conservation during dehydration periods. In agreement with our findings, the *MIP* genes in *Nicotiana glauca* [47] and *PIP* genes in *Arabidopsis* [20] were down-regulated under drought stress. By contrast, very few *BrPIP* genes displayed up-regulation and showed higher expression at 4 or 12 h (Fig. 6b). Notably, *BrPIP2;4a* and *2;4b* exhibited 4- and 7-fold higher expression, respectively, compared to the control. In addition, over-expression of *AQP7* in tobacco plants and *MaPIP1;1* in banana plants reduced membrane injury compared to wild-type plants under drought stress [48, 49]. These results indicate that up-regulated *BrPIP* genes might participate in avoiding membrane injury under drought stress.

Muries et al. [50] reported that 3 *AQP*s genes showed low expression in roots and were highly expressed in leaves and/or flowers, and remained stable or were up-regulated under drought. This result indicated that the *AQP* genes that are down regulated under normal condition can be highly expressed in drought stress in roots. This pattern might be due to the existence of post transcriptional mechanisms regulating PIP trafficking to the plasma membrane to overcome the drought via decreasing injury of the membrane. Therefore, it is necessary to take root samples in addition to leaf samples under drought stress conditions for expression profiling of *BrPIP* genes in order to make decisive conclusions for development of drought tolerant cultivars. Otherwise, the transcriptional down-regulation of PIP genes upon drought stress could also be observed on the protein level [51].

Under salt stress, all of the *BrPIP* genes were up-regulated except *BrPIP1;1a* and *BrPIP1;1b.* However, most of the *BrPIP* genes showed initial down-regulation and subsequent up-regulation, and highest expression was observed at 24 h (Fig. 6c). During salt stress, the initial down- and subsequent up-regulation of *BrPIP* gene expression indicate that these genes likely function in limiting water loss at the early stage and subsequent water uptake to maintain homeostasis in the cell. Early down-regulation and subsequent up-regulation of *AQP* gene expression has also been observed in microarray analysis of the two rice cultivars [52] and *Arabidopsis* [53].

*AQP* genes have been identified to play important roles in ABA responses in different plant species including *Arabidopsis* [12], rice [54], *Brassica napus* [55], and radish [1]. All of the *BrPIP* genes except *BrPIP1;2b; 2;3b* and *2;5a* were up-regulated in response to exogenous ABA application (Fig. 6d). Most of the *BrPIP* genes showed moderate up-regulation (below 3 fold). However, the *BrPIP1;5, 2;4a, 2;4b, 2;6,* and *2;7a* exhibited 9-, 16-, 5-, 4- and 4- fold higher expression, respectively, in response to ABA treatment. These results indicate that responsiveness of *BrPIP* genes to ABA treatment varied greatly. Therefore, it could be deduced that *BrPIP* gene expression responses are complex, likely due to involvement in both ABA-dependent and ABA-independent signaling pathways.

Under water logging stress, all of the *BrPIP* genes were significantly down-regulated. A very few cases showed up-regulation at the end of the time courses, although their expression pattern remained below the control (Fig. 6e). The hydraulic conductivity of tissues is regulated by three different pathways of water flow in plants, the symplastic, transcellular and apoplastic pathways [56]. In the symplastic pathway, water and solutes are transported from cytoplasm of one cell to that of a neighboring cell via plasmodesmata. In the transcellular pathway, water and dissolved nutrients pass across through plasma membrane and vacuolar membrane. The apoplastic pathway facilitates the transport of water and solutes across cell wall. Apoplastic water movement is faster than symplastic water movement. Under water logging conditions, apoplastic water movement may be more active and the symplastic water movement system may be stop or inactive. *AQPs* are mostly involved in symplastic water transport in plants [57, 58], consistent with our findings that all *BrPIP* genes showed down-regulation under water logging, when symplastic water movement would be expected to be down-regulated.

The cold-upregulated *AQP* genes such as *BrPIP1;4* could be candidates for introgression or overexpression to develop cold stress tolerant genotypes, whereas *BrPIP1;5* genes might candidates for cold as well as ABA-responsive *B. rapa*. The *BrPIP* gene *BrPIP2;6* was cold- and *Fusarium*-stress responsive; *Br.PIP*2*;7c* was cold- and salt-stress responsive; *BrPIP2;4a* was drought- and ABA-responsive. In addition, to obtain drought and salt stress-tolerant genotypes, breeders might focus attention on *BrPIP*2;*4b. BrPIP1;3b* could be useful for salt and *Fusarium* fungus tolerance. Additionally, to develop *Fusarium* fungus tolerance, introgression of *BrPIP2;1* and *BrPIP2;2a* might be useful (Fig. 6a-f). Our findings are also supported by the review of Afzal et al. [59] the argues that *AQP* genes play an important role in plant defense responses against biotic and abiotic stressors and the report of Reddy et al. [60] of the functions of this gene family in abiotic stress tolerance in Sorghum.

There have been no previous reports on responses of *AQP* to biotic stress. From our analysis, we have identified 4 *BrPIP* genes that showed responsiveness to biotic stress in the form of *Fusarium oxysporum* f.sp*. conglutinans* fungus. Three *BrPIP* genes showed the highest expression at 4 dai, and one showed the highest expression at 11 dai (Fig. 6f). This soil pathogenic fungus specifically attacks *Brassica* species, causing wilting, yellowing, necrosis of various plant parts and finally plant death [61]. The highly responsive *BrPIP* genes reported here might play an important role against the fungus *F. oxysporum* f.sp. *conglutinans*.

## Conclusions

In this study, we demonstrated that *BrPIP* genes showed organ-specific expression in *B. rapa* plants and might be related to different cellular controls of water flow. In addition, four out of 22 *BrPIP* genes showed responses to *F. oxysporum* f.sp. *conglutinans* fungal infection in *B. rapa* plants. Our expression analysis illustrates the possible involvement of *BrPIP* genes in different abiotic and biotic stress-related physiological processes. Several *BrPIP* genes seem to participate in multiple processes; for instance, *BrPIP1;3b, 1;4,2;4a, 2;6, 2;7a* showed responsiveness to cold and drought stresses. *BrPIP1;3b, 1;4, 2;4a, 2;4b, 2;6* and *2;7a* showed higher expression under salt and drought stresses and might be useful for developing salt and drought tolerance cultivars through conventional, molecular or transgenic breeding approaches. By contrast, *BrPIP1;4; 1;5, 2;3b,2;4a,2;5b,2;6, 2;7a, 2;7b* and *2;7c* genes exhibited several-fold higher expression compared to the control during cold and salt stresses. Remarkably, *BrPIP1;3a, 1;4, 2;4a,2;6* and *2;7a* exhibited responses to three abiotic stress (cold, salt and drought) and could be good sources for breeding targeted abiotic stress-tolerant cultivars. It is interesting to note that all *BrPIP* genes were significantly down-regulated by water logging stress, while *BrPIP1;5* and *2;4a* showed the highest expression to ABA treatment. The highly induced *BrPIP* genes reported here might be involved in maintaining water homeostasis in plant responses to abiotic stresses and ABA, and several of these genes might be functional against multiple stresses. The comprehensive expression analysis under different stress stimuli supplies novel information to assign putative stress-related physiological functions of *BrPIP* genes and facilitates selection of potential genes for further functional genomics studies in different *Brassica* crops.

## Methods

### Identification and sequence analysis of aquaporins in *B. rapa*

*B. rapa* AQP members were identified using the key word “aquaporin” for the SWISSPROT tool of the *B. rapa* database (http://brassicadb.org/brad/index.php; [22]. We also investigated the microarray annotated database for two cold-treated *B. rapa* inbred lines, Chiifu and Kenshin, using the keyword “aquaporin”. The CDS (coding DNA sequence) and protein sequences of the identified AQPs were processed or deduced using the *B. rapa* genomic database, after which the AQP protein sequences were further examined to confirm the presence of the characteristic MIP and trans-membrane helical domains using the SMART program (http://smart.embl-heidelberg.de/; [62] and TMHMM Server v.2.0 (http://www.cbs.dtu.dk/services/TMHMM/) [63]. Prediction of subcellular localization of identified *B. rapa* AQPs was carried out using Plant-mPLoc (http://www.csbio.sjtu.edu.cn/bioinf/plant/). Additionally, the primary gene structure (protein length, molecular weight and iso-electric point) was analyzed using ExPasy (http://au.expasy.org/tools/pi_tool.html). Open reading Frame Finder (ORF) was obtained using ORF finder at NCBI (http://www.ncbi.nlm.nih.gov/gorf/gorf.html). Multiple sequence alignments using the identified protein sequences were made by CLUSTAL Omega (http://www.ebi.ac.uk/Tools/msa/clustalo/). The protein homology study was done using the Basic Local Alignment Search Tool (BLASTp) (http://www.ncbi.nlm.nih.gov/BLAST/) to confirm the identified *AQP* genes. The exon–intron organization of *BrAQP* genes was identified by comparing predicted coding sequences (CDS) with the corresponding genomic sequences using the GSDS 2.0 software (http://gsds.cbi.pku.edu.cn). The conserved motifs in the encoded proteins were identified using Multiple Expectation Maximization for Motif Elicitation (MEME; http://meme-suite.org/tools/meme) with the following parameters: maximum number of motifs 10; width of optimum motif ≥15 and ≤50.

### Phylogenetic analysis

The predicted protein sequences of the 59 *BrAQP* genes were downloaded from the *B. rapa* genomic database (http://brassicadb.org/brad/). *Arabidopsis* and tomato AQP protein sequences were collected from TAIR (http://www.arabidopsis.org/) and the Sol Genomics network (http://solgenomics.net/), respectively. All sequences were then aligned using Clustal X [64]. A phylogenic tree was constructed with MEGA6.0 software (http://www.megasoftware.net) [65, 66] using the neighbor-joining method and 1,000 bootstrap replicates. The different domains might contribute to the topology of the phylogenetic tree with pairwise gap deletion option.

### Chromosomal location and gene duplication analysis

Sub-genome fractionation, and positional information of all candidate *AQP* genes along through the ten (10) chromosomes of *B. rapa* were retrieved from *B. rapa* database and the locations of the *AQP* genes were drafted using Map Chart version 2.2 (http://www.wageningenur.nl/en/show/Mapchart.htm). The *AQP* genes were BLAST searched (http://blast.ncbi.nlm.nih.gov/Blast.cgi) against each other to identify duplicate genes, in which the similarity of the aligned regions covered >80% and the aligned region had identity >80% [67]. Tandem duplicated genes were defined as an array of two or more homologous genes within a range of 100-kb distance. We calculated the non-synonymous substitution (*Ka*), synonymous rate (*Ks*), and evolutionary constriction (*Ka/Ks*) between the duplicated *AQP* gene pairs of *B. rapa* based on their coding sequence alignments, using the Nei and Gojobori model [68] as employed in MEGA 6.0 software (66). The nonsynonymous to synonymous ratio (*Ka/Ks*) between duplicated genes was analyzed to identify the mode of selection. *Ka/Ks* ratio >1, <1 and =1 indicate positive selection, purifying selection and neutral selection, respectively. We calculated the divergence time of duplicated gene pairs using *T* = *Ks/2R* Mya (Millions of years), where *T* refers to divergence time, *Ks* refers to the synonymous substitutions per site, and *R* is the rate of divergence of plant’s nuclear genes. For dicotyledonous plants *R* = 1.5 × 10^−8^ synonymous substitutions per site per year (38).

### Microarray expression analysis

Temperature-treated microarray data for *AQP* genes were collected from the data of Jung et al. (30). For that data, two inbred lines of *B. rapa* ssp. *pekinensis*, namely cold-tolerant Chiifu and cold-sensitive Kenshin, were treated with different temperatures viz*. 22,* 4, 0, −2, and −4 °C for 2 h. A heat map was generated based on transcript abundance value of 59 AQP genes using Cluster 3.0 and tree view software (http://bonsai.hgc.jp/~mdehoon/software/cluster/software.htm#ctv).

### Microsynteny analysis of the AQP gene family

The microsyntenic relationship of AQP genes among *B. rapa, B. oleracea* and *A. thaliana* were detected using Blast against whole genomes of such crop species. *AQP* gene positions on chromosomes were collected from databases and the relationship among the three crop species were plotted using Circos software (http://circos.ca/) [69].

### Plant materials, growth and treatments

Chinese cabbage (*B. rapa* ssp. *pekinensis*) inbred lines cold-tolerant Chiifu and cold-sensitive Kenshin were used for cold-stress experiments, and Kenshin was used for other abiotic stress treatments. Seed sterilization, culture, seedling management were conducted according to the methods described by Ahmed et al. [70]. Plants were culture on semisolid media for 2 weeks, after which those plants were transferred into liquid media to minimize stress during the treatment time. The 3-week-old plants were used for abiotic stress treatments (cold, drought, salt, ABA and water logging) and treatments were applied over a continuous time course (with samples taken at 0, 1, 4, 12, 24 and 48 h). Plants were transferred to the incubator at 4 °C to induce cold stress. Drought stress was simulated by drying the plants on Whatmann 3 mm filter papers. To induce salt ABA and waterlogging stress, plants were placed on petri dishes with medium containing 200 mM NaCl, 100 mM abscisic acid (ABA) and abundant of water respectively, for the recommended time courses. Fresh roots and leaves (third and fourth leaves) of *B. rapa* plants were harvested, immediately frozen in liquid nitrogen, and then stored at −80 ° C for RNA extraction. *B. rapa* (SUN-3061) was used for analysis of organ-specific expression and for biotic stress treatment (with *F. oxysporum* f.sp. *conglutinans*). The plants were grown for 3 weeks under culture room conditions with 16 h light and 8 h dark maintaining 25 °C temperature prior to fungus treatment. The fungal spore concentration 1x10^6^ spores per ml solution was used for inoculation using the method described by Ahmed et al. [71]. Samples were collected from infected and mock-infected plants at 0 h, 3 h, 6 h, 4 d, 8 d and 11 d after inoculation (dai). The local (fourth) and systemic (fifth) leaves were harvested and immediately frozen in liquid nitrogen. Samples were then stored at −80 ° C until RNA extraction.

### RNA extraction and cDNA synthesis

Total RNA was extracted from the samples (roots and leaves) using the RNeasy mini kit (Qiagen, USA) following the manufacturer’s protocol. The concentration of RNA from each sample was determined by UV spectrophotometry at A260 using a NanoDropND-1000 (Nano Drop Technologies, USA). DNA contamination was removed using RNase-free DNase (Promega, USA) following the manufacturer’s protocol. A 6 μl sample of total RNA was converted to cDNA using the First-Strand cDNA synthesis kit (Invitrogen, Japan) following the manufacturer’s instructions.

### qPCR expression analysis

For each treatment, qRT-PCR was performed on three biological replicates. The 10 μl reaction volume consisted of the following: 5 μl 2x Quanti speed SYBR mix, 1 μL (10 pmol) each forward (F) and reverse (R) gene-specific primers, 1 μl template cDNA (50 ng) and 2 μl distilled, deionized water (ddH_2_O). The conditions for real-time PCR were as follows: initial denaturation at 95 °C for 5 min, followed by 40 cycles of denaturation at 95 °C for 10 s, annealing at 58 °C for 10 s, and extension at 72 °C for 15 s. The qRT-PCR reactions were normalized using the *B. rapa Actin* gene as reference for all comparisons [72]. The fluorescence was measured following the last step of each cycle, and three replications were used for each sample. Amplification detection and data were processed using the Light cycler® 96 SW 1.1 software and the cq value was calculated using the 2^-ΔΔ^C_T_ method to determine the relative expression. The relative expression data was statistically analyzed (Tukey HSD test) and lettering was done using Minitab 17 software (https://www.minitab. com/products/minitab/).

## Additional files

Additional file 1: Table S1.
*In silico* analysis of aquaporin genes identified in *B. rapa* with their closest *Arabidopsis* homologs and sequence characteristics. (DOCX 21 kb)
Additional file 2: Table S2. Homology analysis of *AQP* genes of *B. rapa*. (DOCX 66 kb)
Additional file 3: Figure S1. Alignment of amino acid sequences of *Arabidopsis* and (1a) BrNIP (1b) BrSIP (1c) BrPIP and (1d) BrTIP subfamily members. Upper red line indicates predicted MIP domain and the blue portion of the alignment denotes predicted transmembrane domains. The two conserved NPA motifs are shown in bold pink letters. Residues comprising the ar/R filter are marked in yellow and labelled H2, H5, LE1 and LE2. Residues occupying the conserved Froger’s positions one to five (from N- to C-terminus P1 to P5) are marked in green. (PPTX 437 kb)
Additional file 4: Table S3. Pair-wise sequences similarity (%) of aquaporin proteins in *B. rapa. (XLSX 32 kb)*

Additional file 5: Figure S2. Depiction of *BrAQP* duplicated gene pairs on *10* chromosomes of *B. rapa*. (PPTX 400 kb)
Additional file 6: Figure S3. Schematic representation of motif compositions in the BrAQP protein sequences. Different motifs, numbered 1–10, are displayed in different colored boxes. The names of all members are displayed on the left, while the length of the motif is shown in the scale at the bottom of the figure. (PPTX 269 kb)
Additional file 7: Table S4. Best possible match sequences of motifs (1–15) presented in Additional file Figure S3. (DOCX 11 kb)
Additional file 8: Figure S4. The intron-exon structures of *BrAQP* genes. Names of the genes are on the left. The thick blue lines, exons; fine red lines, introns. (PPTX 346 kb)
Additional file 9: Figure S5. Expression profiles of *BrPIP* genes in various tissues as determined by RT-PCR analyses. Four amplified bands from left to right for each gene represent amplified products from R, roots; S, stems; L, leaves; Fb, flower buds (PPTX 103 kb)
Additional file 10: Table S5. Primer sequence used for real time and RT-PCR amplification of *Aquaporin* genes of *B. rapa. (DOCX 12 kb)*

## Abbreviations

ABA: Abscisic acid
AK: Karyotype
AQP: Aquaporin
Ar/R: Aromatic/ Arginine
At: *Arabidopsis thaliana*
B. oleracea: *Brassica oleracea*
Br: *Brassica rapa*
BRAD: Brassica database
C: Chiifu
CDS: Coding DNA sequence
Cl: Cluster
dai: Days after infection
*F.oxysporum*: *Fusarium oxysporum*
f.sp: Fungal species
Gm: *Glycine max*
K: Kenshin
Ka: Nonsynonymous substitutions per nonsynonymous site
kb: Kilo basepair
Ks: Synonymous substitutions per synonymous site
LF: Least fractionated
MF1: Medium fractionated
MF2: Most fractionated
MIP: Major intrinsic protein
MYA: Million year
NIP: NOD26-like intrinsic protein
Nt: *Nicotiana tabacum*
PIP: Plasma membrane intrinsic proteins
qPCR: Quantitative polymerase chain reaction
ROS: Reactive oxygen species
RT-PCR: Reverse transcription polymerase chain reaction
SIP: Small basic intrinsic protein
Sl: *Solanum lycopersicum*
Ta: *Triticum aestrivum*
TIP: Tonoplast intrinsic protein
XIP: X intrinsic protein
