# Supplementary material for: Genome-wide expression profiling of aquaporin genes confer responses to abiotic and biotic stresses in Brassica rapa
Source: BMC Plant Biol. 2017 Jan 25;17:23. doi: 10.1186/s12870-017-0979-5 (PMC5264328; doi:10.1186/s12870-017-0979-5)
Supplement: Additional file 1: Table S1. — In silico analysis of aquaporin genes identified in B. rapa with their closest Arabidopsis homologs and sequence characteristics. (DOCX 21 kb) [file 12870_2017_979_MOESM1_ESM.docx]

**Table S1.** In silico analysis of aquaporin genes identified in *B. rapa* with their closest *Arabidopsis* homologs and sequence characteristics.

|  |  | Chromosome | | |  | | | | | |
| --- | --- | --- | --- | --- | --- | --- | --- | --- | --- | --- |
| Gene Name | Gene ID | Number | start | end | strand | Protein  length | Isoelectic point  (Pi) | Mw (KDa) | ORF | At ortholog |
| BrSIP1;1a | [Bra040150](javascript:modalDialog('multiSearch.php?gene=Bra040150','select%20database',390,200)) | A01 | 26056127 | 26057962 | + | 254 | 9.93 | 27.24 | 765 | *AtSIP1;1* |
| BrSIP1;1b | [Bra031946](javascript:modalDialog('multiSearch.php?gene=Bra031946','select%20database',390,200)) | A05 | 24507091 | 24508703 | + | 239 | 9.52 | 25.61 | 720 | *AtSIP1;1* |
| BrSIP1;2 | [Bra002151](javascript:modalDialog('multiSearch.php?gene=Bra002151','select%20database',390,200)) | A10 | 11295303 | 11296851 | + | 243 | 10.13 | 26.18 | 732 | *AtSIP1;2* |
| BrSIP2;1a | [Bra014661](javascript:modalDialog('multiSearch.php?gene=Bra014661','select%20database',390,200)) | A04 | 2100218 | 2101863 | + | 237 | 9.49 | 25.90 | 714 | *AtSIP2;1* |
| BrSIP2;1b | [Bra003257](javascript:modalDialog('multiSearch.php?gene=Bra003257','select%20database',390,200)) | A07 | 15500412 | 15501702 | - | 238 | 9.61 | 26.03 | 717 | *AtSIP2;1* |
| BrSIP2;1c | [Bra007285](javascript:modalDialog('multiSearch.php?gene=Bra007285','select%20database',390,200)) | A09 | 29885479 | 29886353 | - | 234 | 9.49 | 25.49 | 705 | *AtSIP2;1* |
| BrNIP1;2a | [Bra013361](javascript:modalDialog('multiSearch.php?gene=Bra013361','select%20database',390,200)) | A01 | 5206437 | 5208447 | + | 297 | 7.76 | 31.64 | 894 | *AtNIP1;2* |
| BrNIP1;2b | [Bra012567](javascript:modalDialog('multiSearch.php?gene=Bra012567','select%20database',390,200)) | A03 | 23528140 | 23532627 | + | 298 | 8.61 | 31.69 | 897 | *AtNIP1;2* |
| BrNIP2;1a | [Bra005430](javascript:modalDialog('multiSearch.php?gene=Bra005430','select%20database',390,200)) | A05 | 5438497 | 5439599 | + | 287 | 8.79 | 30.38 | 864 | *AtNIP2;1* |
| BrNIP2;1b | [Bra005428](javascript:modalDialog('multiSearch.php?gene=Bra005428','select%20database',390,200)) | A05 | 5432795 | 5434118 | + | 286 | 8.57 | 30.32 | 861 | *AtNIP2;1* |
| BrNIP3;1a | [Bra035520](javascript:modalDialog('multiSearch.php?gene=Bra035520','select%20database',390,200)) | A08 | 8142007 | 8143955 | - | 323 | 6.3 | 34.55 | 972 | *AtNIP3;1* |
| BrNIP3;1b | [Bra033867](javascript:modalDialog('multiSearch.php?gene=Bra033867','select%20database',390,200)) | A05 | 15593678 | 15595314 | - | 337 | 8.54 | 36.08 | 1014 | *AtNIP3;1* |
| BrNIP4;1 | [Bra025437](javascript:modalDialog('multiSearch.php?gene=Bra025437','select%20database',390,200)) | A04 | 9093730 | 9096110 | + | 276 | 7.62 | 29.60 | 831 | *AtNIP4;1* |
| BrNIP4;2a | [Bra028151](javascript:modalDialog('multiSearch.php?gene=Bra028151','select%20database',390,200)) | A04 | 6550742 | 6552202 | + | 283 | 8.24 | 30.24 | 852 | *AtNIP4;1* |
| BrNIP4;2b | [Bra025436](javascript:modalDialog('multiSearch.php?gene=Bra025436','select%20database',390,200)) | A04 | 9102623 | 9103526 | + | 197 | 9.77 | 20.82 | 594 | *AtNIP4;1* |
| BrNIP4;2c | [Bra025435](javascript:modalDialog('multiSearch.php?gene=Bra025435','select%20database',390,200)) | A04 | 9109108 | 9110011 | + | 164 | 9.93 | 17.56 | 495 | *AtNIP4;1* |
| BrNIP5;1a | [Bra033181](javascript:modalDialog('multiSearch.php?gene=Bra033181','select%20database',390,200)) | A02 | 16960448 | 16963277 | + | 301 | 8.66 | 31.14 | 906 | *AtNIP5;1* |
| BrNIP5;1b | [Bra000710](javascript:modalDialog('multiSearch.php?gene=Bra000710','select%20database',390,200)) | A03 | 12602344 | 12609091 | - | 301 | 8.9 | 31.21 | 906 | *AtNIP5;1* |
| BrNIP6;1a | [Bra008442](javascript:modalDialog('multiSearch.php?gene=Bra008442','select%20database',390,200)) | A02 | 14668782 | 14670332 | + | 305 | 7.69 | 31.83 | 918 | *AtNIP6;1* |
| BrNIP6;1b | [Bra035156](javascript:modalDialog('multiSearch.php?gene=Bra035156','select%20database',390,200)) | A07 | 25684748 | 25686188 | + | 305 | 8.57 | 31.82 | 918 | *AtNIP6;1* |
| BrNIP7;1 | [Bra020777](javascript:modalDialog('multiSearch.php?gene=Bra020777','select%20database',390,200)) | A05 | 24084892 | 24086510 | + | 272 | 6.57 | 28.60 | 819 | *AtNIP7;1* |
| BrTIP1;1 | [Bra017222](javascript:modalDialog('multiSearch.php?gene=Bra017222','select%20database',390,200)) | A04 | 16043764 | 16044882 | + | 251 | 6.02 | 25.55 | 756 | *AtTIP1;1* |
| BrTIP1;2a | [Bra025210](javascript:modalDialog('multiSearch.php?gene=Bra025210','select%20database',390,200)) | A06 | 21676099 | 21677177 | + | 253 | 5.61 | 25.77 | 762 | *AtTIP1;2* |
| BrTIP1;2b | [Bra032937](javascript:modalDialog('multiSearch.php?gene=Bra032937','select%20database',390,200)) | A02 | 21684584 | 21685852 | - | 253 | 5.32 | 25.79 | 762 | *AtTIP1;2* |
| BrTIP1;3 | [Bra037415](javascript:modalDialog('multiSearch.php?gene=Bra037415','select%20database',390,200)) | A09 | 516607 | 517365 | - | 252 | 5.35 | 25.86 | 759 | *AtTIP1;3* |
| BrTIP2;1a | [Bra027181](javascript:modalDialog('multiSearch.php?gene=Bra027181','select%20database',390,200)) | A05 | 20281490 | 20282983 | - | 248 | 5.3 | 24.86 | 747 | *AtTIP2;1* |
| BrTIP2;1b | [Bra001626](javascript:modalDialog('multiSearch.php?gene=Bra001626','select%20database',390,200)) | A03 | 17533686 | 17535004 | + | 249 | 5.58 | 24.89 | 750 | *AtTIP2;1* |
| BrTIP2;1c | [Bra021171](javascript:modalDialog('multiSearch.php?gene=Bra021171','select%20database',390,200)) | A01 | 22765495 | 22766645 | - | 210 | 5.66 | 20.98 | 633 | *AtTIP2;1* |
| BrTIP2;2 | [Bra026245](javascript:modalDialog('multiSearch.php?gene=Bra026245','select%20database',390,200)) | A01 | 10224550 | 10225424 | - | 250 | 5.1 | 24.99 | 753 | *AtTIP2;2* |
| BrTIP2;3a | [Bra024943](javascript:modalDialog('multiSearch.php?gene=Bra024943','select%20database',390,200)) | A06 | 23263655 | 23264989 | + | 251 | 4.98 | 25.25 | 756 | *AtTIP2;3* |
| BrTIP2;3b | [Bra022131](javascript:modalDialog('multiSearch.php?gene=Bra022131','select%20database',390,200)) | A02 | 19270257 | 19278307 | - | 204 | 4.9 | 20.68 | 615 | *AtTIP2;3* |
| BrTIP3;1a | [Bra008079](javascript:modalDialog('multiSearch.php?gene=Bra008079','select%20database',390,200)) | A02 | 12029940 | 12030888 | + | 266 | 6.75 | 28.00 | 801 | *AtTIP3;1* |
| BrTIP3;1b | [Bra016014](javascript:modalDialog('multiSearch.php?gene=Bra016014','select%20database',390,200)) | A07 | 23117218 | 23118268 | + | 265 | 6.54 | 28.00 | 798 | *AtTIP3;1* |
| BrTIP3;2a | [Bra025947](javascript:modalDialog('multiSearch.php?gene=Bra025947','select%20database',390,200)) | A06 | 6553605 | 6554761 | + | 267 | 6.49 | 28.58 | 804 | *AtTIP3;2* |
| BrTIP3;2b | [Bra031005](javascript:modalDialog('multiSearch.php?gene=Bra031005','select%20database',390,200)) | A09 | 34718073 | 34719224 | - | 267 | 6.54 | 28.46 | 804 | *AtTIP3;2* |
| BrTIP4;1 | [Bra034271](javascript:modalDialog('multiSearch.php?gene=Bra034271','select%20database',390,200)) | A04 | 11930123 | 11931805 | + | 249 | 5.3 | 26.10 | 750 | *AtTIP4;1* |
| BrTIP5;1 | [Bra018148](javascript:modalDialog('multiSearch.php?gene=Bra018148','select%20database',390,200)) | A06 | 10325804 | 10326748 | - | 255 | 6.05 | 26.30 | 768 | *AtTIP5;1* |
| BrPIP1;1a | [Bra007603](javascript:modalDialog('multiSearch.php?gene=Bra007603','select%20database',390,200)) | A09 | 31490618 | 31492001 | + | 286 | 8.86 | 30.72 | 861 | *AtPIP1;1* |
| BrPIP1;1b | [Bra014437](javascript:modalDialog('multiSearch.php?gene=Bra014437','select%20database',390,200)) | A04 | 649779 | 651115 | - | 286 | 9.16 | 30.71 | 861 | *AtPIP1;1* |
| BrPIP1;2a | [Bra039301](javascript:modalDialog('multiSearch.php?gene=Bra039301','select%20database',390,200)) | A04 | 18807721 | 18809169 | + | 280 | 8.88 | 30.15 | 843 | *AtPIP1;2* |
| BrPIP1;2b | [Bra004950](javascript:modalDialog('multiSearch.php?gene=Bra004950','select%20database',390,200)) | A05 | 2615972 | 2617343 | + | 286 | 9.1 | 30.26 | 861 | *AtPIP1;2* |
| BrPIP1;3a | [Bra032644](javascript:modalDialog('multiSearch.php?gene=Bra032644','select%20database',390,200)) | A09 | 38760441 | 38761505 | - | 286 | 9.03 | 30.54 | 861 | *AtPIP1;3* |
| BrPIP1;3b | [Bra033248](javascript:modalDialog('multiSearch.php?gene=Bra033248','select%20database',390,200)) | A10 | 3345854 | 3346893 | - | 286 | 9.03 | 30.52 | 861 | *AtPIP1;3* |
| BrPIP1;4 | [Bra000974](javascript:modalDialog('multiSearch.php?gene=Bra000974','select%20database',390,200)) | A03 | 14235366 | 14236625 | + | 288 | 9.14 | 30.75 | 867 | *AtPIP1;4* |
| BrPIP1;5 | [Bra019307](javascript:modalDialog('multiSearch.php?gene=Bra019307','select%20database',390,200)) | A03 | 25092063 | 25093184 | + | 287 | 8.99 | 30.76 | 864 | *AtPIP1;5* |
| BrPIP2;1 | [Bra006997](javascript:modalDialog('multiSearch.php?gene=Bra006997','select%20database',390,200)) | A09 | 28252411 | 28253995 | - | 287 | 7.68 | 30.40 | 864 | *AtPIP2;1* |
| BrPIP2;2a | [Bra023102](javascript:modalDialog('multiSearch.php?gene=Bra023102','select%20database',390,200)) | A03 | 8711706 | 8712834 | - | 283 | 6.51 | 30.03 | 852 | *AtPIP2;2* |
| BrPIP2;2b | [Bra005215](javascript:modalDialog('multiSearch.php?gene=Bra005215','select%20database',390,200)) | A05 | 4067080 | 4068219 | - | 238 | 6.81 | 25.29 | 717 | *AtPIP2;2* |
| BrPIP2;3a | [Bra023103](javascript:modalDialog('multiSearch.php?gene=Bra023103','select%20database',390,200)) | A03 | 8716382 | 8717546 | + | 285 | 6.95 | 30.27 | 858 | *AtPIP2;3* |
| BrPIP2;3b | [Bra005216](javascript:modalDialog('multiSearch.php?gene=Bra005216','select%20database',390,200)) | A05 | 4071511 | 4072570 | + | 231 | 9.12 | 24.27 | 696 | *AtPIP2;3* |
| BrPIP2;4a | [Bra020238](javascript:modalDialog('multiSearch.php?gene=Bra020238','select%20database',390,200)) | A02 | 4764651 | 4766078 | + | 283 | 8.22 | 29.97 | 852 | *AtPIP2;4* |
| BrPIP2;4b | [Bra006650](javascript:modalDialog('multiSearch.php?gene=Bra006650','select%20database',390,200)) | A03 | 4475470 | 4476900 | + | 285 | 7.62 | 30.12 | 858 | *AtPIP2;4* |
| BrPIP2;4c | [Bra002462](javascript:modalDialog('multiSearch.php?gene=Bra002462','select%20database',390,200)) | A10 | 9450456 | 9451814 | - | 260 | 6.88 | 27.29 | 783 | *AtPIP2;4* |
| BrPIP2;5a | [Bra007100](javascript:modalDialog('multiSearch.php?gene=Bra007100','select%20database',390,200)) | A09 | 28837381 | 28839703 | + | 286 | 8.99 | 30.47 | 861 | *AtPIP2;5* |
| BrPIP2;5b | [Bra003196](javascript:modalDialog('multiSearch.php?gene=Bra003196','select%20database',390,200)) | A07 | 15094951 | 15097025 | + | 286 | 8.77 | 30.49 | 861 | *AtPIP2;5* |
| BrPIP2;6 | [Bra000111](javascript:modalDialog('multiSearch.php?gene=Bra000111','select%20database',390,200)) | A03 | 9324457 | 9327238 | + | 288 | 8.6 | 30.87 | 867 | *AtPIP2;6* |
| BrPIP2;7a | [Bra034675](javascript:modalDialog('multiSearch.php?gene=Bra034675','select%20database',390,200)) | A08 | 11342859 | 11344234 | - | 281 | 9.13 | 29.71 | 846 | *AtPIP2;7* |
| BrPIP2;7b | [Bra011585](javascript:modalDialog('multiSearch.php?gene=Bra011585','select%20database',390,200)) | A01 | 1544899 | 1546238 | - | 281 | 8.62 | 29.74 | 846 | *AtPIP2;7* |
| BrPIP2;7c | [Bra017697](javascript:modalDialog('multiSearch.php?gene=Bra017697','select%20database',390,200)) | A03 | 29867761 | 29869141 | + | 281 | 8.62 | 29.80 | 846 | *AtPIP2;7* |
