# Supplementary material for: Genome-wide expression profiling of aquaporin genes confer responses to abiotic and biotic stresses in Brassica rapa
Source: BMC Plant Biol. 2017 Jan 25;17:23. doi: 10.1186/s12870-017-0979-5 (PMC5264328; doi:10.1186/s12870-017-0979-5)
Supplement: Additional file 2: Table S2. — Homology analysis of AQP genes of B. rapa. (DOCX 66 kb) [file 12870_2017_979_MOESM2_ESM.docx]

**Table S2.** Homology analysis of *AQP* genes of *Brassica rapa*.

| **Gene name** | **Accession no.** | **Top matched clone** | **Name of protein** | **Quary cover** | **e-value** | **Identity** | **Holologus species** | **Function** | **Ref.** |
| --- | --- | --- | --- | --- | --- | --- | --- | --- | --- |
| BrSIP1;1a | [Bra040150](javascript:modalDialog('multiSearch.php?gene=Bra040150','select%20database',390,200)) | NP187059 | [Aquaporin SIP1-1](http://blast.ncbi.nlm.nih.gov/Blast.cgi#alnHdr_15229225) | 88% | 3e-126 | 87% | *Arabidopsis thaliana* |  | [(1)](http://www.ncbi.nlm.nih.gov/pubmed?term=Salanoubat%20M%5BAuthor%5D&cauthor=true&cauthor_uid=11130713) |
|  |  | NP001274290 | Aquaporin SIP1;2-like | 94% | 3e-79 | 52% | *Solanum lycopersicum* | Responsive to tissue-specific and development-specific expression; water and solute transport in leaves and during fruit development. | (2) |
| BrSIP1;1b | [Bra031946](javascript:modalDialog('multiSearch.php?gene=Bra031946','select%20database',390,200)) | XP002884428 | [SIP1;1](http://blast.ncbi.nlm.nih.gov/Blast.cgi#alnHdr_297833092) | 100% | 1e-141 | 90% | *Arabidopsis lyrata subsp. lyrata* |  | Unpublished |
|  |  | XP007030233 | Small basic intrinsic protein 1 | 98% | 2e-79 | 63% | *Theobroma cacao* | Responsive to Pod colour | (3) |
| BrSIP1;2 | [Bra002151](javascript:modalDialog('multiSearch.php?gene=Bra002151','select%20database',390,200)) | [NP187059](http://www.ncbi.nlm.nih.gov/protein/15229225?report=genbank&log$=prottop&blast_rank=8&RID=DTJFBUTC01R) | [Aquaporin SIP1;1](http://blast.ncbi.nlm.nih.gov/Blast.cgi#alnHdr_15229225) | 94% | 3e-95 | 67% | *A. thaliana* |  | (1) |
|  |  | [XP007030233](http://www.ncbi.nlm.nih.gov/protein/590641448?report=genbank&log$=prottop&blast_rank=48&RID=C3ACV7U8014) | [Small basic intrinsic protein 1](http://blast.ncbi.nlm.nih.gov/Blast.cgi#alnHdr_590641448) | 97% | 6e-76 | 57% | *T. cacao* | Responsive to Pod colour | (3) |
| BrSIP2;1a | [Bra014661](javascript:modalDialog('multiSearch.php?gene=Bra014661','select%20database',390,200)) | [XP002878121](http://www.ncbi.nlm.nih.gov/protein/297820476?report=genbank&log$=prottop&blast_rank=2&RID=DTA4MZ8M01R) | [SIP2;1](http://blast.ncbi.nlm.nih.gov/Blast.cgi#alnHdr_297820476) | 100% | 2e-152 | 88% | [*A. lyrata subsp. lyrata*](http://blast.ncbi.nlm.nih.gov/Blast.cgi#alnHdr_297820476) |  | Unpublished |
|  |  | [BAO18651](http://www.ncbi.nlm.nih.gov/protein/560891785?report=genbank&log$=prottop&blast_rank=33&RID=BZ4JSP65013) | Small basic intrinsic protein 2;1 | 99% | 1e-99 | 59% | *S. lycopersicum* | Responsive to tissue-specific and development-specific expression; water and solute transport in leaves and during fruit development. | (2) |
| BrSIP2;1b | [Bra003257](javascript:modalDialog('multiSearch.php?gene=Bra003257','select%20database',390,200)) | [XP002878121](http://www.ncbi.nlm.nih.gov/protein/297820476?report=genbank&log$=prottop&blast_rank=2&RID=DTHKH8TB01R) | [SIP2;1](http://blast.ncbi.nlm.nih.gov/Blast.cgi#alnHdr_297820476) | 100% | 8e-154 | 90% | [*A. lyrata subsp. lyrata*](http://blast.ncbi.nlm.nih.gov/Blast.cgi#alnHdr_297820476) |  | Unpublished |
|  |  | [BAO18651](http://www.ncbi.nlm.nih.gov/protein/560891785?report=genbank&log$=prottop&blast_rank=36&RID=C1S917F5016) | [Small basic intrinsic protein 2;1](http://blast.ncbi.nlm.nih.gov/Blast.cgi#alnHdr_560891785) | 100% | 1e-100 | 92% | *S. lycopersicum* | Responsive to tissue-specific and development-specific expression; water and solute transport in leaves and during fruit development. | (2) |
| BrSIP2;1c | [Bra007285](javascript:modalDialog('multiSearch.php?gene=Bra007285','select%20database',390,200)) | [XP002878121](http://www.ncbi.nlm.nih.gov/protein/297820476?report=genbank&log$=prottop&blast_rank=3&RID=DTCNG1VM01R) | [SIP2;1](http://blast.ncbi.nlm.nih.gov/Blast.cgi#alnHdr_297820476) | 100% | 8e-134 | 80% | [*A. lyrata subsp. lyrata*](http://blast.ncbi.nlm.nih.gov/Blast.cgi#alnHdr_297820476) |  | Unpublished |
|  |  | [BAO18651](http://www.ncbi.nlm.nih.gov/protein/560891785?report=genbank&log$=prottop&blast_rank=36&RID=C1S917F5016) | [Small basic intrinsic protein 2;1](http://blast.ncbi.nlm.nih.gov/Blast.cgi#alnHdr_560891785) | 100% | 1e-91 | 57% | *S. lycopersicum* | Responsive to tissue-specific and development-specific expression; water and solute transport in leaves and during fruit development. | (2) |
| BrNIP1;2a | [Bra013361](javascript:modalDialog('multiSearch.php?gene=Bra013361','select%20database',390,200)) | [AAM61294](http://www.ncbi.nlm.nih.gov/protein/21536953?report=genbank&log$=prottop&blast_rank=13&RID=KF148E7S016) | [Major intrinsic protein (MIP)- like](http://blast.ncbi.nlm.nih.gov/Blast.cgi#alnHdr_21536953) | 100% | 00 | 89% | *A.thaliana* |  | Unpublished |
|  |  | [NP001274704](http://www.ncbi.nlm.nih.gov/protein/567757540?report=genbank&log$=prottop&blast_rank=56&RID=BZ5BKNX2013) | [Aquaporin NIP1;1-like](http://blast.ncbi.nlm.nih.gov/Blast.cgi#alnHdr_567757540) | 94% | 6e-128 | 83% | *S. lycopersicum* | Responsive to tissue-specific and development-specific expression; water and solute transport in leaves and during fruit development. | (2) |
| BrNIP1;2b | [Bra012567](javascript:modalDialog('multiSearch.php?gene=Bra012567','select%20database',390,200)) | \|  \|  \| \| --- \| --- \|   [XP010434480](http://www.ncbi.nlm.nih.gov/protein/727516226?report=genbank&log$=prottop&blast_rank=10&RID=KF0N98XC013) | Aquaporin NIP1;2-like | 100% | 00 | 88% | *Camelina sativa* |  | Unpublished |
|  |  | [NP001274704](http://www.ncbi.nlm.nih.gov/protein/567757540?report=genbank&log$=prottop&blast_rank=56&RID=BZ5BKNX2013) | [Aquaporin NIP1;1-like](http://blast.ncbi.nlm.nih.gov/Blast.cgi#alnHdr_567757540) | 94% | 6e-128 | 83% | *S. lycopersicum* | Responsive to tissue-specific and development-specific expression; water and solute transport in leaves and during fruit development. | (2) |
| BrNIP2;1a | [Bra005430](javascript:modalDialog('multiSearch.php?gene=Bra005430','select%20database',390,200)) | ACD75049 | Aquaporin | 100% | 0 | 92% | *A. thaliana* |  | Unpublished |
|  |  | [NP001274704](http://www.ncbi.nlm.nih.gov/protein/567757540?report=genbank&log$=prottop&blast_rank=86&RID=C1UHXN5T016) | Aquaporin NIP1;1 | 98% | 6e-88 | 51% | *S. lycopersicum* | Responsive to tissue-specific and development-specific expression; water and solute transport in leaves and during fruit development. | (2) |
| BrNIP2;1b | [Bra005428](javascript:modalDialog('multiSearch.php?gene=Bra005428','select%20database',390,200)) | [ACD75049](http://www.ncbi.nlm.nih.gov/protein/189032243?report=genbank&log$=prottop&blast_rank=1&RID=DTFN6HX601R) | [Aquaporin](http://blast.ncbi.nlm.nih.gov/Blast.cgi#alnHdr_189032243) | 100% | 6e-179 | 90% | *A. thaliana* |  | Unpublished |
|  |  | [NP001274279](http://www.ncbi.nlm.nih.gov/protein/565324177?report=genbank&log$=prottop&blast_rank=82&RID=C1UZX2NW013) | [Nodulin-26-like](http://blast.ncbi.nlm.nih.gov/Blast.cgi#alnHdr_565324177) | 82% | 5e-89 | 78% | *S. lycopersicum* | Responsive to tissue-specific and development-specific expression; water and solute transport in leaves and during fruit development. | (2) |
| BrNIP3;1a | [Bra035520](javascript:modalDialog('multiSearch.php?gene=Bra035520','select%20database',390,200)) | NP174472 | [Aquaporin NIP3;1](http://blast.ncbi.nlm.nih.gov/Blast.cgi#alnHdr_186479110) | 99% | 0 | 86% | *A. thaliana* |  | Unpublished |
|  |  | NP001274279 | [Nodulin-26-like](http://blast.ncbi.nlm.nih.gov/Blast.cgi#alnHdr_565324177) | 76% | 1e-95 | 59% | *S. lycopersicum* | Responsive to tissue-specific and development-specific expression; water and solute transport in leaves and during fruit development. | (2) |
| BrNIP3;1b | [Bra033867](javascript:modalDialog('multiSearch.php?gene=Bra033867','select%20database',390,200)) | NP174472 | [Aquaporin NIP3;1](http://blast.ncbi.nlm.nih.gov/Blast.cgi#alnHdr_186479110) | 95% | 0 | 78% | *A. thaliana* |  | Unpublished |
|  |  | [NP001274279](http://www.ncbi.nlm.nih.gov/protein/565324177?report=genbank&log$=prottop&blast_rank=47&RID=BTHA5JT301R) | [Nodulin-26-like](http://blast.ncbi.nlm.nih.gov/Blast.cgi#alnHdr_565324177) | 69% | 2e-92 | 61% | *S. lycopersicum* | Responsive to tissue-specific and development-specific expression; water and solute transport in leaves and during fruit development. | (2) |
| BrNIP4;1 | [Bra025437](javascript:modalDialog('multiSearch.php?gene=Bra025437','select%20database',390,200)) | [NP_198597](http://www.ncbi.nlm.nih.gov/protein/15240347?report=genbank&log$=prottop&blast_rank=6&RID=KEEVZUJ701N) | Aquaporin NIP4;1 | 100% | 2e-178 | 87% | *A. thaliana* |  | Unpublished |
|  |  | [BAO18645](http://www.ncbi.nlm.nih.gov/protein/560891773?report=genbank&log$=prottop&blast_rank=28&RID=BY3JBGGJ013) | [Nodulin26-like intrinsic protein 4;1](http://blast.ncbi.nlm.nih.gov/Blast.cgi#alnHdr_560891773) | 98% | 1e-125 | 68% | *S. lycopersicum* | Responsive to tissue-specific and development-specific expression; water and solute transport in leaves and during fruit development. | (2) |
| BrNIP4;2a | [Bra028151](javascript:modalDialog('multiSearch.php?gene=Bra028151','select%20database',390,200)) | [XP002302955](http://www.ncbi.nlm.nih.gov/protein/224069322?report=genbank&log$=prottop&blast_rank=15&RID=DHT21U2S01R) | [Aquaporin, MIP family, NIP subfamily](http://blast.ncbi.nlm.nih.gov/Blast.cgi#alnHdr_224069322) | 98% | 1e-124 | 66% | [*P. trichocarpa*](http://blast.ncbi.nlm.nih.gov/Blast.cgi#alnHdr_224144734) |  | Unpublished |
|  |  | [BAO18645](http://www.ncbi.nlm.nih.gov/protein/560891773?report=genbank&log$=prottop&blast_rank=35&RID=BW4HBHUR013) | [Nodulin26-like intrinsic protein 4;1](http://blast.ncbi.nlm.nih.gov/Blast.cgi#alnHdr_560891773) | 94% | 4e-125 | 86% | *S. lycopersicum* | Responsive to tissue-specific and development-specific expression; water and solute transport in leaves and during fruit development. | (2) |
| BrNIP4;2b | [Bra025436](javascript:modalDialog('multiSearch.php?gene=Bra025436','select%20database',390,200)) | [BAO18645.](http://www.ncbi.nlm.nih.gov/protein/560891773?report=genbank&log$=prottop&blast_rank=16&RID=DHUZGHY7015) | [Nodulin26-like intrinsic protein 4;1](http://blast.ncbi.nlm.nih.gov/Blast.cgi#alnHdr_560891773) | 93% | 2e-91 | 74% | [*S. lycopersicum*](http://blast.ncbi.nlm.nih.gov/Blast.cgi#alnHdr_560891773) | Responsive to tissue-specific and development-specific expression; water and solute transport in leaves and during fruit development. | (2) |
|  |  | [XP007033055](http://www.ncbi.nlm.nih.gov/protein/590652058?report=genbank&log$=prottop&blast_rank=71&RID=BY3YR7UP013) | [Nodulin26-like intrinsic protein 4;1](http://blast.ncbi.nlm.nih.gov/Blast.cgi#alnHdr_560891773) | 91% | 2e-85 | 64% | *T. cacao* | Responsive to Pod colour development | (3) |
| BrNIP4;2c | [Bra025435](javascript:modalDialog('multiSearch.php?gene=Bra025435','select%20database',390,200)) | [BAO18645.](http://www.ncbi.nlm.nih.gov/protein/560891773?report=genbank&log$=prottop&blast_rank=14&RID=DHV54THV014) | [Nodulin26-like intrinsic protein 4;1](http://blast.ncbi.nlm.nih.gov/Blast.cgi#alnHdr_560891773) | 92% | 7e-65 | 59% | [*S. lycopersicum*](http://blast.ncbi.nlm.nih.gov/Blast.cgi#alnHdr_560891773) | Responsive to tissue-specific and development-specific expression; water and solute transport in leaves and during fruit development. | (2) |
|  |  | [XP007033055](http://www.ncbi.nlm.nih.gov/protein/590652058?report=genbank&log$=prottop&blast_rank=67&RID=BY4Z1XRV013) | [Nodulin26-like intrinsic protein 4;1](http://blast.ncbi.nlm.nih.gov/Blast.cgi#alnHdr_560891773) | 90% | 4e-58 | 56% | *T. cacao* | Responsive to Pod colour development | (3) |
| BrNIP5;1a | [Bra033181](javascript:modalDialog('multiSearch.php?gene=Bra033181','select%20database',390,200)) | [NP001105021](http://www.ncbi.nlm.nih.gov/protein/162458955?report=genbank&log$=prottop&blast_rank=34&RID=DHMWXV1F01R) | [Aquaporin NIP3;1](http://blast.ncbi.nlm.nih.gov/Blast.cgi#alnHdr_162458955) | 95% | 2e-136 | 72% | *Zea mays* | Responsive to tissue specific expression | (4) |
|  |  | [ABY19373](http://www.ncbi.nlm.nih.gov/protein/162568623?report=genbank&log$=prottop&blast_rank=62&RID=BTTKTS1101R) | [Major intrinsic protein NIP5;1](http://blast.ncbi.nlm.nih.gov/Blast.cgi#alnHdr_162568623) | 98% | 6e-142 | 71% | *Lotus japonicus* | Responsive to arsenic transportation in crops plant | (5) |
| BrNIP5;1b | [Bra000710](javascript:modalDialog('multiSearch.php?gene=Bra000710','select%20database',390,200)) | [NP001105021](http://www.ncbi.nlm.nih.gov/protein/162458955?report=genbank&log$=prottop&blast_rank=32&RID=DTKZYSCX01R) | [Aquaporin NIP3;1](http://blast.ncbi.nlm.nih.gov/Blast.cgi#alnHdr_162458955) | 95% | 1e-135 | 72% | *Z. mays* | Responsive to tissue specific expression | (4) |
|  |  | [ABY19373](http://www.ncbi.nlm.nih.gov/protein/162568623?report=genbank&log$=prottop&blast_rank=67&RID=C3BN88ME014) | [Major intrinsic protein NIP5;1](http://blast.ncbi.nlm.nih.gov/Blast.cgi#alnHdr_162568623) | 98% | 2e-137 | 69% | *L. japonicus* | Responsive to arsenic transportation in crops plant | (5) |
| BrNIP6;1a | [Bra008442](javascript:modalDialog('multiSearch.php?gene=Bra008442','select%20database',390,200)) | [AFN44229](http://www.ncbi.nlm.nih.gov/protein/394998161?report=genbank&log$=prottop&blast_rank=23&RID=KEBUK98C013) | [Aquaporin](http://blast.ncbi.nlm.nih.gov/Blast.cgi#alnHdr_15220826) | 100% | 6e-167 | 85% | [*Gossypium hirsutum*](http://blast.ncbi.nlm.nih.gov/Blast.cgi#alnHdr_394998161) |  | Unpublished |
|  |  | [XP007023401](http://www.ncbi.nlm.nih.gov/protein/590616066?report=genbank&log$=prottop&blast_rank=17&RID=BZ7T2B3N013) | [Aquaporin NIP6,1](http://blast.ncbi.nlm.nih.gov/Blast.cgi#alnHdr_590616066) | 100% | 3e-169 | 81% | *T. cacao* | Responsive to Pod colour development | (3) |
| BrNIP6;1b | [Bra035156](javascript:modalDialog('multiSearch.php?gene=Bra035156','select%20database',390,200)) | [AFN44229](http://www.ncbi.nlm.nih.gov/protein/394998161?report=genbank&log$=prottop&blast_rank=22&RID=KEBDVE5V013) | [Aquaporin](http://blast.ncbi.nlm.nih.gov/Blast.cgi#alnHdr_15220826) | 100% | 9e-167 | 80% | [*G. hirsutum*](http://blast.ncbi.nlm.nih.gov/Blast.cgi#alnHdr_394998161) |  | Unpublished |
|  |  | [ABY19374](http://www.ncbi.nlm.nih.gov/protein/162568625?report=genbank&log$=prottop&blast_rank=36&RID=BTAHK4DC01R) | Major intrinsic protein NIP6;1 | 99% | 1e-155 | 75% | *L. japonicus* | Responsive to arsenic transportation in crops plant | (5) |
| BrNIP7;1 | [Bra020777](javascript:modalDialog('multiSearch.php?gene=Bra020777','select%20database',390,200)) | ABY19373 | [Major intrinsic protein NIP5;1](http://blast.ncbi.nlm.nih.gov/Blast.cgi#alnHdr_162568623) | 77% | 4e-48 | 46% | [*L. japonicus*](http://blast.ncbi.nlm.nih.gov/Blast.cgi#alnHdr_162568623) | Responsive to arsenic transportation in crops plant | (5) |
|  |  | [XP007034632](http://www.ncbi.nlm.nih.gov/protein/590657675?report=genbank&log$=prottop&blast_rank=23&RID=BYRTDV2T016) | [Aquaporin NIP1;1,](http://blast.ncbi.nlm.nih.gov/Blast.cgi#alnHdr_590657675) | 90% | 3e-90 | 57% | *T. cacao* | Responsive to Pod colour development | (3) |
| BrTIP1;1 | [Bra017222](javascript:modalDialog('multiSearch.php?gene=Bra017222','select%20database',390,200)) | [ACI95283](http://www.ncbi.nlm.nih.gov/protein/15228041?report=genbank&log$=prottop&blast_rank=12&RID=BZ33P4XH01R) | [Tonoplast intrinsic protein](http://blast.ncbi.nlm.nih.gov/Blast.cgi#alnHdr_227434194) | 100% | 1e-169 | 98% | [*Sinapis arvensis*](http://blast.ncbi.nlm.nih.gov/Blast.cgi#alnHdr_209892837) | ------------------ | Unpublished |
|  |  | [ACP28878](http://www.ncbi.nlm.nih.gov/protein/227434194?report=genbank&log$=prottop&blast_rank=14&RID=BZ33P4XH01R) | [Tonoplast intrinsic protein](http://blast.ncbi.nlm.nih.gov/Blast.cgi#alnHdr_227434194) | 100% | 3e-157 | 90% | *G. hirsutum* | Responsive to cell freezing-tolerance | (6) |
| BrTIP1;2a | [Bra025210](javascript:modalDialog('multiSearch.php?gene=Bra025210','select%20database',390,200)) | AAD39372 | [tonoplast intrinsic protein](http://blast.ncbi.nlm.nih.gov/Blast.cgi#alnHdr_5081419) | 100% | 7e-171 | 98% | [*B. napus*](http://blast.ncbi.nlm.nih.gov/Blast.cgi#alnHdr_5081419) | Responsive to water transportation and cell growth associated with radicle protrusion. | [(7)](http://www.ncbi.nlm.nih.gov/pubmed?term=Gao%20YP%5BAuthor%5D&cauthor=true&cauthor_uid=10480387) |
|  |  | [AAB62692](http://www.ncbi.nlm.nih.gov/protein/2246621?report=genbank&log$=prottop&blast_rank=17&RID=BYCAZ4Z2013) | [Salt-stress induced tonoplast intrinsic protein](http://blast.ncbi.nlm.nih.gov/Blast.cgi#alnHdr_2246621) | 100% | 2e-152 | 84% | *A. thaliana* | Responsive to salt stress and ABA treatment | (8) |
| BrTIP1;2b | [Bra032937](javascript:modalDialog('multiSearch.php?gene=Bra032937','select%20database',390,200)) | AAD39372 | [Tonoplast intrinsic protein](http://blast.ncbi.nlm.nih.gov/Blast.cgi#alnHdr_5081419) | 100% | 6e-165 | 94% | [*B. napus*](http://blast.ncbi.nlm.nih.gov/Blast.cgi#alnHdr_5081419) | Responsive to water transportation and cell growth associated with radicle protrusion. | [(7)](http://www.ncbi.nlm.nih.gov/pubmed?term=Gao%20YP%5BAuthor%5D&cauthor=true&cauthor_uid=10480387) |
|  |  | [AAB62692](http://www.ncbi.nlm.nih.gov/protein/2246621?report=genbank&log$=prottop&blast_rank=17&RID=BTU2PZ1A01R) | [Salt-stress induced tonoplast intrinsic protein](http://blast.ncbi.nlm.nih.gov/Blast.cgi#alnHdr_2246621) | 100% | 4e-155 | 85% | *A. thaliana* | Responsive to high salt condition and ABA treatments | (8) |
| BrTIP1;3 | [Bra037415](javascript:modalDialog('multiSearch.php?gene=Bra037415','select%20database',390,200)) | [XP002872891](http://www.ncbi.nlm.nih.gov/protein/297810015?report=genbank&log$=prottop&blast_rank=4&RID=KCFYH2T9013) | [Aquaporin TIP1;3](http://blast.ncbi.nlm.nih.gov/Blast.cgi#alnHdr_15234189) | 100% | 5e-169 | 97% | *A.* [*lyrata subsp. lyrata*](http://blast.ncbi.nlm.nih.gov/Blast.cgi#alnHdr_297839189) |  | Unpublished |
|  |  | [XP007009482](http://www.ncbi.nlm.nih.gov/protein/590563830?report=genbank&log$=prottop&blast_rank=16&RID=BT7E5DXB013) | [Tonoplast intrinsic protein 1,3](http://blast.ncbi.nlm.nih.gov/Blast.cgi#alnHdr_590563830) | 100% | 1e-145 | 83% | *T. cacao* | Responsive to pod colour | (3) |
| BrTIP2;1a | [Bra027181](javascript:modalDialog('multiSearch.php?gene=Bra027181','select%20database',390,200)) | [AAL38357](http://www.ncbi.nlm.nih.gov/protein/17473872?report=genbank&log$=prottop&blast_rank=11&RID=KCFR4F6Z013) | Delta-tonoplast intrinsic protein | 100% | 2e-160 | 96% | *A. thaliana* |  | Unpublished |
|  |  | [ADE34289.](http://www.ncbi.nlm.nih.gov/protein/292653543?report=genbank&log$=prottop&blast_rank=25&RID=BW52D4BJ013) | [Aquaporin TIP2;3](http://blast.ncbi.nlm.nih.gov/Blast.cgi#alnHdr_292653543) | 100% | 2e-145 | 85% | *G. hirsutum* | Responsive to water balance and nutrient uptake in cotton | (9) |
| BrTIP2;1b | [Bra001626](javascript:modalDialog('multiSearch.php?gene=Bra001626','select%20database',390,200)) | [AAL38357](http://www.ncbi.nlm.nih.gov/protein/17473872?report=genbank&log$=prottop&blast_rank=9&RID=KCEXEBKS013) | [Alpha-tonoplast intrinsic protein](http://blast.ncbi.nlm.nih.gov/Blast.cgi#alnHdr_297839189) | 100% | 4e-158 | 94% | *A. thaliana* |  | Unpublished |
|  |  | [AFH36343](http://www.ncbi.nlm.nih.gov/protein/383479042?report=genbank&log$=prottop&blast_rank=46&RID=C3AZ3S9B015) | [Aquaporin TIP2;1](http://blast.ncbi.nlm.nih.gov/Blast.cgi#alnHdr_383479042) | 100% | 5e-141 | 83% | *Quercus petraea* | Responsive to root development | (10) |
| BrTIP2;1c | [Bra021171](javascript:modalDialog('multiSearch.php?gene=Bra021171','select%20database',390,200)) | NP188245 | [Aquaporin TIP2-1](http://blast.ncbi.nlm.nih.gov/Blast.cgi#alnHdr_15233320) | 97% | 1e-131 | 96% | *A. thaliana* |  | [Salanoubat](http://www.ncbi.nlm.nih.gov/pubmed?term=Salanoubat%20M%5BAuthor%5D&cauthor=true&cauthor_uid=11130713) et al., 2000 |
|  |  | [AFH36343](http://www.ncbi.nlm.nih.gov/protein/383479042?report=genbank&log$=prottop&blast_rank=30&RID=BYR1226N013) | [Aquaporin TIP2;1](http://blast.ncbi.nlm.nih.gov/Blast.cgi#alnHdr_383479042) | 98% | 3e-117 | 85% | *Q. petraea* | Responsive to root development | (10) |
| BrTIP2;2 | [Bra026245](javascript:modalDialog('multiSearch.php?gene=Bra026245','select%20database',390,200)) | [NP199556](http://www.ncbi.nlm.nih.gov/protein/15238100?report=genbank&log$=prottop&blast_rank=8&RID=DHTS917Z014) | [Aquaporin TIP2-3](http://blast.ncbi.nlm.nih.gov/Blast.cgi#alnHdr_15238100) | 98% | 2e-154 | 91% | *A. thaliana* |  | Unpublished |
|  |  | XP007039804 | \|  \|  \| \| --- \| --- \|   Tonoplast intransic protein2;3 | 99% | 1e-138 | 86% | *T. cacao* | Responsive to pod colour | (3) |
| BrTIP2;3a | [Bra024943](javascript:modalDialog('multiSearch.php?gene=Bra024943','select%20database',390,200)) | [NP199556](http://www.ncbi.nlm.nih.gov/protein/15238100?report=genbank&log$=prottop&blast_rank=4&RID=DSVNMM6H01R) | [Aquaporin TIP2;3](http://blast.ncbi.nlm.nih.gov/Blast.cgi#alnHdr_15238100) | 100% | 1e-156 | 91% | *A. thaliana* |  | Unpublished |
|  |  | [CAA65187](http://www.ncbi.nlm.nih.gov/protein/1212921?report=genbank&log$=prottop&blast_rank=33&RID=BYDDMY72013) | Aquaporin | 97% | 2e-133 | 80% | *Helianthus annuus* | Responsive to drought | (11) |
| BrTIP2;3b | [Bra022131](javascript:modalDialog('multiSearch.php?gene=Bra022131','select%20database',390,200)) | [NP199556](http://www.ncbi.nlm.nih.gov/protein/15238100?report=genbank&log$=prottop&blast_rank=2&RID=DSZG4C7E01R) | [Aquaporin TIP2;3](http://blast.ncbi.nlm.nih.gov/Blast.cgi#alnHdr_15238100) | 98% | 2e-128 | 94% | *A. thaliana* |  | Unpublished |
|  |  | NP001274703 | [TIP protein](http://blast.ncbi.nlm.nih.gov/Blast.cgi#alnHdr_567757473) | 97% | 1e-110 | 84% | [*S. lycopersicum*](http://blast.ncbi.nlm.nih.gov/Blast.cgi#alnHdr_560891773) | Responsive to tissue-specific and development-specific expression; water and solute transport in leaves and during fruit development. | (2) |
| BrTIP3;1a | [Bra008079](javascript:modalDialog('multiSearch.php?gene=Bra008079','select%20database',390,200)) | [XP002887476](http://www.ncbi.nlm.nih.gov/protein/297839189?report=genbank&log$=prottop&blast_rank=4&RID=KCEM55P4013) | [Alpha-tonoplast intrinsic protein](http://blast.ncbi.nlm.nih.gov/Blast.cgi#alnHdr_297839189) | 100% | 4e-168 | 94% | *A.* [*lyrata subsp. lyrata*](http://blast.ncbi.nlm.nih.gov/Blast.cgi#alnHdr_297839189) |  | Unpublished |
|  |  | [BAO18638](http://www.ncbi.nlm.nih.gov/protein/560891759?report=genbank&log$=prottop&blast_rank=77&RID=BZ83BYPV013) | [Tonoplast intrinsic protein 3;1](http://blast.ncbi.nlm.nih.gov/Blast.cgi#alnHdr_560891759) | 90% | 3e-113 | 71% | *S. lycopersicum* | Responsive to tissue-specific and development-specific expression; water and solute transport in leaves and during fruit development. | (2) |
| BrTIP3;1b | [Bra016014](javascript:modalDialog('multiSearch.php?gene=Bra016014','select%20database',390,200)) | [XP002887476](http://www.ncbi.nlm.nih.gov/protein/297839189?report=genbank&log$=prottop&blast_rank=10&RID=KCDRWX3D013) | [Alpha-tonoplast intrinsic protein](http://blast.ncbi.nlm.nih.gov/Blast.cgi#alnHdr_297839189) | 100% | 1e-160 | 90% | *A.* [*lyrata subsp. lyrata*](http://blast.ncbi.nlm.nih.gov/Blast.cgi#alnHdr_297839189) |  | Unpublished |
|  |  | BAO18638 | [Tonoplast intrinsic protein 3;1](http://blast.ncbi.nlm.nih.gov/Blast.cgi#alnHdr_560891759) | 90% | 9e-114 | 70% | *S. lycopersicum* | Responsive to tissue-specific and development-specific expression; water and solute transport in leaves and during fruit development. | (2) |
| BrTIP3;2a | [Bra025947](javascript:modalDialog('multiSearch.php?gene=Bra025947','select%20database',390,200)) | [XP002890250](http://www.ncbi.nlm.nih.gov/protein/297844738?report=genbank&log$=prottop&blast_rank=8&RID=KCD354VG016) | [Beta-tonoplast intrinsic protein](http://blast.ncbi.nlm.nih.gov/Blast.cgi#alnHdr_15220848) | 100% | 8e-163 | 91% | *A.* [*lyrata subsp. lyrata*](http://blast.ncbi.nlm.nih.gov/Blast.cgi#alnHdr_297844738) |  | Unpublished |
|  |  | NP001105045 | Aquaporin TIP3;2 | 98% | 1e-112 | 62% | *Z. mays* | Responsive to tissue specific expression of the genes | (4) |
| BrTIP3;2b | [Bra031005](javascript:modalDialog('multiSearch.php?gene=Bra031005','select%20database',390,200)) | XP007099759 | Beta-tonoplast intrinsic prote in | 97% | 2e-125 | 74% | [*T. cacao*](http://blast.ncbi.nlm.nih.gov/Blast.cgi#alnHdr_508775618) | Responsive to Pod colour development | (3) |
|  |  | \|  \| \| --- \|   NP001241721 | [Aquaporin TIP3;2](http://blast.ncbi.nlm.nih.gov/Blast.cgi#alnHdr_162463618) | 98% | 5e-112 | 62% | *Z. mays* | Responsive to tissue specific expression of the genes | (4) |
| BrTIP4;1 | [Bra034271](javascript:modalDialog('multiSearch.php?gene=Bra034271','select%20database',390,200)) | [XP002878889](http://www.ncbi.nlm.nih.gov/protein/297822013?report=genbank&log$=prottop&blast_rank=8&RID=KGFZDCT2014) | [TIP4;1](http://blast.ncbi.nlm.nih.gov/Blast.cgi#alnHdr_15225195) | 100% | 8e-153 | 93% | *A.* [*lyrata subsp. lyrata*](http://blast.ncbi.nlm.nih.gov/Blast.cgi#alnHdr_297844738) |  | Unpublished |
|  |  | DAA33872 | [TPA: aquaporin TIP4;1](http://blast.ncbi.nlm.nih.gov/Blast.cgi#alnHdr_300793618) | 97% | 3e-128 | 81% | *G. hirsutum* | Responsive to water balance and nutrient uptake in cotton | (9) |
| BrTIP5;1 | [Bra018148](javascript:modalDialog('multiSearch.php?gene=Bra018148','select%20database',390,200)) | [EOY22874](http://www.ncbi.nlm.nih.gov/protein/508775618?report=genbank&log$=prottop&blast_rank=5&RID=DT2F1N9J01R) | [Tonoplast intrinsic protein 5.1](http://blast.ncbi.nlm.nih.gov/Blast.cgi#alnHdr_566146415) | 98% | 3e-109 | 68% | [*T. cacao*](http://blast.ncbi.nlm.nih.gov/Blast.cgi#alnHdr_508775618) | Responsive to Pod colour development | (3) |
|  |  | [NP001105036](http://www.ncbi.nlm.nih.gov/protein/162461657?report=genbank&log$=prottop&blast_rank=70&RID=BZ25D4ZU016) | [Aquaporin TIP5;1](http://blast.ncbi.nlm.nih.gov/Blast.cgi#alnHdr_162461657) | 86% | 1e-63 | 78% | *Z. mays* | Responsive to tissue specific expression | (4) |
| BrPIP1;1a | [Bra007603](javascript:modalDialog('multiSearch.php?gene=Bra007603','select%20database',390,200)) | [BAA32777](http://www.ncbi.nlm.nih.gov/protein/3551131?report=genbank&log$=prottop&blast_rank=1&RID=DTCB99RH01R) | [Plasma membrane aquaporin](http://blast.ncbi.nlm.nih.gov/Blast.cgi#alnHdr_3551131) | 100% | 0 | 99% | [*R. sativus*](http://blast.ncbi.nlm.nih.gov/Blast.cgi#alnHdr_3551131) | Responsive to organ- and tissue-specific manner | [(12)](http://www.ncbi.nlm.nih.gov/pubmed?term=Suga%20S%5BAuthor%5D&cauthor=true&cauthor_uid=11216851) |
|  |  | KC969669 | Aquaporin MaPIP1;1 | 100 |  | 75% | Musa acuminata | Responsive to drought and salt stress | (13) |
| BrPIP1;1b | [Bra014437](javascript:modalDialog('multiSearch.php?gene=Bra014437','select%20database',390,200)) | [BAA32777](http://www.ncbi.nlm.nih.gov/protein/3551131?report=genbank&log$=prottop&blast_rank=6&RID=KH35334J014) | [Plasma membrane aquaporin (PAQ1)](http://blast.ncbi.nlm.nih.gov/Blast.cgi#alnHdr_3551131) | 100% | 0 | 98% | [*R. sativus*](http://blast.ncbi.nlm.nih.gov/Blast.cgi#alnHdr_3551131) | Regulated in certain tissues at the translational level and by the rate of protein turnover. | [(12)](http://www.ncbi.nlm.nih.gov/pubmed?term=Suga%20S%5BAuthor%5D&cauthor=true&cauthor_uid=11216851) |
|  |  | KC969669 | Aquaporin MaPIP1;1 | 100 | 1e-65 | 74% | M. acuminata | Responsive to drought and salt stress | (13) |
| BrPIP1;2a | [Bra039301](javascript:modalDialog('multiSearch.php?gene=Bra039301','select%20database',390,200)) | CAA64896 | [Transmembrane channel protein](http://blast.ncbi.nlm.nih.gov/Blast.cgi#alnHdr_1199503) | 100% | 1e-174 | 88% | *B. oleracea* | Responsive to drought inducibility and constituents of the pollen coat | [(14)](http://www.ncbi.nlm.nih.gov/pubmed?term=Ruiter%20RK%5BAuthor%5D&cauthor=true&cauthor_uid=9177322) |
|  |  | [AAG23180](http://www.ncbi.nlm.nih.gov/protein/10799095?report=genbank&log$=prottop&blast_rank=5&RID=BT5PMESM013) | [Aquaporin PIP1b2](http://blast.ncbi.nlm.nih.gov/Blast.cgi#alnHdr_10799095) | 100% | 2e-173 | 87% | *B. oleracea* | Responsive to water flow between the pollen and stigma papillae | (15) |
| BrPIP1;2b | [Bra004950](javascript:modalDialog('multiSearch.php?gene=Bra004950','select%20database',390,200)) | [AAG23179](http://www.ncbi.nlm.nih.gov/protein/10799093?report=genbank&log$=prottop&blast_rank=7&RID=PS052DPA015) | Aquaporin PIP1b2 | 100% | 00 | 98% | *B. oleracea* | Responsive to water flow between the pollen and stigma papillae | (15) |
|  |  | KC969669 | Aquaporin MaPIP1;1 | 100 | 2e-107 | 75% | M. acuminata | Responsive to drought and salt stress | (13) |
| BrPIP1;3a | [Bra032644](javascript:modalDialog('multiSearch.php?gene=Bra032644','select%20database',390,200)) | [AAG23180](http://www.ncbi.nlm.nih.gov/protein/10799093?report=genbank&log$=prottop&blast_rank=7&RID=PS052DPA015) | [Aquaporin PIP1b2](http://blast.ncbi.nlm.nih.gov/Blast.cgi#alnHdr_10799095) | 100% | 00 | 95% | *B. oleracea* | Responsive to water flow between the pollen and stigma papillae | (15) |
|  |  | [AEA40491](http://www.ncbi.nlm.nih.gov/protein/327242161?report=genbank&log$=prottop&blast_rank=51&RID=BTW8AZF801R) | [Aquaporin PIP1-3](http://blast.ncbi.nlm.nih.gov/Blast.cgi#alnHdr_327242161) | 97% | 0.0 | 96% | *B. oleracea var. italica* | Responsive to Salinity | (16) |
| BrPIP1;3b | [Bra033248](javascript:modalDialog('multiSearch.php?gene=Bra033248','select%20database',390,200)) | [AAL32688](http://www.ncbi.nlm.nih.gov/protein/17065068?report=genbank&log$=prottop&blast_rank=17&RID=KC3PGZ0N016) | [Plasma membrane intrinsic protein 1C](http://blast.ncbi.nlm.nih.gov/Blast.cgi#alnHdr_17065068) | 100% | 0 | 97% | *A. thaliana* |  | Unpublished |
|  |  | KC969669 | Aquaporin MaPIP1;1 | 100 | 13-65 | 75% | M. acuminata | Responsive to drought and salt stress | (13) |
| BrPIP1;4 | [Bra000974](javascript:modalDialog('multiSearch.php?gene=Bra000974','select%20database',390,200)) | [NP567178](http://www.ncbi.nlm.nih.gov/protein/18411332?report=genbank&log$=prottop&blast_rank=4&RID=DTKPHJHM01R) | [Plasma membrane intrinsic protein 1;4](http://blast.ncbi.nlm.nih.gov/Blast.cgi#alnHdr_18411332) | 99% | 0 | 98% | *A. thaliana* |  | Unpublished |
|  |  | KC969669 | Aquaporin MaPIP1;1 | 100 | 1e-65 | 75% | M. acuminata | Responsive to drought and salt stress | (13) |
| BrPIP1;5 | [Bra019307](javascript:modalDialog('multiSearch.php?gene=Bra019307','select%20database',390,200)) | [XP002869782](http://www.ncbi.nlm.nih.gov/protein/297803796?report=genbank&log$=prottop&blast_rank=8&RID=KC13R0W0013) | PIP1_5/PIP1D | 100% | 0 | 97% | *A.*  *lyrata subsp.lyrata* |  | Unpublished |
|  |  | KC969669 | Aquaporin MaPIP1;1 | 100 | 1e-107 | 75% | M. acuminata | Responsive to drought and salt stress | (13) |
| BrPIP2;1 | [Bra006997](javascript:modalDialog('multiSearch.php?gene=Bra006997','select%20database',390,200)) | [AAD39373](http://www.ncbi.nlm.nih.gov/protein/5081421?report=genbank&log$=prottop&blast_rank=1&RID=DTESD0E301R) | [Plasma membrane intrinsic protein 1](http://blast.ncbi.nlm.nih.gov/Blast.cgi#alnHdr_5081421) | 100% | 0 | 100% | [*B. napus*](http://blast.ncbi.nlm.nih.gov/Blast.cgi#alnHdr_5081419) | Responsive to water transportation | (7) |
|  |  | \|  \|  \| \| --- \| --- \|   ABK60195 | PIP2 protein | 99% | 4e-175 | 86% | *G. hirsutum* | Responsive to root development and in stresses (NaCl, cold, PEG). | (6) |
| BrPIP2;2a | [Bra023102](javascript:modalDialog('multiSearch.php?gene=Bra023102','select%20database',390,200)) | [BAA92261](http://www.ncbi.nlm.nih.gov/protein/7209562?report=genbank&log$=prottop&blast_rank=2&RID=KH4C0069015) | [Plasma membrane aquaporin 2C](http://blast.ncbi.nlm.nih.gov/Blast.cgi#alnHdr_3551131) | 100% | 0 | 99% | [*R. sativus*](http://blast.ncbi.nlm.nih.gov/Blast.cgi#alnHdr_3551131) | Regulated in certain tissues at the translational level and by the rate of protein turnover. | [(12)](http://www.ncbi.nlm.nih.gov/pubmed?term=Suga%20S%5BAuthor%5D&cauthor=true&cauthor_uid=11216851) |
|  |  | [AAO39008](http://www.ncbi.nlm.nih.gov/protein/28395420?report=genbank&log$=prottop&blast_rank=52&RID=BYEX2MA0016) | [Plasma intrinsic protein 2,2](http://blast.ncbi.nlm.nih.gov/Blast.cgi#alnHdr_28395420) | 100% | 7e-173 | 83% | *Juglans regia* | Responsive to water transport between xylem parenchyma cells and embolized vessels | (17) |
| BrPIP2;2b | [Bra005215](javascript:modalDialog('multiSearch.php?gene=Bra005215','select%20database',390,200)) | BAA92260 | Plasma membrane aquaporin 2b | 99% | 2e-156 | 82% | *R. sativus* | Regulated in an organ- and tissue-specific manner, water transport in plant body during growth and development. | [(12)](http://www.ncbi.nlm.nih.gov/pubmed?term=Suga%20S%5BAuthor%5D&cauthor=true&cauthor_uid=11216851) |
|  |  | \|  \|  \| \| --- \| --- \|   BK60195 | PIP2 protein | 98% | 4e-131 | 69% | *G. hirsutum* | Responsive to root development and in stresses (NaCl, cold, PEG). | (6) |
| BrPIP2;3a | [Bra023103](javascript:modalDialog('multiSearch.php?gene=Bra023103','select%20database',390,200)) | BAA92261.1 | Plasma membrane aquaporin 2c | 99% | 0 | 97% | [*R. sativus*](http://blast.ncbi.nlm.nih.gov/Blast.cgi#alnHdr_7209562) | Regulated in an organ- and tissue-specific manner | [(12)](http://www.ncbi.nlm.nih.gov/pubmed?term=Suga%20S%5BAuthor%5D&cauthor=true&cauthor_uid=11216851) |
|  |  | AEQ29857 | Aquaporin PIP2 | 99% | 1e-172 | 84% | *Malus prunifolia* | Responsive to high salinity conditions | (18) |
| BrPIP2;3b | [Bra005216](javascript:modalDialog('multiSearch.php?gene=Bra005216','select%20database',390,200)) | BAA92260 | Plasma membrane aquaporin 2b | 99% | 1e-155 | 99% | *R. sativus* | Regulated in an organ- and tissue-specific manner, water transport in plant body during growth and development | [(12)](http://www.ncbi.nlm.nih.gov/pubmed?term=Suga%20S%5BAuthor%5D&cauthor=true&cauthor_uid=11216851) |
|  |  | \|  \|  \| \| --- \| --- \|   ABK60195 | PIP2 protein | 94% | 4e-137 | 87% | *G. hirsutum* | Responsive to root development and in stresses (NaCl, cold, PEG). | (6) |
| BrPIP2;4a | [Bra020238](javascript:modalDialog('multiSearch.php?gene=Bra020238','select%20database',390,200)) | ACU00103 | Plasma membrane intrinsic protein2;1 | 100% | 2e-172 | 82% | [*P. trichocarpa x P. deltoides*](http://blast.ncbi.nlm.nih.gov/Blast.cgi#alnHdr_255045893) |  | Unpublished |
|  |  | [AAO39008](http://www.ncbi.nlm.nih.gov/protein/28395420?report=genbank&log$=prottop&blast_rank=65&RID=BYSF0PC501R) | [Plasma intrinsic protein 2,2](http://blast.ncbi.nlm.nih.gov/Blast.cgi#alnHdr_28395420) | 100% | 5e-170 | 81% | [*Juglans regia*](http://blast.ncbi.nlm.nih.gov/Blast.cgi#alnHdr_28395420) | Responsive to water transport between xylem parenchyma cells and embolized vessels | (17) |
| BrPIP2;4b | [Bra006650](javascript:modalDialog('multiSearch.php?gene=Bra006650','select%20database',390,200)) | [BAA32778](http://www.ncbi.nlm.nih.gov/protein/3551133?report=genbank&log$=prottop&blast_rank=22&RID=KHCSBX5201R) | [Plasma membrane aquaporin (PAQ2)](http://blast.ncbi.nlm.nih.gov/Blast.cgi#alnHdr_3551133) | 100% | 4e-175 | 84% | *R. sativus* | Regulated in an organ- and tissue-specific manner | [(12)](http://www.ncbi.nlm.nih.gov/pubmed?term=Suga%20S%5BAuthor%5D&cauthor=true&cauthor_uid=11216851) |
|  |  | AAV69744 | Aquaporin | 99% | 6e-172 | 82% | *Vitis vinifera* | Responsive to water transport and drought stress | (19) |
| BrPIP2;4c | [Bra002462](javascript:modalDialog('multiSearch.php?gene=Bra002462','select%20database',390,200)) | [NP200874](http://www.ncbi.nlm.nih.gov/protein/15239397?report=genbank&log$=prottop&blast_rank=15&RID=KC00WRG3016) | Aquaporin PIP2;4 | 99% | 1e-163 | 83% | *A. thaliana* |  | [Unpublished](http://www.ncbi.nlm.nih.gov/pubmed?term=Tuskan%20GA%5BAuthor%5D&cauthor=true&cauthor_uid=16973872) |
|  |  | [AAV69744](http://www.ncbi.nlm.nih.gov/protein/55982653?report=genbank&log$=prottop&blast_rank=35&RID=C38T62Y4015) | [Aquaporin PIP2](http://blast.ncbi.nlm.nih.gov/Blast.cgi#alnHdr_350543336) | 100% | 9e-151 | 77% | [*Malus prunifolia*](http://blast.ncbi.nlm.nih.gov/Blast.cgi#alnHdr_350543336) | Responsive to water transport and drought stress | (18) |
| BrPIP2;5a | [Bra007100](javascript:modalDialog('multiSearch.php?gene=Bra007100','select%20database',390,200)) | [XP002876271](http://www.ncbi.nlm.nih.gov/protein/297816776?report=genbank&log$=prottop&blast_rank=3&RID=DTE66B3T01R) | [PIP2_5/PIP2D](http://blast.ncbi.nlm.nih.gov/Blast.cgi#alnHdr_297816776) | 100% | 0 | 94% | [*A. lyrata subsp. lyrata*](http://blast.ncbi.nlm.nih.gov/Blast.cgi#alnHdr_297820476) |  | Unpublished |
|  |  | [AAV69744](http://www.ncbi.nlm.nih.gov/protein/55982653?report=genbank&log$=prottop&blast_rank=47&RID=C1SMRB7G016) | [Aquaporin](http://blast.ncbi.nlm.nih.gov/Blast.cgi#alnHdr_55982653) | 100% | 9e-170 | 83% | [*V. vinifera*](http://blast.ncbi.nlm.nih.gov/Blast.cgi#alnHdr_55982653) | Responsive to water transport and drought stress | (19) |
| BrPIP2;5b | [Bra003196](javascript:modalDialog('multiSearch.php?gene=Bra003196','select%20database',390,200)) | [XP002876271](http://www.ncbi.nlm.nih.gov/protein/297816776?report=genbank&log$=prottop&blast_rank=4&RID=DTHTWTSY01R) | [PIP2;5/PIP2D](http://blast.ncbi.nlm.nih.gov/Blast.cgi#alnHdr_297816776) | 100% | 0 | 92% | [*A. lyrata subsp. lyrata*](http://blast.ncbi.nlm.nih.gov/Blast.cgi#alnHdr_297820476) |  | Unpublished |
|  |  | [AAV69744](http://www.ncbi.nlm.nih.gov/protein/55982653?report=genbank&log$=prottop&blast_rank=45&RID=C3873XAD015) | [Aquaporin](http://blast.ncbi.nlm.nih.gov/Blast.cgi#alnHdr_55982653) | 100% | 5e-168 | 82% | [*V. vinifera*](http://blast.ncbi.nlm.nih.gov/Blast.cgi#alnHdr_55982653) | Responsive to water transport and drought stress | (19) |
| BrPIP2;6 | [Bra000111](javascript:modalDialog('multiSearch.php?gene=Bra000111','select%20database',390,200)) | [AEQ29857](http://www.ncbi.nlm.nih.gov/protein/350543336?report=genbank&log$=prottop&blast_rank=50&RID=C3BYDFZX014) | [Aquaporin PIP2](http://blast.ncbi.nlm.nih.gov/Blast.cgi#alnHdr_543177273) | 97% | 3e-160 | 78% | [*M. prunifolia*](http://blast.ncbi.nlm.nih.gov/Blast.cgi#alnHdr_350543336) | Responsive to salt stress | (18) |
|  |  | [AAV69744](http://www.ncbi.nlm.nih.gov/protein/55982653?report=genbank&log$=prottop&blast_rank=49&RID=C3BYDFZX014) | [Aquaporin](http://blast.ncbi.nlm.nih.gov/Blast.cgi#alnHdr_55982653) | 97% | 3e-160 | 79% | [*V. vinifera*](http://blast.ncbi.nlm.nih.gov/Blast.cgi#alnHdr_55982653) | Responsive to water transport and drought stress | (19) |
| BrPIP2;7a | [Bra034675](javascript:modalDialog('multiSearch.php?gene=Bra034675','select%20database',390,200)) | [ACB42441](http://www.ncbi.nlm.nih.gov/protein/171190270?report=genbank&log$=prottop&blast_rank=25&RID=KC0PHYHW016) | Aquaporin PIP2;4 | 100% | 6e-179 | 89% | *G. hirsutum* | Regulate their water channel activities and are required for fibre development | (20) |
|  |  | [AFB83067](http://www.ncbi.nlm.nih.gov/protein/378408543?report=genbank&log$=prottop&blast_rank=29&RID=BTEEHR4301R) | [Plasma intrinsic protein PIP2.1](http://blast.ncbi.nlm.nih.gov/Blast.cgi#alnHdr_378408543) | 100% | 2e-178 | 89% | *H. almeriense* | Responsive to drought stress (morphophysiological adaptation of this symbiosis to drought conditions) | (21) |
| BrPIP2;7b | [Bra011585](javascript:modalDialog('multiSearch.php?gene=Bra011585','select%20database',390,200)) | [NP001267957](http://www.ncbi.nlm.nih.gov/protein/526118191?report=genbank&log$=prottop&blast_rank=40&RID=PSHBJFGU014) | [Aquaporin](http://blast.ncbi.nlm.nih.gov/Blast.cgi#alnHdr_11119335) PIP 2;2 | 100% | 0 | 88% | Vitis vinifera | Water transport and drought stress responses | (19) |
|  |  | [AFB83067](http://www.ncbi.nlm.nih.gov/protein/378408543?report=genbank&log$=prottop&blast_rank=38&RID=PSHBJFGU014) | [Plasma intrinsic protein PIP2.1](http://blast.ncbi.nlm.nih.gov/Blast.cgi#alnHdr_378408543) | 100% | 0.0 | 89% | *H. almeriense* | Responsive to drought conditions | (21) |
| BrPIP2;7c | [Bra017697](javascript:modalDialog('multiSearch.php?gene=Bra017697','select%20database',390,200)) | [AAG30607](http://www.ncbi.nlm.nih.gov/protein/11119335?report=genbank&log$=prottop&blast_rank=2&RID=DT2TT7K101R) | [aquaporin](http://blast.ncbi.nlm.nih.gov/Blast.cgi#alnHdr_11119335) | 100% | 0 | 95% | [*B. oleracea*](http://blast.ncbi.nlm.nih.gov/Blast.cgi#alnHdr_11119335) |  | Unpublished |
|  |  | [AAB65787](http://www.ncbi.nlm.nih.gov/protein/2306917?report=genbank&log$=prottop&blast_rank=14&RID=BZ2RHJS8013) | [Plasma membrane intrinsic protein](http://blast.ncbi.nlm.nih.gov/Blast.cgi#alnHdr_2306917) | 100% | 0.0 | 95% | *A. thaliana* | Responsive to salt stress and ABA treatment | (8) |

References:

1. [Salanoubat M](https://www.ncbi.nlm.nih.gov/pubmed/?term=Salanoubat%20M%5BAuthor%5D&cauthor=true&cauthor_uid=11130713), [Lemcke K](https://www.ncbi.nlm.nih.gov/pubmed/?term=Lemcke%20K%5BAuthor%5D&cauthor=true&cauthor_uid=11130713), [Rieger M](https://www.ncbi.nlm.nih.gov/pubmed/?term=Rieger%20M%5BAuthor%5D&cauthor=true&cauthor_uid=11130713) et al., Sequence and analysis of chromosome 3 of the plant Arabidopsis thaliana. [Nature.](https://www.ncbi.nlm.nih.gov/pubmed/11130713) 2000; 408(6814):820-2.
2. [Reuscher S](http://www.ncbi.nlm.nih.gov/pubmed/?term=Reuscher%20S%5BAuthor%5D&cauthor=true&cauthor_uid=24260152), [Akiyama M](http://www.ncbi.nlm.nih.gov/pubmed/?term=Akiyama%20M%5BAuthor%5D&cauthor=true&cauthor_uid=24260152), [Mori C](http://www.ncbi.nlm.nih.gov/pubmed/?term=Mori%20C%5BAuthor%5D&cauthor=true&cauthor_uid=24260152), [Aoki K](http://www.ncbi.nlm.nih.gov/pubmed/?term=Aoki%20K%5BAuthor%5D&cauthor=true&cauthor_uid=24260152), [Shibata D](http://www.ncbi.nlm.nih.gov/pubmed/?term=Shibata%20D%5BAuthor%5D&cauthor=true&cauthor_uid=24260152), [Shiratake K](http://www.ncbi.nlm.nih.gov/pubmed/?term=Shiratake%20K%5BAuthor%5D&cauthor=true&cauthor_uid=24260152). Genome-wide identification and expression analysis of aquaporins in tomato. [PLoS One.](http://www.ncbi.nlm.nih.gov/pubmed/24260152) 2013; 8(11):e79052. doi: 10.1371/journal.pone.0079052.
3. [Motamayor JC](http://www.ncbi.nlm.nih.gov/pubmed/?term=Motamayor%20JC%5BAuthor%5D&cauthor=true&cauthor_uid=23731509), [Mockaitis K](http://www.ncbi.nlm.nih.gov/pubmed/?term=Mockaitis%20K%5BAuthor%5D&cauthor=true&cauthor_uid=23731509), [Schmutz J](http://www.ncbi.nlm.nih.gov/pubmed/?term=Schmutz%20J%5BAuthor%5D&cauthor=true&cauthor_uid=23731509), [Haiminen N](http://www.ncbi.nlm.nih.gov/pubmed/?term=Haiminen%20N%5BAuthor%5D&cauthor=true&cauthor_uid=23731509), et al. The genome sequence of the most widely cultivated cacao type and its use to identify candidate genes regulating pod color. [Genome Biol.](http://www.ncbi.nlm.nih.gov/pubmed/23731509) 2013; 14(6):r53. doi: 10.1186/gb-2013-14-6-r53.
4. [Chaumont F](http://www.ncbi.nlm.nih.gov/pubmed/?term=Chaumont%20F%5BAuthor%5D&cauthor=true&cauthor_uid=11244102), [Barrieu F](http://www.ncbi.nlm.nih.gov/pubmed/?term=Barrieu%20F%5BAuthor%5D&cauthor=true&cauthor_uid=11244102), [Wojcik E](http://www.ncbi.nlm.nih.gov/pubmed/?term=Wojcik%20E%5BAuthor%5D&cauthor=true&cauthor_uid=11244102), [Chrispeels MJ](http://www.ncbi.nlm.nih.gov/pubmed/?term=Chrispeels%20MJ%5BAuthor%5D&cauthor=true&cauthor_uid=11244102), [Jung R](http://www.ncbi.nlm.nih.gov/pubmed/?term=Jung%20R%5BAuthor%5D&cauthor=true&cauthor_uid=11244102). Aquaporins constitute a large and highly divergent protein family in maize. [Plant Physiol.](http://www.ncbi.nlm.nih.gov/pubmed/11244102) 2001; 125(3):1206-15.
5. [Bienert GP](http://www.ncbi.nlm.nih.gov/pubmed/?term=Bienert%20GP%5BAuthor%5D&cauthor=true&cauthor_uid=18544156), [Thorsen M](http://www.ncbi.nlm.nih.gov/pubmed/?term=Thorsen%20M%5BAuthor%5D&cauthor=true&cauthor_uid=18544156), [Schüssler MD](http://www.ncbi.nlm.nih.gov/pubmed/?term=Sch%C3%BCssler%20MD%5BAuthor%5D&cauthor=true&cauthor_uid=18544156), [Nilsson HR](http://www.ncbi.nlm.nih.gov/pubmed/?term=Nilsson%20HR%5BAuthor%5D&cauthor=true&cauthor_uid=18544156), [Wagner A](http://www.ncbi.nlm.nih.gov/pubmed/?term=Wagner%20A%5BAuthor%5D&cauthor=true&cauthor_uid=18544156), [Tamás MJ](http://www.ncbi.nlm.nih.gov/pubmed/?term=Tam%C3%A1s%20MJ%5BAuthor%5D&cauthor=true&cauthor_uid=18544156), [Jahn TP](http://www.ncbi.nlm.nih.gov/pubmed/?term=Jahn%20TP%5BAuthor%5D&cauthor=true&cauthor_uid=18544156). A subgroup of plant aquaporins facilitate the bi-directional diffusion of As(OH)3 and Sb(OH)3 across membranes. [BMC Biol.](http://www.ncbi.nlm.nih.gov/pubmed/18544156) 2008; 6:26. doi: 10.1186/1741-7007-6-26.
6. [Li DD](http://www.ncbi.nlm.nih.gov/pubmed/?term=Li%20DD%5BAuthor%5D&cauthor=true&cauthor_uid=18956193), [Wu YJ](http://www.ncbi.nlm.nih.gov/pubmed/?term=Wu%20YJ%5BAuthor%5D&cauthor=true&cauthor_uid=18956193), [Ruan XM](http://www.ncbi.nlm.nih.gov/pubmed/?term=Ruan%20XM%5BAuthor%5D&cauthor=true&cauthor_uid=18956193), [Li B](http://www.ncbi.nlm.nih.gov/pubmed/?term=Li%20B%5BAuthor%5D&cauthor=true&cauthor_uid=18956193), [Zhu L](http://www.ncbi.nlm.nih.gov/pubmed/?term=Zhu%20L%5BAuthor%5D&cauthor=true&cauthor_uid=18956193), [Wang H](http://www.ncbi.nlm.nih.gov/pubmed/?term=Wang%20H%5BAuthor%5D&cauthor=true&cauthor_uid=18956193), [Li XB](http://www.ncbi.nlm.nih.gov/pubmed/?term=Li%20XB%5BAuthor%5D&cauthor=true&cauthor_uid=18956193). Expressions of three cotton genes encoding the PIP proteins are regulated in root development and in response to stresses. [Plant Cell Rep.](http://www.ncbi.nlm.nih.gov/pubmed/18956193) 2009; 28(2):291-300. doi: 10.1007/s00299-008-0626-6.
7. 7. [Gao YP](http://www.ncbi.nlm.nih.gov/pubmed/?term=Gao%20YP%5BAuthor%5D&cauthor=true&cauthor_uid=10480387), [Young L](http://www.ncbi.nlm.nih.gov/pubmed/?term=Young%20L%5BAuthor%5D&cauthor=true&cauthor_uid=10480387), [Bonham-Smith P](http://www.ncbi.nlm.nih.gov/pubmed/?term=Bonham-Smith%20P%5BAuthor%5D&cauthor=true&cauthor_uid=10480387), [Gusta LV](http://www.ncbi.nlm.nih.gov/pubmed/?term=Gusta%20LV%5BAuthor%5D&cauthor=true&cauthor_uid=10480387). Characterization and expression of plasma and tonoplast membrane aquaporins in primed seed of Brassica napus during germination under stress conditions. [Plant Mol Biol.](http://www.ncbi.nlm.nih.gov/pubmed/10480387) 1999; 40(4):635-44.
8. [Pih KT](http://www.ncbi.nlm.nih.gov/pubmed/?term=Pih%20KT%5BAuthor%5D&cauthor=true&cauthor_uid=10102577), [Kabilan V](http://www.ncbi.nlm.nih.gov/pubmed/?term=Kabilan%20V%5BAuthor%5D&cauthor=true&cauthor_uid=10102577), [Lim JH](http://www.ncbi.nlm.nih.gov/pubmed/?term=Lim%20JH%5BAuthor%5D&cauthor=true&cauthor_uid=10102577), [Kang SG](http://www.ncbi.nlm.nih.gov/pubmed/?term=Kang%20SG%5BAuthor%5D&cauthor=true&cauthor_uid=10102577), [Piao HL](http://www.ncbi.nlm.nih.gov/pubmed/?term=Piao%20HL%5BAuthor%5D&cauthor=true&cauthor_uid=10102577), [Jin JB](http://www.ncbi.nlm.nih.gov/pubmed/?term=Jin%20JB%5BAuthor%5D&cauthor=true&cauthor_uid=10102577), [Hwang I](http://www.ncbi.nlm.nih.gov/pubmed/?term=Hwang%20I%5BAuthor%5D&cauthor=true&cauthor_uid=10102577). Characterization of two new channel protein genes in Arabidopsis. Mol Cells. 1999; 9(1):84-90.
9. [Park W](http://www.ncbi.nlm.nih.gov/pubmed/?term=Park%20W%5BAuthor%5D&cauthor=true&cauthor_uid=20626869), [Scheffler BE](http://www.ncbi.nlm.nih.gov/pubmed/?term=Scheffler%20BE%5BAuthor%5D&cauthor=true&cauthor_uid=20626869), [Bauer PJ](http://www.ncbi.nlm.nih.gov/pubmed/?term=Bauer%20PJ%5BAuthor%5D&cauthor=true&cauthor_uid=20626869), [Campbell BT](http://www.ncbi.nlm.nih.gov/pubmed/?term=Campbell%20BT%5BAuthor%5D&cauthor=true&cauthor_uid=20626869). Identification of the family of aquaporin genes and their expression in upland cotton (Gossypium hirsutum L.). [BMC Plant Biol.](http://www.ncbi.nlm.nih.gov/pubmed/20626869) 2010; 10:142. doi: 10.1186/1471-2229-10-142.
10. [Rasheed-Depardieu C](http://www.ncbi.nlm.nih.gov/pubmed/?term=Rasheed-Depardieu%20C%5BAuthor%5D&cauthor=true&cauthor_uid=23284785), [Parent C](http://www.ncbi.nlm.nih.gov/pubmed/?term=Parent%20C%5BAuthor%5D&cauthor=true&cauthor_uid=23284785), [Crèvecoeur M](http://www.ncbi.nlm.nih.gov/pubmed/?term=Cr%C3%A8vecoeur%20M%5BAuthor%5D&cauthor=true&cauthor_uid=23284785), [Parelle J](http://www.ncbi.nlm.nih.gov/pubmed/?term=Parelle%20J%5BAuthor%5D&cauthor=true&cauthor_uid=23284785), [Tatin-Froux F](http://www.ncbi.nlm.nih.gov/pubmed/?term=Tatin-Froux%20F%5BAuthor%5D&cauthor=true&cauthor_uid=23284785), [Le Provost G](http://www.ncbi.nlm.nih.gov/pubmed/?term=Le%20Provost%20G%5BAuthor%5D&cauthor=true&cauthor_uid=23284785), [Capelli N](http://www.ncbi.nlm.nih.gov/pubmed/?term=Capelli%20N%5BAuthor%5D&cauthor=true&cauthor_uid=23284785). Identification and expression of nine oak aquaporin genes in the primary root axis of two oak species, Quercus petraea and Quercus robur. [PLoS One.](http://www.ncbi.nlm.nih.gov/pubmed/23284785) 2012; 7(12):e51838.doi: 10.1371/journal.pone.0051838.
11. [Sarda X](http://www.ncbi.nlm.nih.gov/pubmed/?term=Sarda%20X%5BAuthor%5D&cauthor=true&cauthor_uid=10394956), [Tousch D](http://www.ncbi.nlm.nih.gov/pubmed/?term=Tousch%20D%5BAuthor%5D&cauthor=true&cauthor_uid=10394956), [Ferrare K](http://www.ncbi.nlm.nih.gov/pubmed/?term=Ferrare%20K%5BAuthor%5D&cauthor=true&cauthor_uid=10394956), [Cellier F](http://www.ncbi.nlm.nih.gov/pubmed/?term=Cellier%20F%5BAuthor%5D&cauthor=true&cauthor_uid=10394956), [Alcon C](http://www.ncbi.nlm.nih.gov/pubmed/?term=Alcon%20C%5BAuthor%5D&cauthor=true&cauthor_uid=10394956), [Dupuis JM](http://www.ncbi.nlm.nih.gov/pubmed/?term=Dupuis%20JM%5BAuthor%5D&cauthor=true&cauthor_uid=10394956), [Casse F](http://www.ncbi.nlm.nih.gov/pubmed/?term=Casse%20F%5BAuthor%5D&cauthor=true&cauthor_uid=10394956), [Lamaze T](http://www.ncbi.nlm.nih.gov/pubmed/?term=Lamaze%20T%5BAuthor%5D&cauthor=true&cauthor_uid=10394956). (1999) Characterization of closely related delta-TIP genes encoding aquaporins which are differentially expressed in sunflower roots upon water deprivation through exposure to air. [,](http://www.ncbi.nlm.nih.gov/pubmed/10394956) 1999; 40(1):179-91.
12. [Suga S](http://www.ncbi.nlm.nih.gov/pubmed/?term=Suga%20S%5BAuthor%5D&cauthor=true&cauthor_uid=11216851), [Imagawa S](http://www.ncbi.nlm.nih.gov/pubmed/?term=Imagawa%20S%5BAuthor%5D&cauthor=true&cauthor_uid=11216851), [Maeshima M](http://www.ncbi.nlm.nih.gov/pubmed/?term=Maeshima%20M%5BAuthor%5D&cauthor=true&cauthor_uid=11216851). Specificity of the accumulation of mRNAs and proteins of the plasma membrane and tonoplast aquaporins in radish organs. [Planta](http://www.ncbi.nlm.nih.gov/pubmed/11216851), 2001; 212(2):294-304.
13. Xu Y, Hu W, Liu J, Zhang J, Jia C, Miao H, Biyu X, Jin Z. A banana aquaporin gene, MaPIP1; 1, is involved in tolerance to drought and salt stresses. *BMC Plant Biology.* 2014; doi:10.1186/1471-2229-14-59
14. [Ruiter RK](http://www.ncbi.nlm.nih.gov/pubmed/?term=Ruiter%20RK%5BAuthor%5D&cauthor=true&cauthor_uid=9177322), [van Eldik GJ](http://www.ncbi.nlm.nih.gov/pubmed/?term=van%20Eldik%20GJ%5BAuthor%5D&cauthor=true&cauthor_uid=9177322), [van Herpen MM](http://www.ncbi.nlm.nih.gov/pubmed/?term=van%20Herpen%20MM%5BAuthor%5D&cauthor=true&cauthor_uid=9177322), [Schrauwen JA](http://www.ncbi.nlm.nih.gov/pubmed/?term=Schrauwen%20JA%5BAuthor%5D&cauthor=true&cauthor_uid=9177322), [Wullems GJ](http://www.ncbi.nlm.nih.gov/pubmed/?term=Wullems%20GJ%5BAuthor%5D&cauthor=true&cauthor_uid=9177322). Expression in anthers of two genes encoding Brassica oleracea transmembrane channel proteins. [Plant Mol Biol.](http://www.ncbi.nlm.nih.gov/pubmed/9177322) 1997; 34(1):163-8.
15. [Marin-Olivier M](http://www.ncbi.nlm.nih.gov/pubmed/?term=Marin-Olivier%20M%5BAuthor%5D&cauthor=true&cauthor_uid=11069697), [Chevalier T](http://www.ncbi.nlm.nih.gov/pubmed/?term=Chevalier%20T%5BAuthor%5D&cauthor=true&cauthor_uid=11069697), [Fobis-Loisy I](http://www.ncbi.nlm.nih.gov/pubmed/?term=Fobis-Loisy%20I%5BAuthor%5D&cauthor=true&cauthor_uid=11069697), [Dumas C](http://www.ncbi.nlm.nih.gov/pubmed/?term=Dumas%20C%5BAuthor%5D&cauthor=true&cauthor_uid=11069697), [Gaude T](http://www.ncbi.nlm.nih.gov/pubmed/?term=Gaude%20T%5BAuthor%5D&cauthor=true&cauthor_uid=11069697). Aquaporin PIP genes are not expressed in the stigma papillae in Brassica oleracea. [Plant J.](http://www.ncbi.nlm.nih.gov/pubmed/11069697) 2000; 24(2):231-40.
16. [Muries B](http://www.ncbi.nlm.nih.gov/pubmed/?term=Muries%20B%5BAuthor%5D&cauthor=true&cauthor_uid=21321750), [Faize M](http://www.ncbi.nlm.nih.gov/pubmed/?term=Faize%20M%5BAuthor%5D&cauthor=true&cauthor_uid=21321750), [Carvajal M](http://www.ncbi.nlm.nih.gov/pubmed/?term=Carvajal%20M%5BAuthor%5D&cauthor=true&cauthor_uid=21321750), [Martínez-Ballesta Mdel C](http://www.ncbi.nlm.nih.gov/pubmed/?term=Mart%C3%ADnez-Ballesta%20Mdel%20C%5BAuthor%5D&cauthor=true&cauthor_uid=21321750). Identification and differential induction of the expression of aquaporins by salinity in broccoli plants. [Mol Biosyst.](http://www.ncbi.nlm.nih.gov/pubmed/21321750) 2011; 7(4):1322-35. doi: 10.1039/c0mb00285b.
17. [Sakr S](http://www.ncbi.nlm.nih.gov/pubmed/?term=Sakr%20S%5BAuthor%5D&cauthor=true&cauthor_uid=14526109), [Alves G](http://www.ncbi.nlm.nih.gov/pubmed/?term=Alves%20G%5BAuthor%5D&cauthor=true&cauthor_uid=14526109), [Morillon R](http://www.ncbi.nlm.nih.gov/pubmed/?term=Morillon%20R%5BAuthor%5D&cauthor=true&cauthor_uid=14526109), [Maurel K](http://www.ncbi.nlm.nih.gov/pubmed/?term=Maurel%20K%5BAuthor%5D&cauthor=true&cauthor_uid=14526109), [Decourteix M](http://www.ncbi.nlm.nih.gov/pubmed/?term=Decourteix%20M%5BAuthor%5D&cauthor=true&cauthor_uid=14526109), [Guilliot A](http://www.ncbi.nlm.nih.gov/pubmed/?term=Guilliot%20A%5BAuthor%5D&cauthor=true&cauthor_uid=14526109), [Fleurat-Lessard P](http://www.ncbi.nlm.nih.gov/pubmed/?term=Fleurat-Lessard%20P%5BAuthor%5D&cauthor=true&cauthor_uid=14526109), [Julien JL](http://www.ncbi.nlm.nih.gov/pubmed/?term=Julien%20JL%5BAuthor%5D&cauthor=true&cauthor_uid=14526109), [Chrispeels MJ](http://www.ncbi.nlm.nih.gov/pubmed/?term=Chrispeels%20MJ%5BAuthor%5D&cauthor=true&cauthor_uid=14526109). Plasma membrane aquaporins are involved in winter embolism recovery in walnut tree. [Plant Physiol.](http://www.ncbi.nlm.nih.gov/pubmed/14526109) 2003; 133(2):630-41.
18. [Liu C](http://www.ncbi.nlm.nih.gov/pubmed/?term=Liu%20C%5BAuthor%5D&cauthor=true&cauthor_uid=22819861), [Li C](http://www.ncbi.nlm.nih.gov/pubmed/?term=Li%20C%5BAuthor%5D&cauthor=true&cauthor_uid=22819861), [Liang D](http://www.ncbi.nlm.nih.gov/pubmed/?term=Liang%20D%5BAuthor%5D&cauthor=true&cauthor_uid=22819861), [Wei Z](http://www.ncbi.nlm.nih.gov/pubmed/?term=Wei%20Z%5BAuthor%5D&cauthor=true&cauthor_uid=22819861), [Zhou S](http://www.ncbi.nlm.nih.gov/pubmed/?term=Zhou%20S%5BAuthor%5D&cauthor=true&cauthor_uid=22819861), [Wang R](http://www.ncbi.nlm.nih.gov/pubmed/?term=Wang%20R%5BAuthor%5D&cauthor=true&cauthor_uid=22819861), [Ma F](http://www.ncbi.nlm.nih.gov/pubmed/?term=Ma%20F%5BAuthor%5D&cauthor=true&cauthor_uid=22819861). Differential expression of ion transporters and aquaporins in leaves may contribute to different salt tolerance in Malus species. [Plant Physiol Biochem.](http://www.ncbi.nlm.nih.gov/pubmed/22819861) 2012; 58:159-65. doi: 10.1016/j.plaphy.2012.06.019.
19. [Vandeleur RK](http://www.ncbi.nlm.nih.gov/pubmed/?term=Vandeleur%20RK%5BAuthor%5D&cauthor=true&cauthor_uid=18987216), [Mayo G](http://www.ncbi.nlm.nih.gov/pubmed/?term=Mayo%20G%5BAuthor%5D&cauthor=true&cauthor_uid=18987216), [Shelden MC](http://www.ncbi.nlm.nih.gov/pubmed/?term=Shelden%20MC%5BAuthor%5D&cauthor=true&cauthor_uid=18987216), [Gilliham M](http://www.ncbi.nlm.nih.gov/pubmed/?term=Gilliham%20M%5BAuthor%5D&cauthor=true&cauthor_uid=18987216), [Kaiser BN](http://www.ncbi.nlm.nih.gov/pubmed/?term=Kaiser%20BN%5BAuthor%5D&cauthor=true&cauthor_uid=18987216), [Tyerman SD](http://www.ncbi.nlm.nih.gov/pubmed/?term=Tyerman%20SD%5BAuthor%5D&cauthor=true&cauthor_uid=18987216). The role of plasma membrane intrinsic protein aquaporins in water transport through roots: diurnal and drought stress responses reveal different strategies between isohydric and anisohydric cultivars of grapevine. Plant Physiol, 2008; 149(1):445-60. doi: 10.1104/pp.108.128645. Epub 2008 Nov 5.
20. Li DD, [Ruan XM](http://www.ncbi.nlm.nih.gov/pubmed/?term=Ruan%20XM%5BAuthor%5D&cauthor=true&cauthor_uid=23656428), [Zhang J](http://www.ncbi.nlm.nih.gov/pubmed/?term=Zhang%20J%5BAuthor%5D&cauthor=true&cauthor_uid=23656428), [Wu YJ](http://www.ncbi.nlm.nih.gov/pubmed/?term=Wu%20YJ%5BAuthor%5D&cauthor=true&cauthor_uid=23656428), [Wang XL](http://www.ncbi.nlm.nih.gov/pubmed/?term=Wang%20XL%5BAuthor%5D&cauthor=true&cauthor_uid=23656428), [Li XB](http://www.ncbi.nlm.nih.gov/pubmed/?term=Li%20XB%5BAuthor%5D&cauthor=true&cauthor_uid=23656428). Cotton plasma membrane intrinsic protein 2s (PIP2s) selectively interact to regulate their water channel activities and are required for fibre development. [New Phytol.](http://www.ncbi.nlm.nih.gov/pubmed/23656428) 2013; 199(3):695-707. doi: 10.1111/nph.12309.
21. [Navarro-Ródenas A](http://www.ncbi.nlm.nih.gov/pubmed/?term=Navarro-R%C3%B3denas%20A%5BAuthor%5D&cauthor=true&cauthor_uid=23656332), [Bárzana G](http://www.ncbi.nlm.nih.gov/pubmed/?term=B%C3%A1rzana%20G%5BAuthor%5D&cauthor=true&cauthor_uid=23656332), [Nicolás E](http://www.ncbi.nlm.nih.gov/pubmed/?term=Nicol%C3%A1s%20E%5BAuthor%5D&cauthor=true&cauthor_uid=23656332), [Carra A](http://www.ncbi.nlm.nih.gov/pubmed/?term=Carra%20A%5BAuthor%5D&cauthor=true&cauthor_uid=23656332), [Schubert A](http://www.ncbi.nlm.nih.gov/pubmed/?term=Schubert%20A%5BAuthor%5D&cauthor=true&cauthor_uid=23656332), [Morte A](http://www.ncbi.nlm.nih.gov/pubmed/?term=Morte%20A%5BAuthor%5D&cauthor=true&cauthor_uid=23656332). Expression analysis of aquaporins from desert truffle mycorrhizal symbiosis reveals a fine-tuned regulation under drought. [Mol Plant Microbe Interact.](http://www.ncbi.nlm.nih.gov/pubmed/23656332) 2013; 26(9):1068-78. doi: 10.1094/MPMI-07-12-0178-R.
