# Supplementary material for: Genome-wide expression profiling of aquaporin genes confer responses to abiotic and biotic stresses in Brassica rapa
Source: BMC Plant Biol. 2017 Jan 25;17:23. doi: 10.1186/s12870-017-0979-5 (PMC5264328; doi:10.1186/s12870-017-0979-5)
Supplement: Additional file 3: Figure S1. — Alignment of amino acid sequences of Arabidopsis and (1a) BrNIP (1b) BrSIP (1c) BrPIP and (1d) BrTIP subfamily members. Upper red line indicates predicted MIP domain and the blue portion of the alignment denotes predicted transmembrane domains. The two conserved NPA motifs are shown in bold pink letters. Residues comprising the ar/R filter are marked in yellow and labelled H2, H5, LE1 and LE2. Residues occupying the conserved Froger’s positions one to five (from N- to C-terminus P1 to P5) are marked in green. (PPTX 437 kb) [file 12870_2017_979_MOESM3_ESM.pptx]

## Slide 1
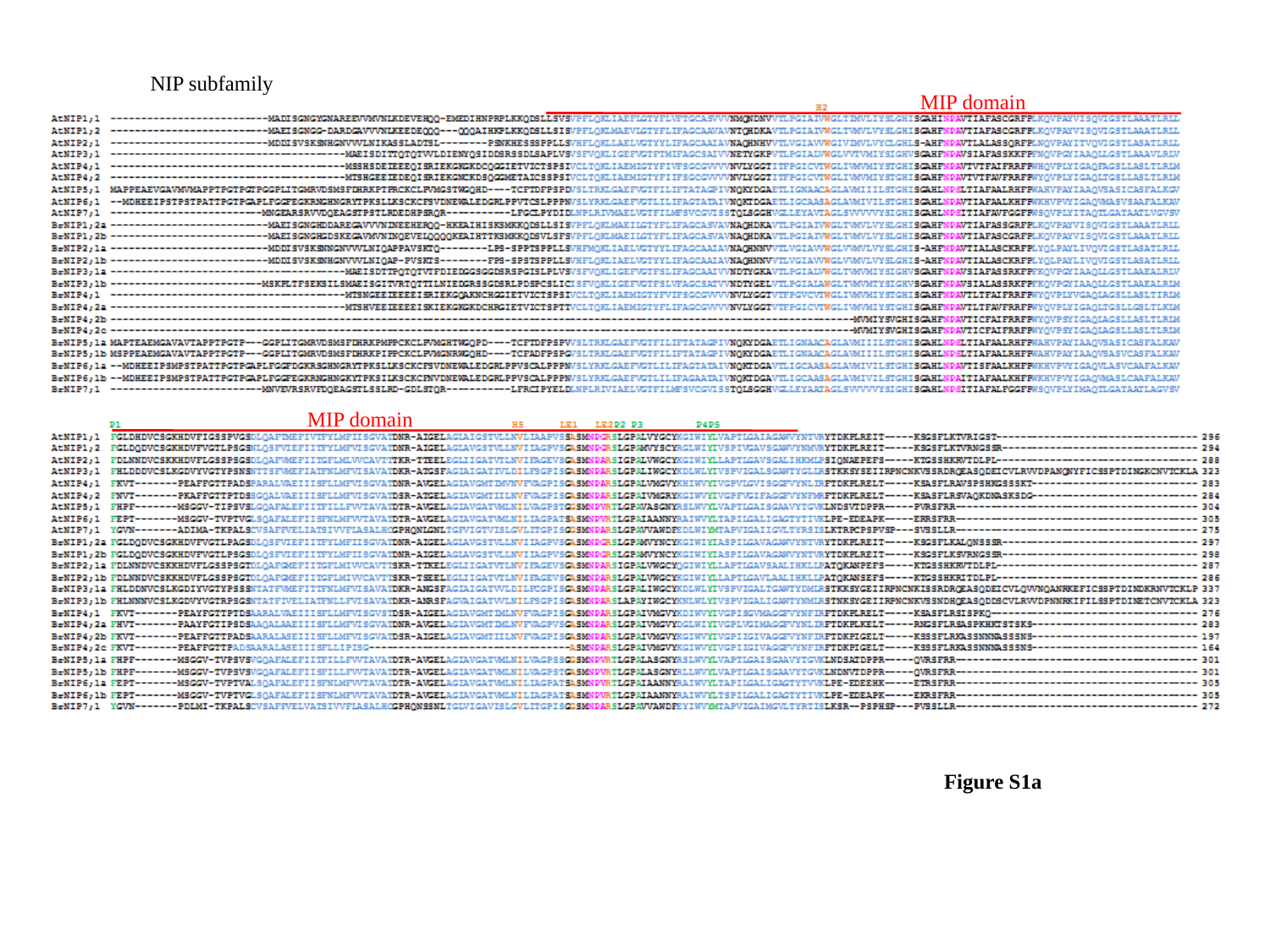

NIP subfamily
MIP domain
MIP domain
Figure S1a

## Slide 2
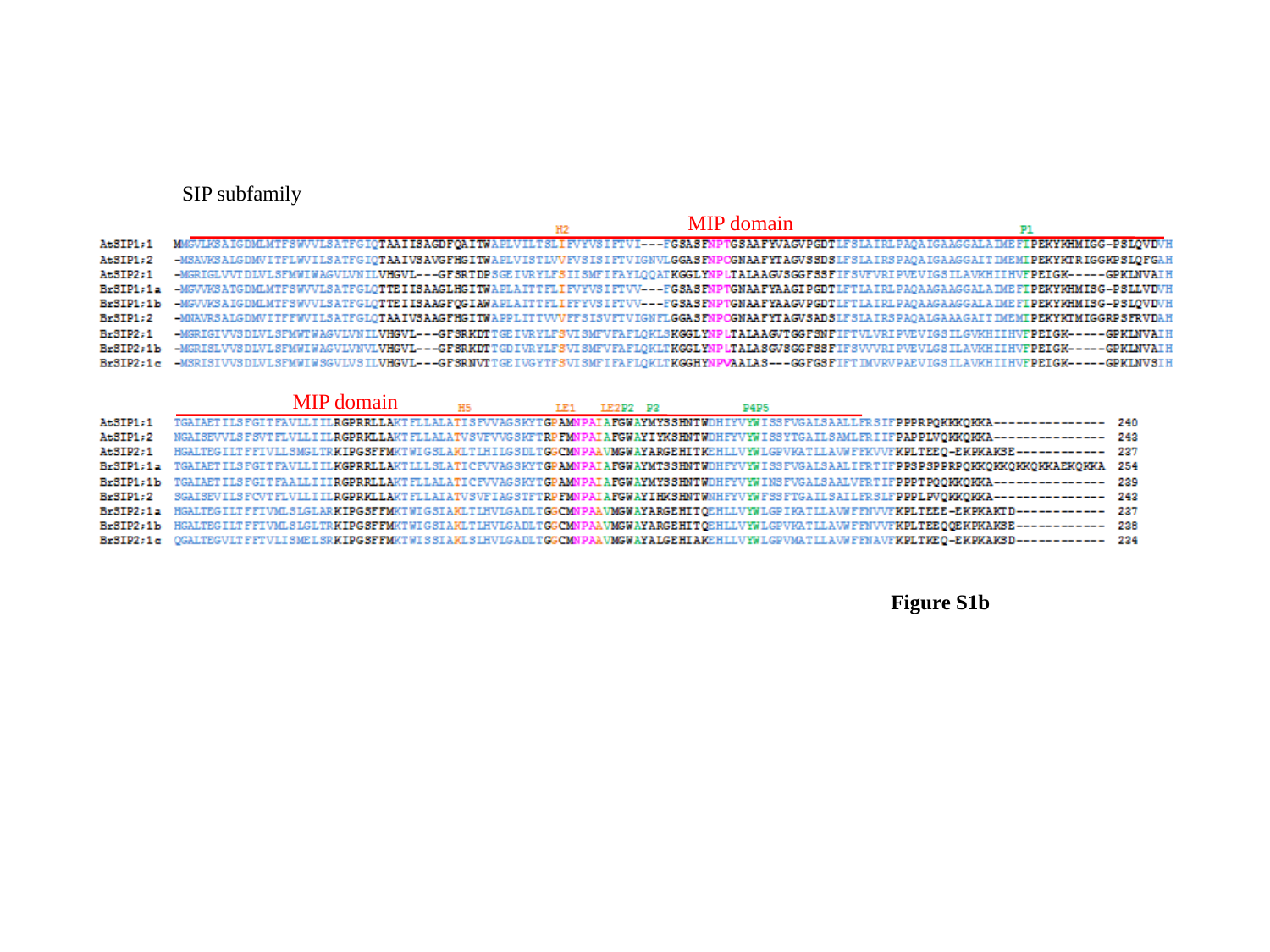

SIP subfamily
MIP domain
MIP domain
Figure S1b

## Slide 3
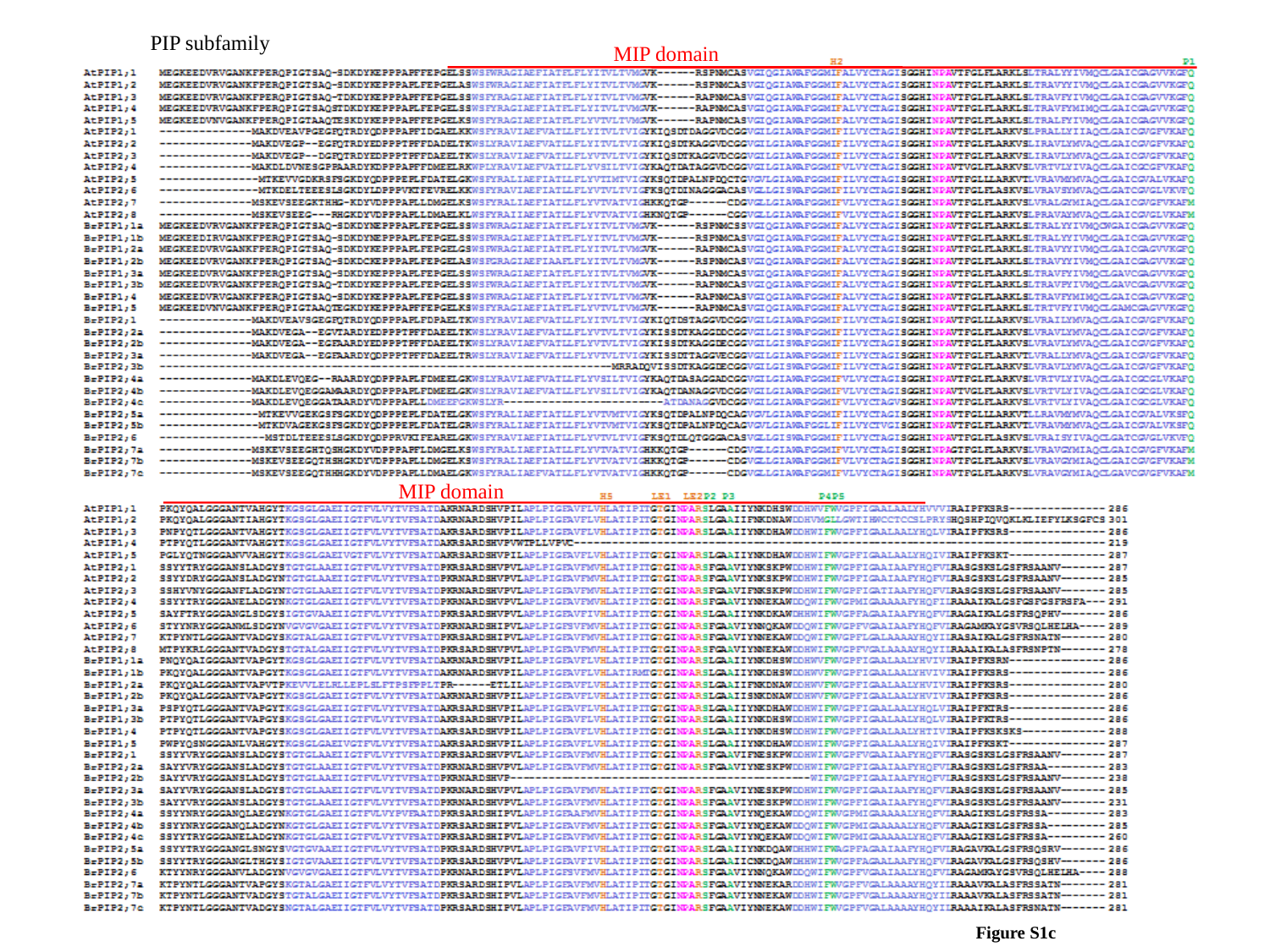

PIP subfamily
MIP domain
MIP domain
Figure S1c

## Slide 4
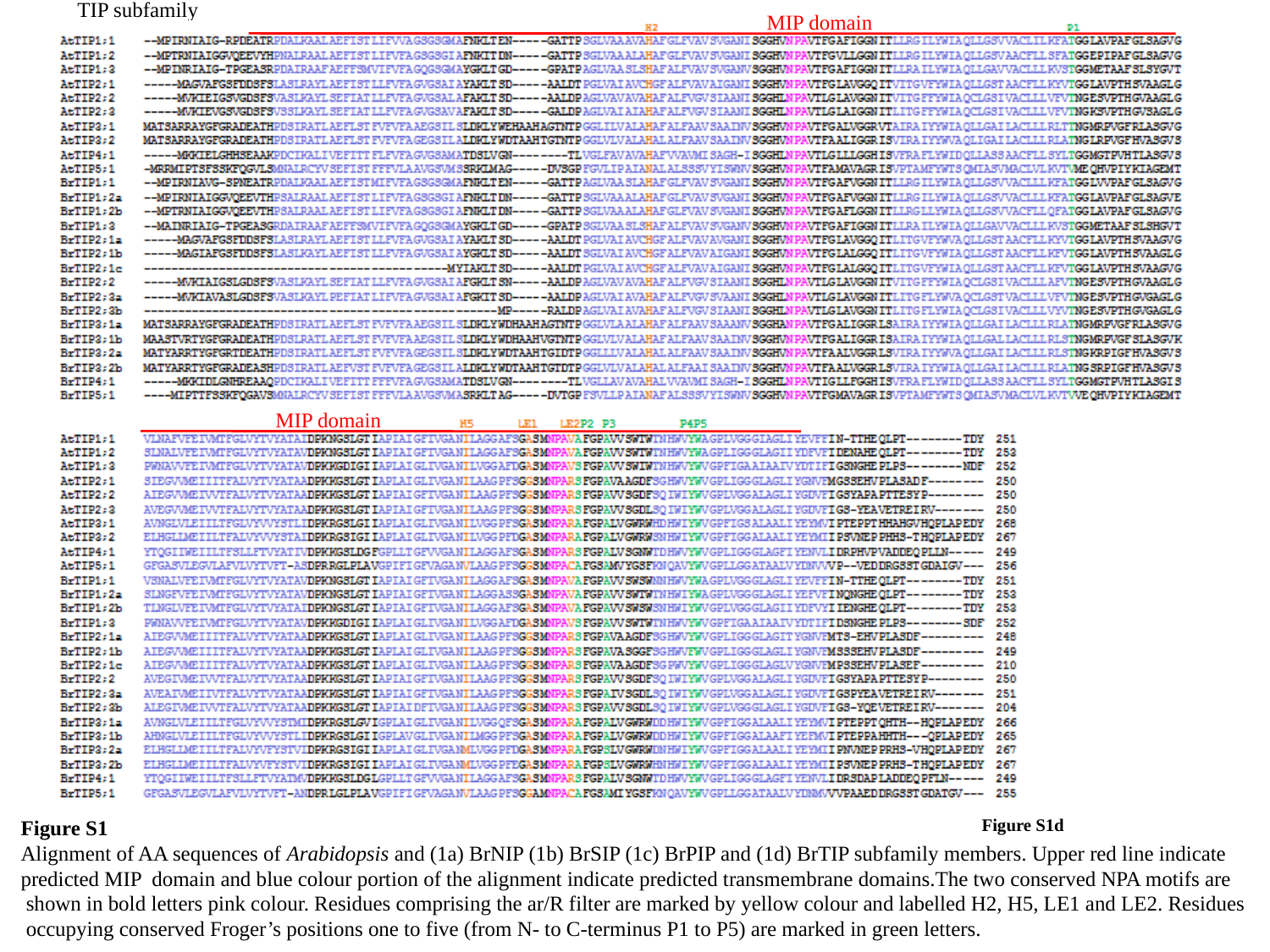

TIP subfamily
MIP domain
MIP domain
Figure S1
Alignment of AA sequences of Arabidopsis and (1a) BrNIP (1b) BrSIP (1c) BrPIP and (1d) BrTIP subfamily members. Upper red line indicate
predicted MIP domain and blue colour portion of the alignment indicate predicted transmembrane domains.The two conserved NPA motifs are
 shown in bold letters pink colour. Residues comprising the ar/R filter are marked by yellow colour and labelled H2, H5, LE1 and LE2. Residues
 occupying conserved Froger’s positions one to five (from N- to C-terminus P1 to P5) are marked in green letters.
Figure S1d
