# Supplementary figures and images for: Genome-wide expression profiling of aquaporin genes confer responses to abiotic and biotic stresses in Brassica rapa
Source: BMC Plant Biol. 2017 Jan 25;17:23. doi: 10.1186/s12870-017-0979-5 (PMC5264328; doi:10.1186/s12870-017-0979-5)

## Slide 1
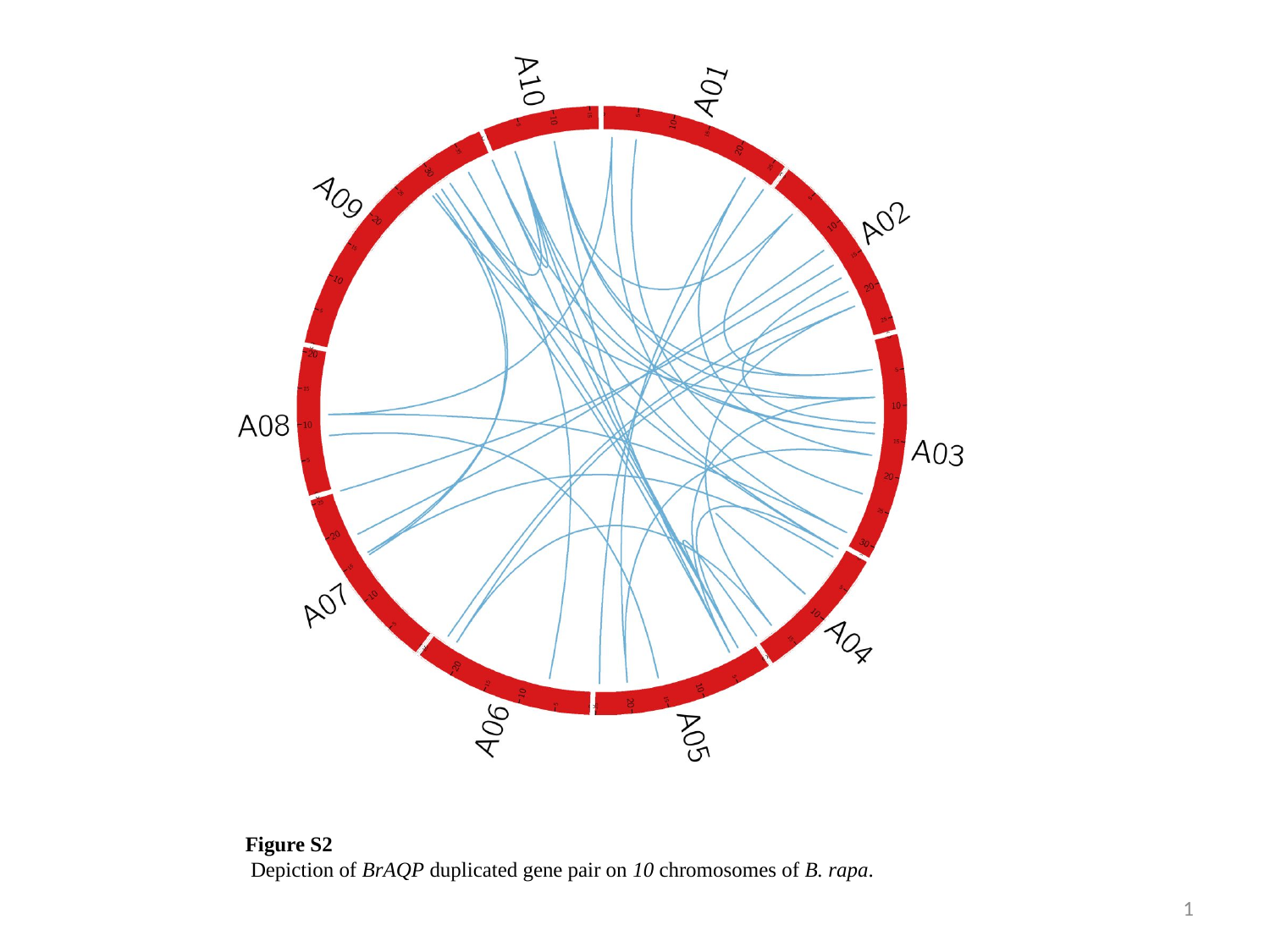

Figure S2
 Depiction of BrAQP duplicated gene pair on 10 chromosomes of B. rapa.
1

Supplement: Additional file 5: Figure S2. — Depiction of BrAQP duplicated gene pairs on 10 chromosomes of B. rapa. (PPTX 400 kb) [file 12870_2017_979_MOESM5_ESM.pptx]
