# Supplementary material for: Genome-wide expression profiling of aquaporin genes confer responses to abiotic and biotic stresses in Brassica rapa
Source: BMC Plant Biol. 2017 Jan 25;17:23. doi: 10.1186/s12870-017-0979-5 (PMC5264328; doi:10.1186/s12870-017-0979-5)
Supplement: Additional file 6: Figure S3. — Schematic representation of motif compositions in the BrAQP protein sequences. Different motifs, numbered 1–10, are displayed in different colored boxes. The names of all members are displayed on the left, while the length of the motif is shown in the scale at the bottom of the figure. (PPTX 269 kb) [file 12870_2017_979_MOESM6_ESM.pptx]

## Slide 1
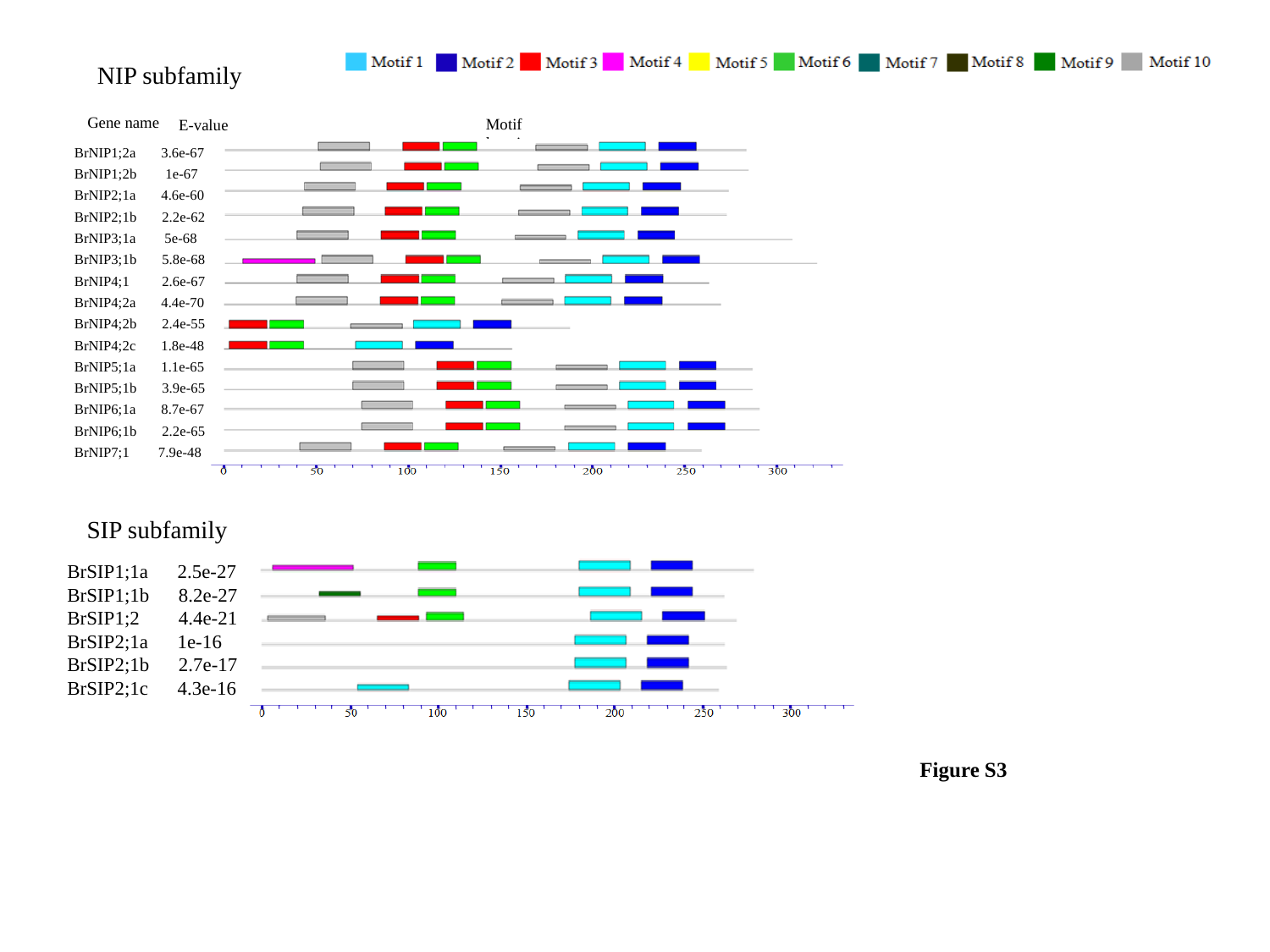

NIP subfamily
Gene name
Motif location
E-value
BrNIP1;2a 3.6e-67
BrNIP1;2b 1e-67
BrNIP2;1a 4.6e-60
BrNIP2;1b 2.2e-62
BrNIP3;1a 5e-68
BrNIP3;1b 5.8e-68
BrNIP4;1 2.6e-67
BrNIP4;2a 4.4e-70
BrNIP4;2b 2.4e-55
BrNIP4;2c 1.8e-48
BrNIP5;1a 1.1e-65
BrNIP5;1b 3.9e-65
BrNIP6;1a 8.7e-67
BrNIP6;1b 2.2e-65
BrNIP7;1 7.9e-48
SIP subfamily
BrSIP1;1a 2.5e-27
BrSIP1;1b 8.2e-27
BrSIP1;2 4.4e-21
BrSIP2;1a 1e-16
BrSIP2;1b 2.7e-17
BrSIP2;1c 4.3e-16
Figure S3

## Slide 2
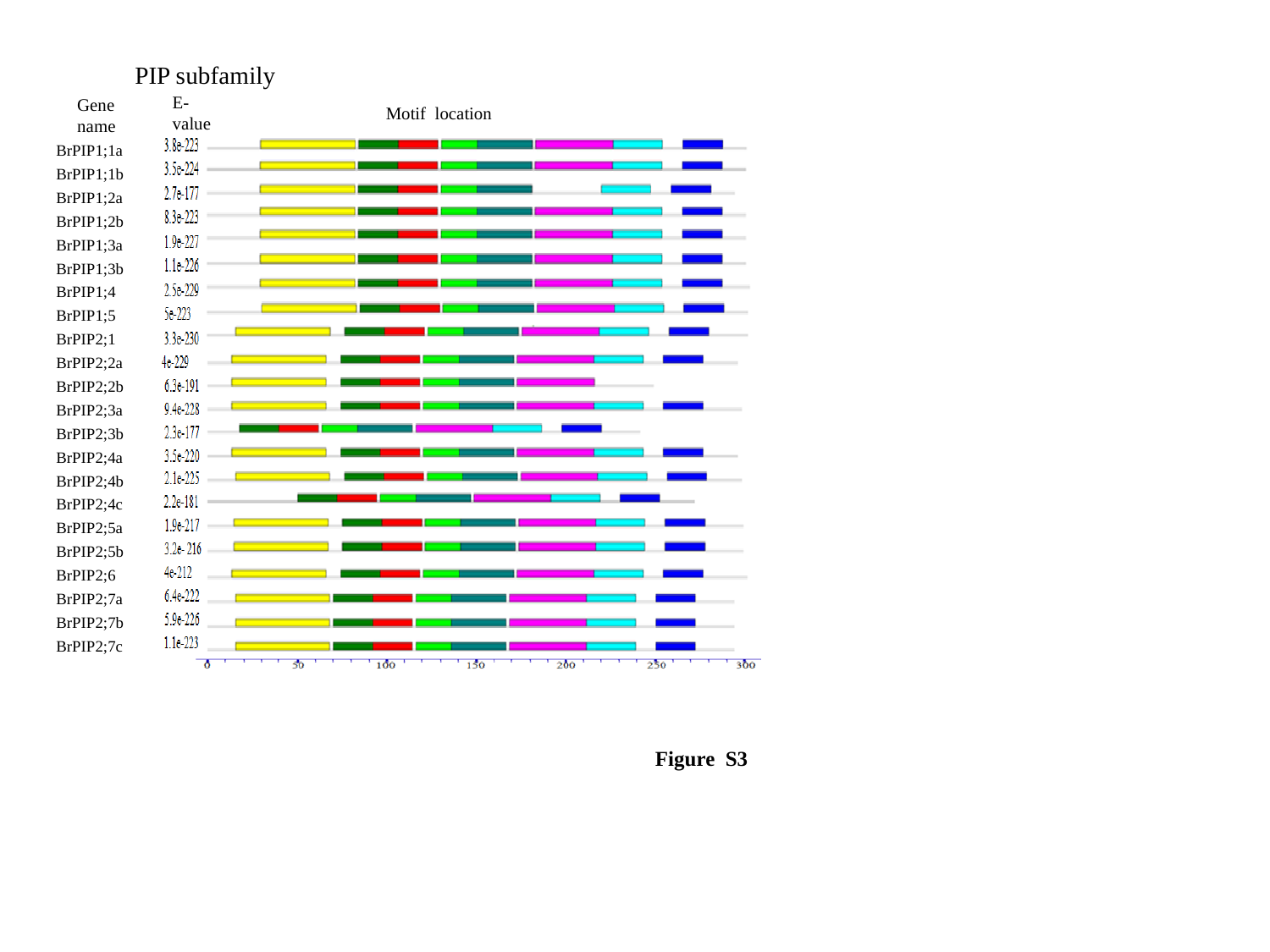

PIP subfamily
E-value
Gene name
Motif location
BrPIP1;1a
BrPIP1;1b
BrPIP1;2a
BrPIP1;2b
BrPIP1;3a
BrPIP1;3b
BrPIP1;4
BrPIP1;5
BrPIP2;1
BrPIP2;2a
BrPIP2;2b
BrPIP2;3a
BrPIP2;3b
BrPIP2;4a
BrPIP2;4b
BrPIP2;4c
BrPIP2;5a
BrPIP2;5b
BrPIP2;6
BrPIP2;7a
BrPIP2;7b
BrPIP2;7c
Figure S3

## Slide 3
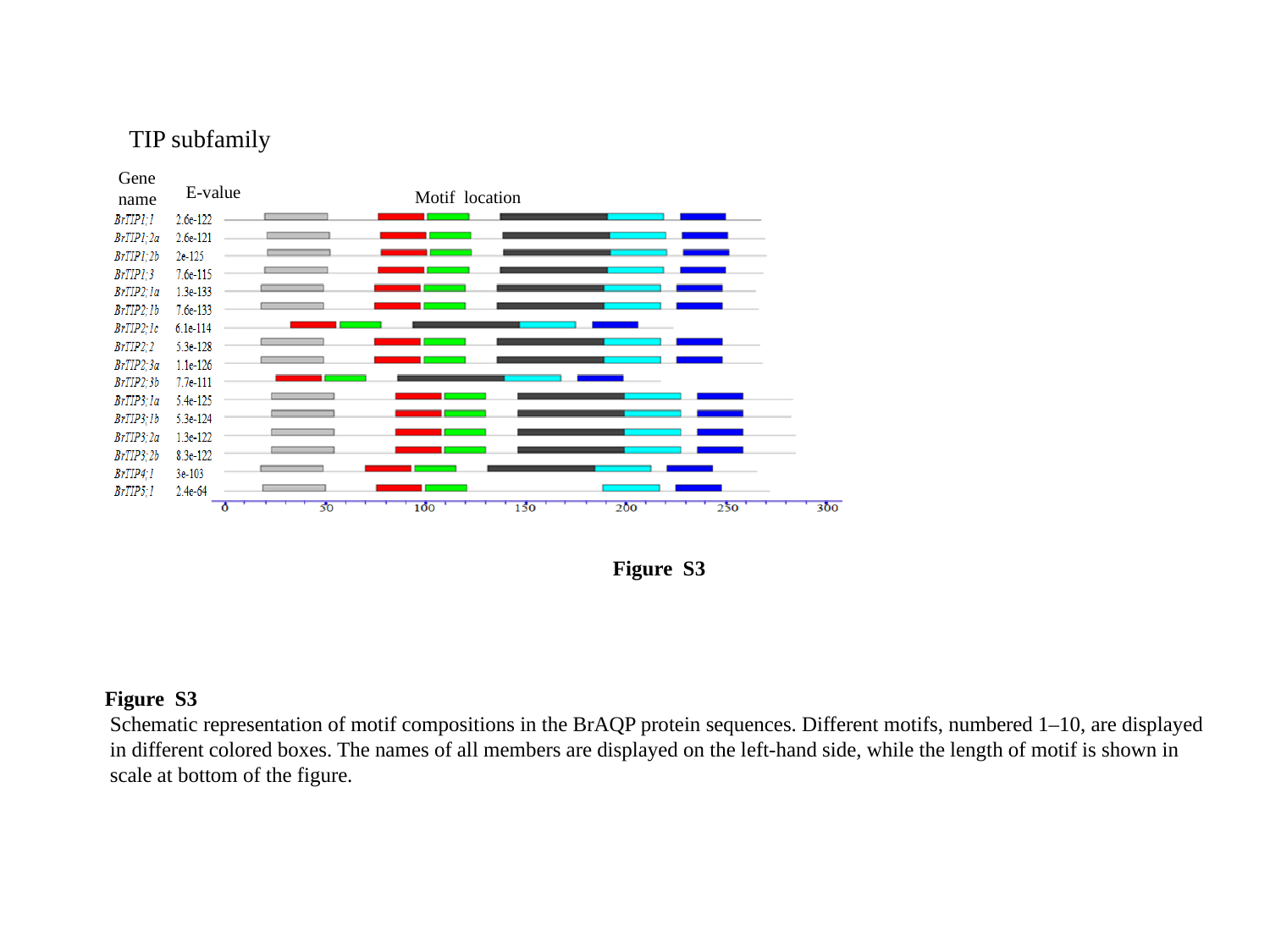

TIP subfamily
Gene name
E-value
Motif location
Figure S3
Figure S3
 Schematic representation of motif compositions in the BrAQP protein sequences. Different motifs, numbered 1–10, are displayed
 in different colored boxes. The names of all members are displayed on the left-hand side, while the length of motif is shown in
 scale at bottom of the figure.
