# Supplementary material for: Genome-wide expression profiling of aquaporin genes confer responses to abiotic and biotic stresses in Brassica rapa
Source: BMC Plant Biol. 2017 Jan 25;17:23. doi: 10.1186/s12870-017-0979-5 (PMC5264328; doi:10.1186/s12870-017-0979-5)
Supplement: Additional file 7: Table S4. — Best possible match sequences of motifs (1–15) presented in Additional file Figure S3. (DOCX 11 kb) [file 12870_2017_979_MOESM7_ESM.docx]

**Table S4.** Best possible match sequences of motifs (1-10) presented in Supplementary Figure 3.

| Motif | Width | Best possible match |
| --- | --- | --- |
| 1 | 26 | GFAVFMNHLATIPITGTSMNPARSFG |
| 2 | 21 | DDHWIYWVGPFIGAALAAWYH |
| 3 | 21 | VYCTAHISGGHINPAVTFGLF |
| 4 | 41 | GTGLGAEIIGTFVLVYTVFSATDPKRNARDSHVPVLAPLPI |
| 5 | 50 | KDYQDPPPAPLFDMEELKKWSFYRAVIAEFIATLLFLYVTVLTVMGYKRQ |
| 6 | 19 | RKFSLKRAPYYWIAQCLGS |
| 7 | 29 | ICGCGFVKAFQPSYYQRYGGGANTLADGY |
| 8 | 50 | VPTHHVAAGVGALEGLVMEIIMTFALVYTVYATAIDPKKGSLGTIAPLAI |
| 9 | 21 | MCDSVGIQGIAWAFGGMIFIL |
| 10 | 29 | LRKYLAEFISTYIFVFAGCGSIMVNDKYD |
