# Supplementary material for: Genome-wide expression profiling of aquaporin genes confer responses to abiotic and biotic stresses in Brassica rapa
Source: BMC Plant Biol. 2017 Jan 25;17:23. doi: 10.1186/s12870-017-0979-5 (PMC5264328; doi:10.1186/s12870-017-0979-5)
Supplement: Additional file 8: Figure S4. — The intron-exon structures of BrAQP genes. Names of the genes are on the left. The thick blue lines, exons; fine red lines, introns. (PPTX 346 kb) [file 12870_2017_979_MOESM8_ESM.pptx]

## Slide 1
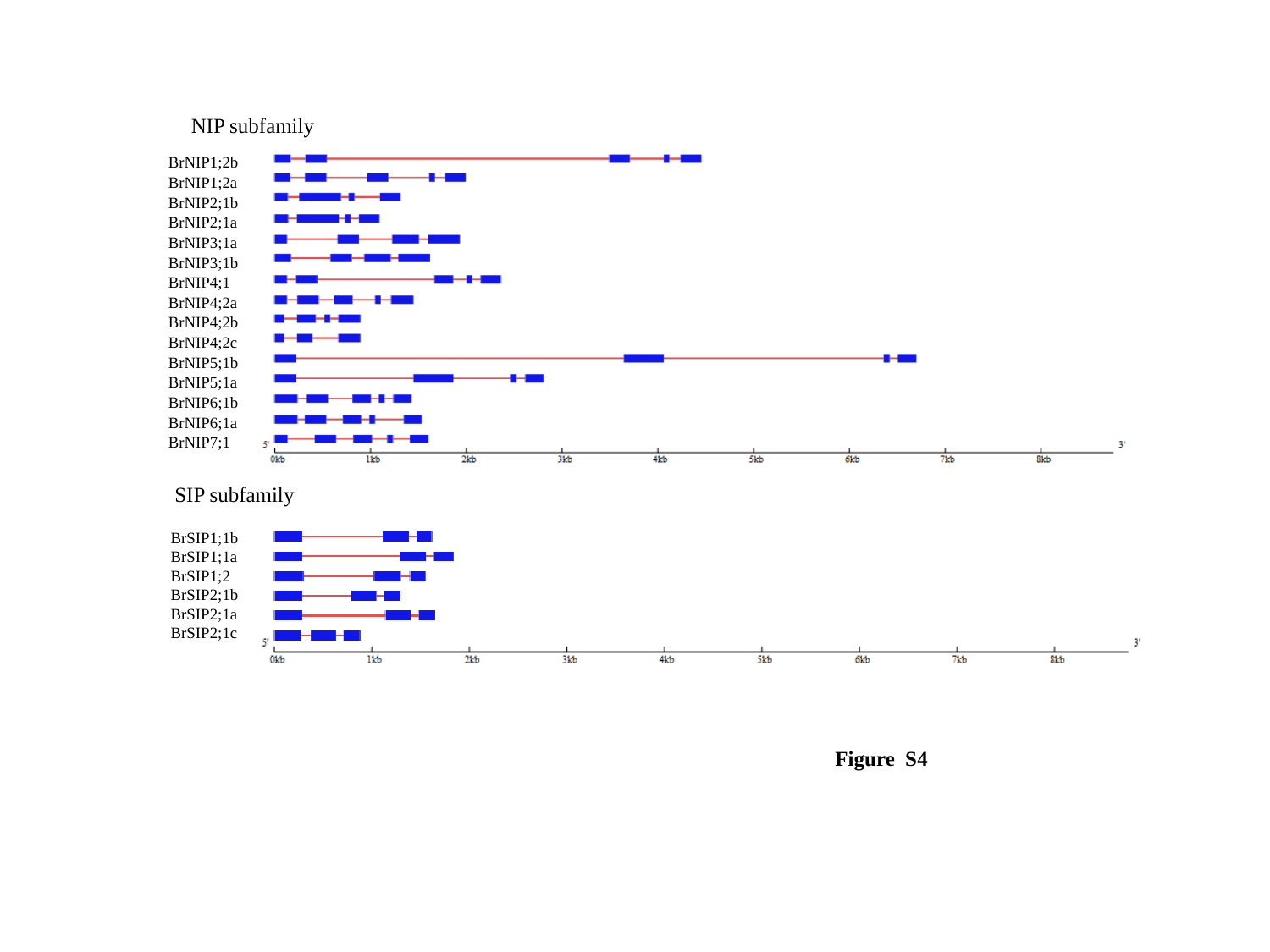

NIP subfamily
BrNIP1;2b
BrNIP1;2a
BrNIP2;1b
BrNIP2;1a
BrNIP3;1a
BrNIP3;1b
BrNIP4;1
BrNIP4;2a
BrNIP4;2b
BrNIP4;2c
BrNIP5;1b
BrNIP5;1a
BrNIP6;1b
BrNIP6;1a
BrNIP7;1
SIP subfamily
BrSIP1;1b
BrSIP1;1a
BrSIP1;2
BrSIP2;1b
BrSIP2;1a
BrSIP2;1c
Figure S4

## Slide 2
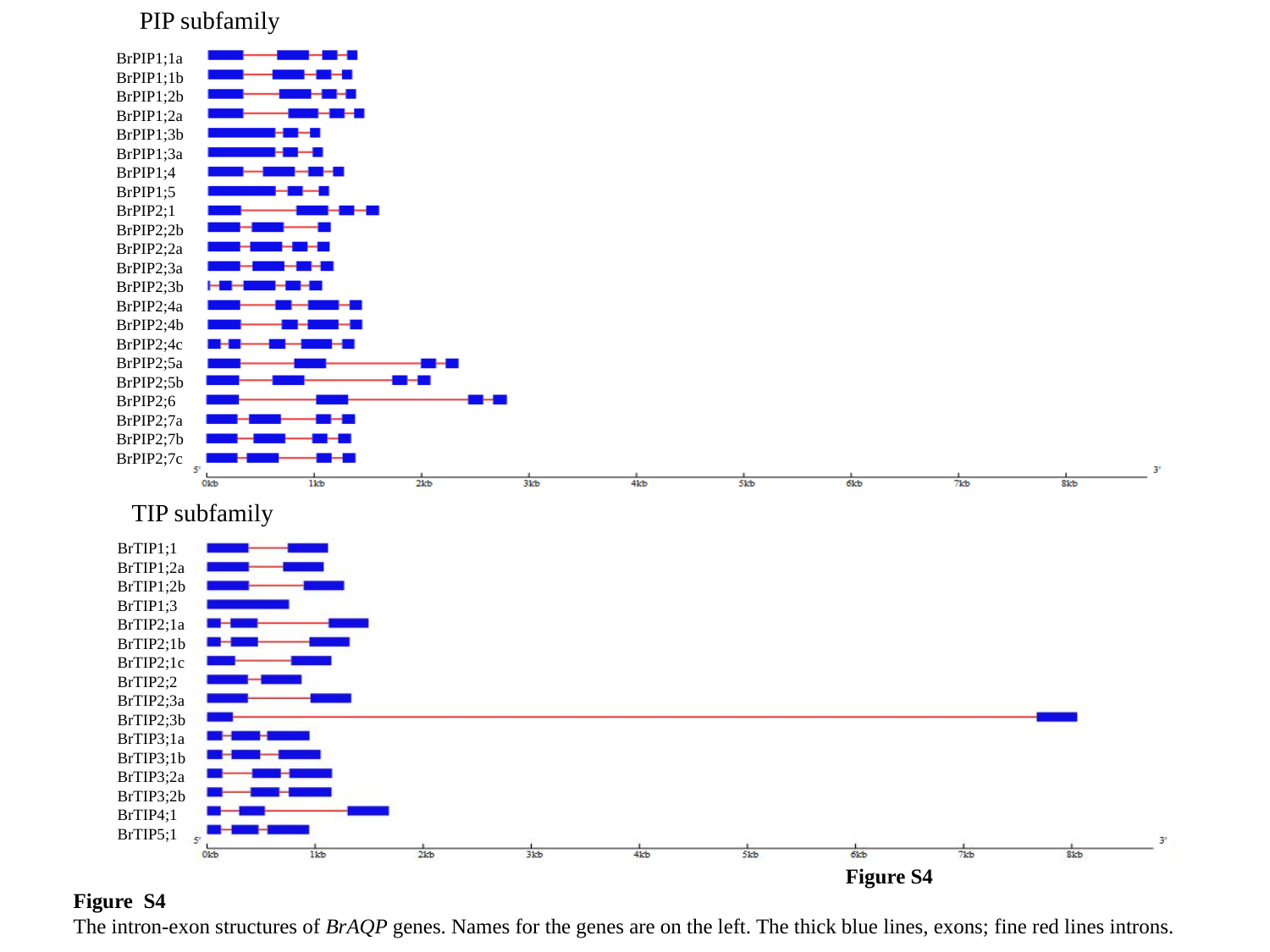

PIP subfamily
BrPIP1;1a
BrPIP1;1b
BrPIP1;2b
BrPIP1;2a
BrPIP1;3b
BrPIP1;3a
BrPIP1;4
BrPIP1;5
BrPIP2;1
BrPIP2;2b
BrPIP2;2a
BrPIP2;3a
BrPIP2;3b
BrPIP2;4a
BrPIP2;4b
BrPIP2;4c
BrPIP2;5a
BrPIP2;5b
BrPIP2;6
BrPIP2;7a
BrPIP2;7b
BrPIP2;7c
TIP subfamily
BrTIP1;1
BrTIP1;2a
BrTIP1;2b
BrTIP1;3
BrTIP2;1a
BrTIP2;1b
BrTIP2;1c
BrTIP2;2
BrTIP2;3a
BrTIP2;3b
BrTIP3;1a
BrTIP3;1b
BrTIP3;2a
BrTIP3;2b
BrTIP4;1
BrTIP5;1
Figure S4
Figure S4
The intron-exon structures of BrAQP genes. Names for the genes are on the left. The thick blue lines, exons; fine red lines introns.
