# Supplementary material for: Genome-wide expression profiling of aquaporin genes confer responses to abiotic and biotic stresses in Brassica rapa
Source: BMC Plant Biol. 2017 Jan 25;17:23. doi: 10.1186/s12870-017-0979-5 (PMC5264328; doi:10.1186/s12870-017-0979-5)
Supplement: Additional file 9: Figure S5. — Expression profiles of BrPIP genes in various tissues as determined by RT-PCR analyses. Four amplified bands from left to right for each gene represent amplified products from R, roots; S, stems; L, leaves; Fb, flower buds (PPTX 103 kb) [file 12870_2017_979_MOESM9_ESM.pptx]

## Slide 1
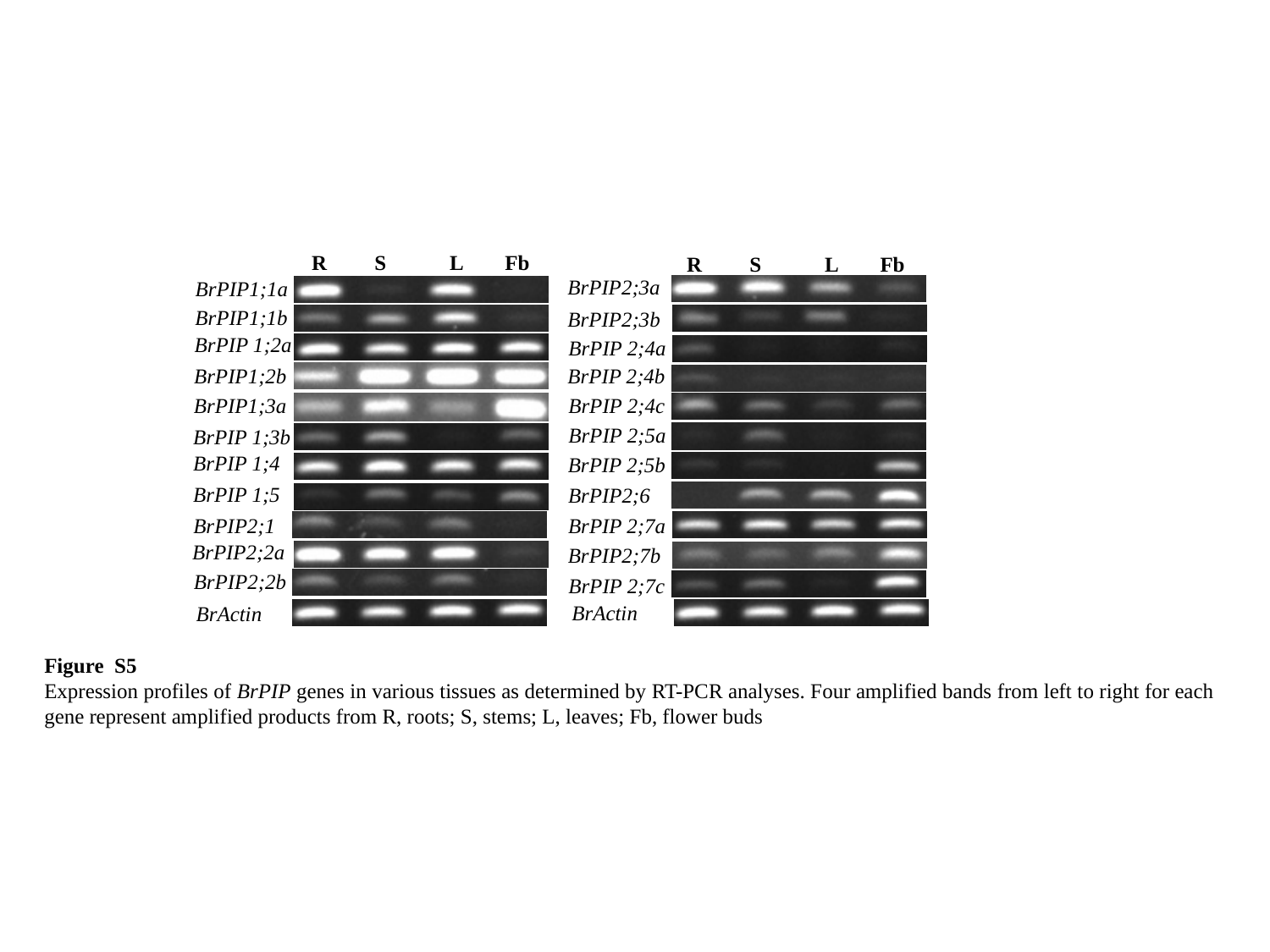

R S L Fb
 R S L Fb
BrPIP2;3a
BrPIP1;1a
BrPIP1;1b
BrPIP2;3b
BrPIP 1;2a
BrPIP 2;4a
BrPIP 2;4b
BrPIP1;2b
BrPIP1;3a
BrPIP 2;4c
BrPIP 2;5a
BrPIP 1;3b
BrPIP 1;4
BrPIP 2;5b
BrPIP 1;5
BrPIP2;6
BrPIP 2;7a
BrPIP2;1
BrPIP2;2a
BrPIP2;7b
BrPIP2;2b
BrPIP 2;7c
BrActin
BrActin
Figure S5
Expression profiles of BrPIP genes in various tissues as determined by RT-PCR analyses. Four amplified bands from left to right for each gene represent amplified products from R, roots; S, stems; L, leaves; Fb, flower buds
