# Supplementary material for: Genome-wide expression profiling of aquaporin genes confer responses to abiotic and biotic stresses in Brassica rapa
Source: BMC Plant Biol. 2017 Jan 25;17:23. doi: 10.1186/s12870-017-0979-5 (PMC5264328; doi:10.1186/s12870-017-0979-5)
Supplement: Additional file 10: Table S5. — Primer sequence used for real time and RT-PCR amplification of Aquaporin genes of B. rapa. (DOCX 12 kb) [file 12870_2017_979_MOESM10_ESM.docx]

**Table S5.** Primer sequence used for real time and RT-PCR amplification of *Auaporin* genes of *B. rapa*

| Gene name | Forward | Reverse |
| --- | --- | --- |
| BrPIP1;1a | AGAGCCCTGTACTACATAGTG | AGTGGCTGAGAAGACTGTG |
| BrPIP1;1b | CCGGAAGTTGTCGCTCAC | AGTGTACACAAGGACGAATGTG |
| BrPIP1;2b | GGATCGCCGAGTTCATCG | AGCTTTCTTGCCAAGAACAG |
| BrPIP1;2a | TTCCGGAGAGACAACCCATC | TGTTCGGTGCCCTCTTCAC |
| BrPIP1;3b | TTCCTTTACATAACGGTGCT | GAGCTAAGAACAGACCGAAA |
| BrPIP1;3a | TGACTGTAATGGGAGTGAAGAG | AGAAGACAGCTCTGGTCAGTG |
| BrPIP1;4 | CCGATATTGGCACCGCTAC | GTGTGATAGAGTGCTGCTAG |
| BrPIP1;5 | ACAAAGGGTTCAGGTCTTGG | TCCTGGCCGGGTTAATTCC |
| BrPIP2;1 | CTACTCTTGGCACGTAAAGTG | GAACGAAAGTACCAATGATCTC |
| BrPIP2;2b | CTCCTCTTCTTGTATGTCACTG | ACGATACCTTCCTTGCCAAG |
| BrPIP2;2a | GGAAGGAGCAGAGGGAGTC | GTCGCCTCCTGCCTTAGTG |
| BrPIP2;3a | ATCCTCGGCATCGCTTGG | AAAGGCCTTTACGAACCCAAC |
| BrPIP2;3b | GGATTTGCCGTGTTCATGG | AAGGACCCGAGGGACTTG |
| BrPIP2;4a | CGGTCTGGTTAAAGCGTTCC | CGCCAAAACAGGAATATGAG |
| BrPIP2;4b | AAGAGGGCGGAGCAATGG | CAACTCCTCCGGCATTTGC |
| BrPIP2;4c | ACGTTCGGATTGTTCTTAGC | ATGATCTCCGCACCGAGTC |
| BrPIP2;5a | GGTGACATTTGGACTATTGC | GCAACACCAGTGCCAACAC |
| BrPIP2;5b | CATATAAACCCGGCCGTGAC | GCCGATGCTATAACCATGTG |
| BrPIP2;6 | TATACGTCACCGTCTTGACA | GCTAGCCAAGAATAGTCCAA |
| BrPIP2;7a | TTGTGAGAGCTGTTGGGTAC | CGGAGAAGACGGTGTAAACG |
| BrPIP2;7b | GGTGCATTTGGCAACCATC | GAGGCCAAGGCCTTAACTG |
| BrPIP2;7c | CCACCACCATGGCAAAGAC | GATCCCGAGCAAACCAACG |
